# Conserved phosphoryl transfer mechanisms within kinase families and the role of the C8 proton of ATP in the activation of phosphoryl transfer

**DOI:** 10.1186/1756-0500-5-131

**Published:** 2012-03-08

**Authors:** Colin P Kenyon, Robyn L Roth, Chris W van der Westhuyzen, Christopher J Parkinson

**Affiliations:** 1 CSIR, Biosciences, Meiring-Naude Road, Pretoria, 0001 Gauteng, South Africa

## Abstract

**Background:**

The kinome is made up of a large number of functionally diverse enzymes, with the classification indicating very little about the extent of the conserved kinetic mechanisms associated with phosphoryl transfer. It has been demonstrated that C8-H of ATP plays a critical role in the activity of a range of kinase and synthetase enzymes.

**Results:**

A number of conserved mechanisms within the prescribed kinase fold families have been identified directly utilizing the C8-H of ATP in the initiation of phosphoryl transfer. These mechanisms are based on structurally conserved amino acid residues that are within hydrogen bonding distance of a co-crystallized nucleotide. On the basis of these conserved mechanisms, the role of the nucleotide C8-H in initiating the formation of a pentavalent intermediate between the γ-phosphate of the ATP and the substrate nucleophile is defined. All reactions can be clustered into two mechanisms by which the C8-H is induced to be labile via the coordination of a backbone carbonyl to C6-NH_2 _of the adenyl moiety, namely a "push" mechanism, and a "pull" mechanism, based on the protonation of N7. Associated with the "push" mechanism and "pull" mechanisms are a series of proton transfer cascades, initiated from C8-H, via the tri-phosphate backbone, culminating in the formation of the pentavalent transition state between the γ-phosphate of the ATP and the substrate nucleophile.

**Conclusions:**

The "push" mechanism and a "pull" mechanism are responsible for inducing the C8-H of adenyl moiety to become more labile. These mechanisms and the associated proton transfer cascades achieve the proton transfer via different family-specific conserved sets of amino acids. Each of these mechanisms would allow for the regulation of the rate of formation of the pentavalent intermediate between the ATP and the substrate nucleophile. Phosphoryl transfer within kinases is therefore a specific event mediated and regulated via the coordination of the adenyl moiety of ATP and the C8-H of the adenyl moiety.

## Background

The kinases are a large number of structurally diverse enzymes that play a critical role in numerous metabolic and signalling pathways and whose substrates may be a small molecule, lipid, or protein. The International Union of Pure and Applied Chemistry and the International Union of Biochemistry (IUPAC/IUB) commission on the classification and nomenclature of enzymes placed the enzymes that transfer high energy phosphate bonds from nucleotides into two divisions: the transferases (kinases) and the ligases (synthetases) [1]. The transferases have been placed in Division 2 and the ligases into Division 6 of the Enzyme Commission (EC) classification. The ligases catalyse the joining of two molecules with the concomitant hydrolysis of the pyrophosphate bond of ATP, while a kinase is defined as an enzyme which catalyses the transfer of the phosphate group from ATP (or GTP) to a substrate containing an alcohol, amino, carboxyl, or phosphate group as the phosphoryl acceptor [2,3]. The kinases have been classified into 25 families of homologous proteins, with the families assembled into 12 fold-groups based on the similarity of their structural folds [2,3]. This classification relays little information on the catalytic mechanisms employed in nucleotide binding and phosphoryl transfer. The critical question that needed to be answered was to what extent the functionality required for the catalysis of phosphoryl transfer is conserved within the 25 families or 12 fold-groups.

Furthermore, during research carried out on the effect of deuteration at the C8 position of ATP on the activity of a number of kinase and synthetase enzymes, it became evident that the C8 proton of ATP plays a direct mechanistic role in initiating phosphoryl transfer, and probably also in the regulation of catalysis via the regulation of the rate of reaction [4]. The Kinetic Isotope Effect (KIE) obtained in enzymes utilizing ATP deuterated at C8 was found to be significantly in excess of 2 and in most cases in excess of 10 at low ATP concentrations. *This is a primary effect and is therefore dependent on C8-H bond breaking*. To this end, the putative mechanistic role of the C8 proton in the catalysis of the 22 families within the 10-fold groups was also investigated in the context of the conserved catalytic residues of each group of kinases.

**Figure 1:**
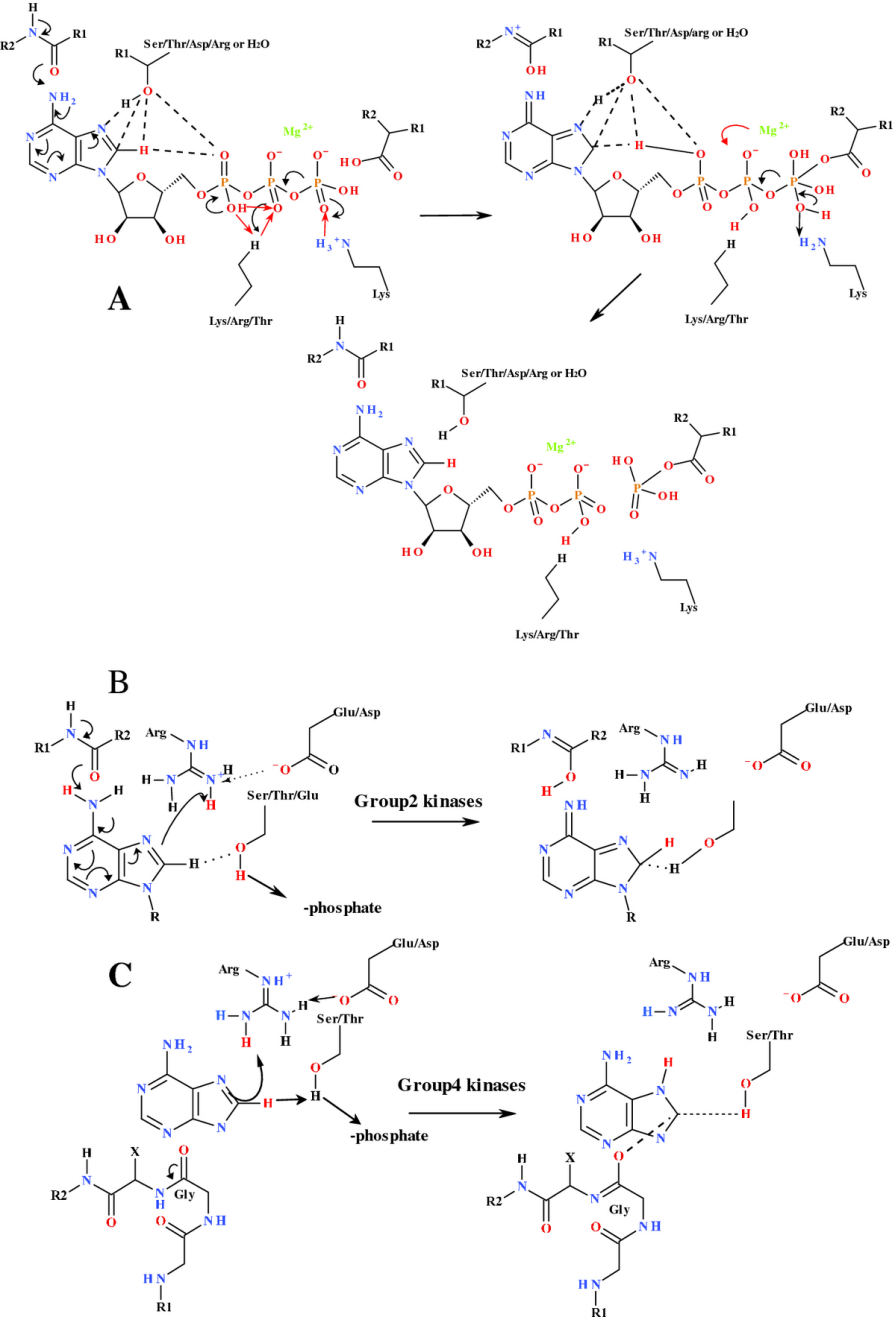
**A. General mechanism for the induction of phosphoryl transfer from the C8H of the adenyl moiety**. This is initiated via the coordination of the ATP adenyl moiety and effective proton transfer along the phosphate tail of ATP initiated from C8-H and mediated via range of conserved coordinating amino acid chains that are specific for each kinase group and family. As the KIE is a primary effect and is therefore dependent on C8-H bond breaking the C8-H is rendered more acidic by either of two mechanisms, a "push" mechanism mediated by the interaction of a backbone carbonyl at the C6-NH_2 _or a "pull" mechanism mediated by the interaction via coordination of a proton donor at N7 or hydrogen bonding of C8-H to an adjacent carbonyl or alcohol (or acceptor group). The red arrows indicate direction of proton migration during the reaction. B. The "push" mechanism is initiated with the redistribution of electron density in the adenyl ring mediated by the coordination of the backbone carbonyl to C6-NH_2 _via a high energy adenosine tautomer. C. The "pull" mechanism is mediated via the formation of a carbenoid species induced via the protonation of N7.

There are two accepted mechanisms by which catalysis might occur in nucleophilic substitution reactions: either by generation/formation of a leaving group or by the activation of the nucleophile, or both. Chemically phosphoryl transfer could occur by either by an S_N_1 or S_N_2 type reaction mechanism. However, within enzyme-catalysed systems it appears to occur predominantly via an in-line associative S_N_2 type mechanism [5]. As the C8-H appears to play a direct role in initiating phosphoryl transfer [4], the main aim of this investigation was to establish mechanistically how this might occur within the 22 families or 12 fold-groups of the kinase enzymes, and also the extent to which these mechanisms might be conserved within the 22 families. One of the criteria required by the associative S_N_2 type mechanism is the creation of a pentavalent intermediate between the γ-phosphate (γ-PO_4_) of ATP and the substrate nucleophile. Accepted mechanisms leading to the creation of this pentavalent transition state and the activation of the γ-PO_4 _as a leaving group, all require protonation of the α-phosphate (α-PO_4_) or β-phosphate (β-PO_4_) of ATP, either directly or via a carrier side-chain (Figure 1). Similarly, protonation or hydrogen-bonding of N7 of the ATP adenyl group with the protein would lead to the C8-H becoming more acidic. This, in turn, requires an acidic proton to protonate N7 and an electron-donating group to stabilise the developing positive charge on C8. This process would allow for the C8-H to protonate the α-phosphoryl group, initiating a tautomerization sequence of reactions involving the acidic proton on the α-phosphoryl group being transferred, via a coordinated basic amino acid side-chain such as lysine, to the β-phosphoryl group. This results in the concomitant activation of the γ-phosphoryl group to nucleophilic attack by the target residue or substrate through a pentavalent phosphorus intermediate. A general mechanism for this process is outlined in Figure 1. This general mechanism was used as the template to define and characterise the family specific mechanisms associated with 21 of the kinase families within 10 of the fold groups where sufficient structural information is available. To this end, the

PDB (the database) was searched for structures representing kinases within each family, based on the EC numbering. All kinase crystal structures containing any AMP, ADP, ATP or corresponding analogues were then visually examined for structurally conserved amino acid side-chains that were associated with the nucleotide or analogue. The inter-atomic distances between the identified amino acid side-chain residues and the nucleotide analogue were also noted. Having identified the conserved mechanisms associated with crystallized representatives of each family, sequence alignments were carried out with other members of each specific family where structural information is not available. This allowed the determination of the extent to which the conserved residues identified in the individual families of kinases by this mechanistic analysis as being responsible for catalysis within all these families.

## Results

**Figure 2:**
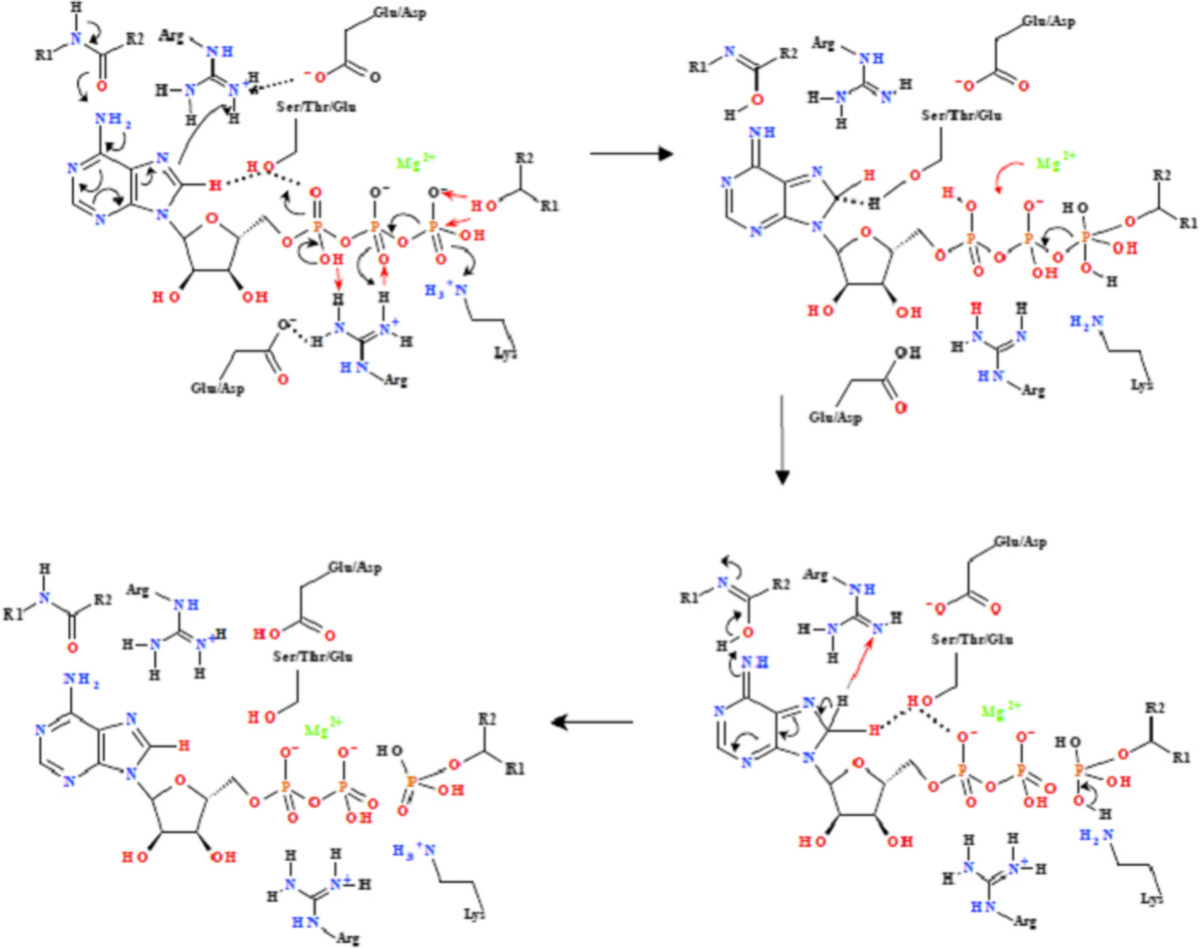
**Phosphoryl transfer mechanism found in the Group 2 kinases (Rossmann-like fold and phosphoenolpyruvte carboxykinase-like sequences)**. The initiation of phosphoryl transfer occurs via the coordination of the ATP C6-NH_2 _to a carbonyl arising from the protein backbone by the "push" mechanism resulting in the protonation of C8 via the coordination of a conserved Arg. This renders the C8-H more acidic, allowing for the protonation of the α-PO_4_, via a conserved Ser/Thr carrier. There is a concomitant transfer of an H^+ ^from the α-PO_4 _to β-PO_4 _via a conserved Arg, thereby facilitating the formation of the pentavalent intermediate between the γ-PO_4 _and the substrate nucleophile. There is a simultaneous ATP-mediated deprotonation of the substrate -OH, allowing for the nucleophilic attack by the substrate to create the pentavalent intermediate and allow phosphoryl transfer. A protonated Lys then transfers the proton to the γ-PO_4_, changing the Mg^2+ ^from being β-PO_4 _to γ-PO_4 _coordinated to being α-PO_4 _to β-PO_4 _coordinated. The H^+ ^originally arising from the C8 is then transferred back to C8, allowing the electron density of the adenyl moiety to return to the "ground-state" distribution.

**Figure 3:**
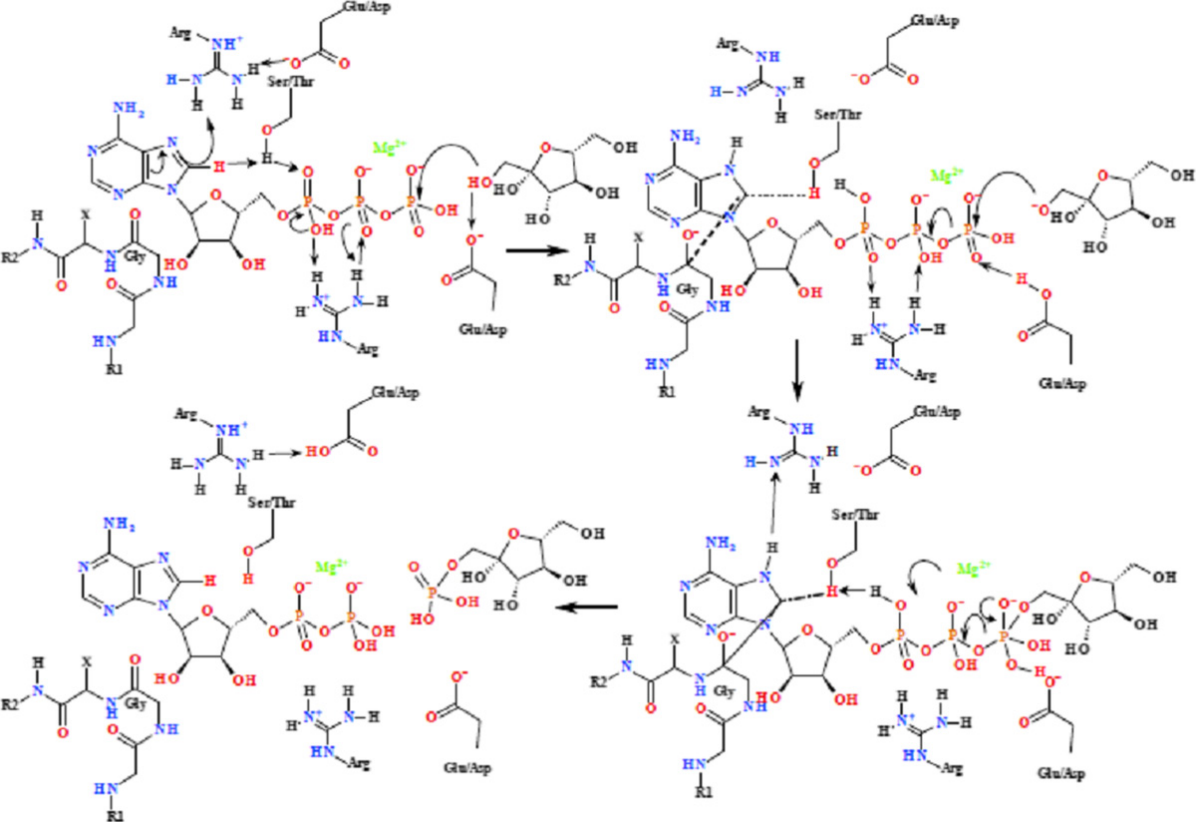
**Phosphoryl transfer mechanism found in the Group 4 kinases (hexokinase family with polyol substrate)**. This occurs via coordination of an arginine residue to the N7/C8 of the imidazole moiety mediating the change in C8 hybridization from sp2 to sp3 hybridized, and altering the protonation of N7 and C8. Protonation of the N7 occurs via a conserved the Arg residue with the NH_2 _being coordinated directly to N7, with an interatomic distance of 3.065 ± 0.823 Å. The Arg residue in Group 4 kinases is always stabilized by an associated Asp/Glu residue. The reaction occurs via a carbene mechanism with the carbene being stabilized via the interaction of a conserved backbone Gly carbonyl that is within bonding distance of C8, causing C8-H to become more acidic, allowing for the protonation of the α-PO_4_, via a conserved Ser/Thr. There is a concomitant transfer of an H^+ ^from the α-PO_4 _to the β-PO_4 _via a conserved Arg, thereby facilitating the formation of the pentavalent intermediate between the γ-PO_4 _and the substrate nucleophile. There is a concomitant Asp-mediated deprotonation of the substrate -OH, allowing for the nucleophilic attack by the substrate. This creates the pentavalent intermediate and allows phosphoryl transfer. The protonated Asp then transfers the proton to the γ-PO_4_, changing the coordination of the Mg^2+ ^from being β-PO_4 _to γ-PO_4 _coordinated to being α-PO_4 _to β-PO_4 _coordinated. The H^+ ^originally arising from the C8 is then transferred back to C8, allowing the electron density of the adenyl moiety to return to the "ground-state" distribution.

**Figure 4:**
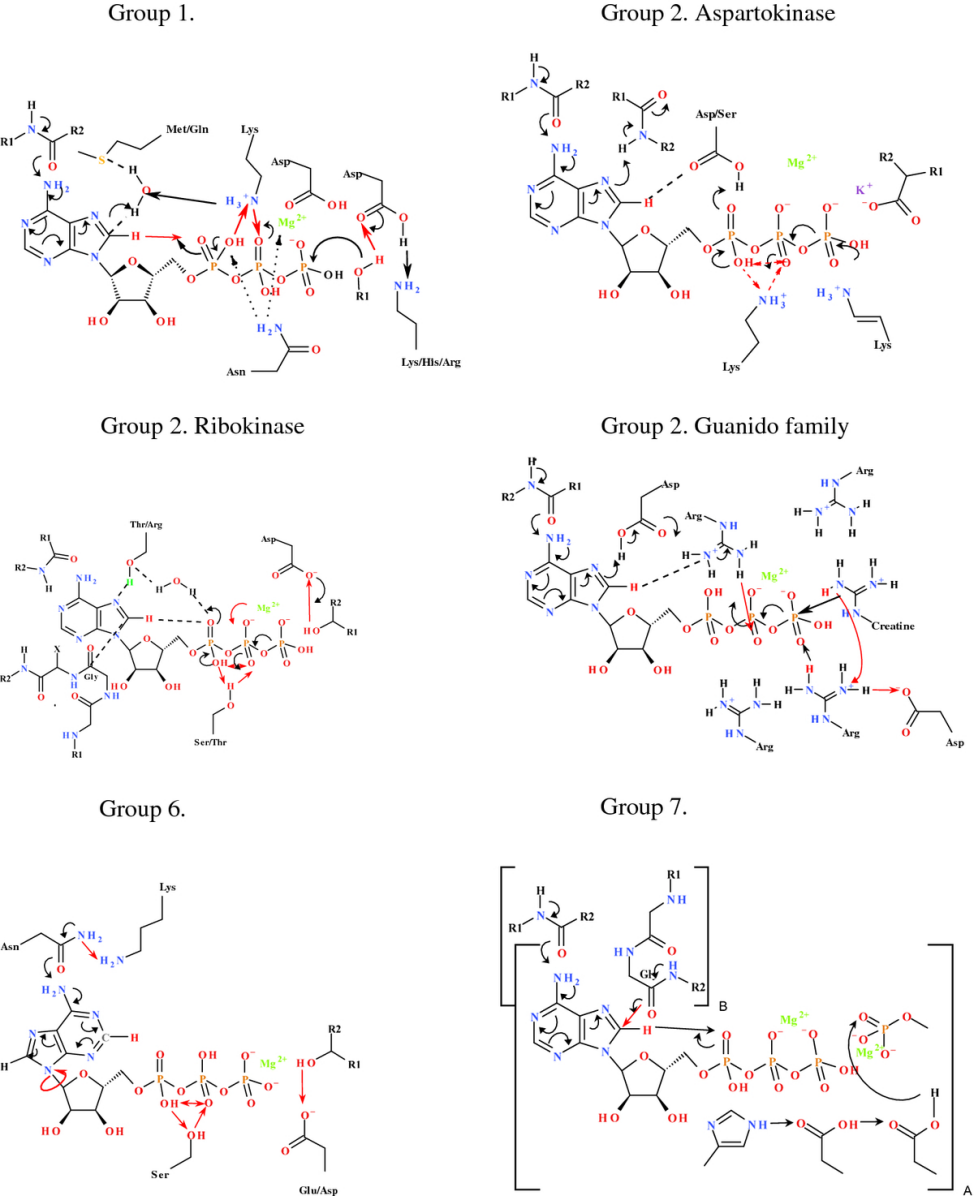
**Spatial arrangements of residues implicated in the phosphoryl transfer mechanism found in the Group 1, Group 2 (aspartokinase family), Group 2 (ribokinase family), Group 3 (Guanido family), Group 6 and Group 7 kinases**. The bracket indicates two distinct protein subunits.

In this classification of the kinase enzymes based on their conserved phosphoryl transfer mechanisms, the framework for the overall organisation remains the same as that of Cheek *et al *[2,3]. However, individual kinases may have been transferred from one family to another based on the conservation of the mechanism. It has been demonstrated that C8-H plays a critical role in the activity of a range of kinase and synthetase enzymes [4]. In all cases, the mechanism associated with the activation and release of the C8-H is based on the proximity of the C8-H to the α- or β-PO_4_, or to a proton carrier side-chain capable of transferring the proton from the C8-H to the α-PO_4_. In most cases, the C8-H is rendered more acidic by hydrogen bonding networks mediating either a "push" mechanism via the coordination to the C6-NH_2 _or a "pull" mechanism via the protonation of N7. In a significant number of cases a carbonyl group from the protein backbone is hydrogen bonded to the C6-NH_2 _of the nucleotide (Figure 1). In cases where this does not occur, there is a residue with a labile proton hydrogen-bonded with N7. The tautomeric interconversion that gives rise to planar peptide bonds allows for the strong hydrogen bonding interaction and/or proton transfer between the backbone carbonyl and the C6-NH_2 _[6,7]. Examples of the "push" and "pull" mechanisms for the kinase families are summarised Figures 2-3, while the detailed reaction mechanisms and the identified residue and interatomic distances outlined in Additional file 1: Table S3 and 4A. The Pfam superfamily and family classifications, as well as the sequence alignments showing the conserved residues within each family are shown in the supplementary information. Within each structurally conserved mechanism, the average interatomic distances between the conserved residues and nucleotide or nucleotide analogue are calculated and outlined in the tables. Figure 4 defines the spatial arrangement for the primary "structure" involved in the initiation of phosphoryl transfer for each of the remaining groups where structural information was available. The full mechanisms associated with these groups are shown in the supplementary information.

### "Push" mechanism

The Group 2 kinases consisting of the Rossmann-like kinases and the phosphoenolpyruvate carboxykinase families are used as the example of the "push" mechanism (Figure 2, (Additional file 1: Table S3). These enzymes fall within the P-loop containing nucleoside triphosphate hydrolase superfamily (Additional file 1: Table S4, Figures S8 & S9). The mechanism relies on a side-chain carrier associated with the transfer of the C8-H to the α-PO_4_, and then the activation of the nucleophile substrate occurs directly via the protonation of the γ-PO_4_. The protonation of the α-PO_4 _from C8-H in other mechanisms may occur directly (see below).

The initiation of phosphoryl transfer occurs via hydrogen bonding of the ATP C6-NH_2 _to a carbonyl group arising from the protein backbone, with the average hydrogen-bond distance from the C6-NH hydrogen to the backbone carbonyl oxygen being 2.101 ± 0.699 Å. This reaction relies on the "push" mechanism which, by the hydrogen bonding of C6-NH_2 _to the backbone carbonyl and the concomitant protonation of the developing anion at C8 by a conserved Arg residue acting as a Brønsted acid, produces a methylene group at C8 (Figure 2). On the opposite face of the adenyl moiety is a coordinated Thr/Ser which acts in the transfer of H^+ ^to the α-PO_4_. The change in the N7-C8 hybridization facilitates the protonation of the α-PO_4 _via the Thr/Ser, via the re-protonation of the Thr/Ser by C8-H. The effective protonation of the α-PO_4 _therefore occurs via the coordinated Ser/Thr as the C8-H is not within direct hydrogen bonding distance of the α-PO_4_, the interatomic distance being 4.322 ± 1.010 Å. The Thr/Ser to C8-H interatomic distance and the Thr/Ser to the α-PO_4 _inter-atomic distance were found to be 3.797 ± 1.697 Å and 3.200 ± 0.558 Å, respectively. The effective transfer of an H^+ ^between the α- and β-PO_4 _occurs via a co-ordinated conserved Arg residue for the Rossmann-like and phosphoenolpyruvate carboxykinase families. The co-ordinated Arg acts as a base by receiving a proton from the α-PO_4 _and also acts as an acid by protonating the β-PO_4_, with the conserved Arg to α-PO_4 _and β-PO_4 _interatomic distances being 2.953 ± 1.197 Å and 2.847 ± 1.143 Å, respectively. The substrate nucleophile is activated directly via the coordinated γ-PO_4 _and this probably plays a role in the translocation of the Mg^2+ ^from being β-PO_4_/γ-PO_4 _co-ordinated to being β-PO_4_/α-PO_4 _coordinated. A γ- PO_4_-coordinated Lys also ensures the creation of the pentavalent intermediate after the nucleophilic attack by the substrate with the lysine δ-NH_3 _to γ-PO_4 _inter-atomic distance being 2.073 ± 0.480 Å. The migration of the Mg^2+ ^to the β-PO_4_/α-PO_4 _co-ordination ensures that the proton arising from the C8 returns to the C8-H position. This occurs with the concomitant re-protonation of the co-ordinated Arg via the Ser/Thr/Asp and the subsequent return of the delocalization of the electrons of the adenine. Enzymes employing the "push" mechanism in the Group 2 in the P-loop containing nucleoside triphosphate hydrolase superfamily in which the C8-H dependent kinetic isotope effect has been demonstrated are shikimate kinase (4) and adenylate kinase (unpublished data).

### "Pull" mechanism

The Group 4 kinases rely on a distinctive mechanism for the activation of the C8-H based on the protonation of N7 initiating the creation of a carbene at C8. This is referred to as the "pull" mechanism. The Group 4 kinases fall within the Actin-like ATPase and Ribokinase-like superfamilies (Additional file 1: Table S8). The C8-H to α- or β-PO_4 _interatomic distance = 4.932 ± 0.876 Å (Figure 3, Additional file 1: Table S7). A significant difference between the Group 4 kinases and all other groups is the role of the adenyl ring associated Arg residue in the initiation of phosphoryl transfer. In this group there is no coordination of the ATP C6-NH_2 _to a carbonyl arising from the protein backbone. Instead the Arg NH_2 _is co-ordinated directly to N7, with the N7 inter-atomic distance being 3.155 ± 0.815 Å. It is proposed that it is this coordination within the Group 4 kinases that initiates the release of the C8-H with the concomitant initiation of phosphoryl transfer (Figure 3). Also making up the "pull mechanism", the Arg residue hydrogen bonded to the adenyl N7 in the Group 4 kinases is always stabilized by an associated Asp/Glu residue, assisting in the protonation of N7. Hydrogen bonding to C8-H by the hydroxyl moiety of a Ser/Thr, is associated with the transfer of the C8-H to the α- or β-PO_4_. The C8-H to α- or β-PO_4 _inter-atomic distance is 4.932 ± 0.876 Å, while the C8-H to Ser/Thr hydroxyl oxygen and Ser/Thr hydroxyl hydrogen to α-PO_4 _distances are 3.495 ± 0.446 Å and 2.672 ± 0.537 Å, respectively (Figure 3, Additional file 1: Table S7). The Arg and Ser residues are situated on opposite faces of the imidazole moiety of the adenyl group. The reaction occurs via a carbene mechanism with the carbene being stabilized via the interaction of a conserved backbone carbonyl that is within bonding distance of C8 at 2.781 ± 0.488 Å (Figure 3, Additional file 1: Table S7). The backbone carbonyl usually arises from a Gly residue. Once the hybridization of C8 from sp^2 ^to sp^3 ^has occurred, the protonation of the α-PO_4 _by the Ser and the concomitant release of the C8-H occurs, affording a stable carbene. The transfer of the proton between the α- and β-PO_4 _groups occurs via a coordinated Arg residue which is 2.756 ± 0.708 Å and 3.095 ± 0.908 Å, respectively, from the α- and β-PO_4 _groups. The activation of the nucleophile substrate hydroxyl group occurs via deprotonation by a coordinating Asp, rendering it more nucleophilic (polyol-OH hydrogen to the Asp oxygen average inter-atomic distance = 2.533 ± 0.583 Å). As discussed previously, once the proton translocation has occurred, a pentavalent phosphorus(V) intermediate is formed between the substrate and the γ-PO_4_, which is now in a position to act as a leaving group. The substrate is activated to nucleophilic attack on the γ-PO_4 _by deprotonation of a hydroxyl group by a hydrogen bonded Asp (polyol -OH to Asp inter-atomic distance is 2.533 ± 0.583 Å). The substrates phosphorylated by the hexokinase, FGGY, and ROK kinases are polyols while short-chain carboxylic acids are the substrates for the acetokinase family. The Group 4 kinases fall within the actin-like ATPase and ribokinase-like superfamilies (Additional file 1: Table S8). The C8-H dependent KIE has been demonstrated in hexokinase a member of the Actin-like ATPase superfamily of Group 4 [4].

### Group 1 kinases

The identified conserved amino acids in the Group 1 kinases, comprising the protein Ser/Thr-Tyr kinases, atypical protein kinases, lipid kinases and the ATP-grasp families are outlined in Supplementary Information (Additional file 1: Table S1). The identified residues associated with phosphoryl transfer distinguish the Group 1 kinases into 3 groups; (1) The protein kinase superfamily comprising families containing the protein kinase domain and protein tyrosine kinase domain, (2) the protein kinase superfamily comprising the choline kinase/ethanolamine kinase family, the phosphotransferase enzyme family and the amino glycoside/hydrourea antibiotic resistance kinase family, (3) and the lipid and ATP grasp kinases consisting of the phosphatidylinositol-4-phosphate 5-kinase and inositol polyphosphate kinase families (Additional file 1: Table S2, Figures S2, S3 & S4). The proposed reaction mechanism for the Protein kinase superfamily comprising families containing the protein kinase domain and protein tyrosine kinase domain relies on the "push" mechanism and the spatial arrangement for the initiation of the reaction is shown in Figure 4 (Additional file 1: Figure S1). The Protein kinase superfamily and protein kinase family are distinguished from all other superfamilies/families in that a conserved methionine residue is found within the active site in close proximity to C8. It is proposed that a water molecule is hydrogen bonded between the sulphur of the methionine and C8 of the adenyl ring creating the proton addition complex analogous to the Wheland intermediate implicated in electrophilic aromatic substitution. The C8-H is within H-bonding distance of the α-PO_4 _(3.050 ± 0.271 Å) and the H^+ ^is transferred directly to the α-PO_4_, allowing for the effective transfer of the H^+ ^to the β-PO_4 _via a coordinating lysine or arginine residue. Other than the ATP-grasp kinases, the nucleophile in all cases is an -OH, which is deprotonated by a coordinating Asp and Asn/Lys/His/Arg combination, rendering it more nucleophilic. The rearrangement of the coordination of the Mg^2+ ^probably contributes to the creation of the pentavalent intermediate and as a result of the protonation of the β-PO_4 _by the coordinating Lys, ensures that the γ-PO_4 _is a better leaving group. As the γ-PO_4_-phosphorylated nucleophile leaves the pentavalent transition state, the H^+ ^coordinated to the Asp and Lys/His/Arg, which arises from the nucleophile, then "returns" to the γ-PO_4_. This allows for the migration of the Mg^2+ ^to be β-PO_4_/α-PO_4 _coordinated and the return of the H^+ ^at the β-PO_4 _to the α-PO_4_. Finally, C8 is re-protonated and subsequently the delocalization of the electrons of the adenine returns to its most stable tautomer. The detailed reaction mechanism is outlined in the Supplementary Information (Additional file 1: Table S1).

A major variation in the conserved catalytic mechanism within this group, that arises between the protein Ser/Thr-Tyr kinases and atypical protein kinases on one hand, and the lipid and the ATP-grasp kinases on the other, is the difference lies in the activation of the substrate for nucleophilic attack on the γ-PO_4_. Generally the protein Ser/Thr-Tyr kinases and atypical protein kinases function via a conserved Asp/Asn residue that allows for the deprotonation of the substrate -OH while the lipid kinases appear to function using a conserved Asp/Lys residue. There are however examples of conserved amino acid functionality e.g. Asp to Glu. Furthermore, substrate activation by the ATP-grasp kinases, which utilize acid substrates *e.g*. the acetate, butyrate and propionate, appears to make use of the mechanism found in the Group 4 kinases in conjunction with a number of His residues (see Group 4, Additional file 1: Table S7).

The differentiation of the Group 1 kinases can be extended to 3 distinct "sub-groups" based on the sequence alignments of the conserved functionality identified by the mechanism by which phophorylation is initiated (Additional file 1: Figures S2, S3 & S4). In each case, the residues involved in the initiation of phosphoryl transfer are conserved within the sub-group. Other than these conserved residues, there is very little sequence homology within the families. Based on the Pfam family classification the protein Ser/Thr-Tyr kinase family, α-PO_4 _to β-PO_4 _the proton transfer is mediated via a coordinated lysine while in the choline kinase family the α-PO_4 _to β-PO_4 _transfer is mediated via a conserved Arg residue (Additional file 1: Table S1, Figure S2 & S3). However, two structures which fall within the phosphotransferase family also utilise Lys for the α-PO_4 _to β-PO_4 _proton transfer. These sequences however, are not similar to the protein Tyr kinase sequences (Additional file 1: Figure S2 & S3). The conserved functionality within the lipid kinases depends on the complexities/order of domain the assembly. Taking the order of the functional domain assembly into account, the identified functional residues within the families of the lipid kinases remain conserved (Additional file 1: Figure S4).

### Group 2 kinases

The Group 2 kinases consist of the Rossmann-like kinases, phosphoenolpyruvate carboxykinase, phosphoglycerate kinase, aspartokinase-like kinases, phosphofructokinase-like kinases, ribokinase-like kinases, thiamine pyrophosphokinase and glycerate kinase.

The mechanism associated with the Rossmann-like kinases and phosphoenolpyruvate carboxykinase has been outlined in the definition of the "push" mechanism. Structurally and mechanistically, the pantothenate and deoxyguanosine kinases differ from the other kinases located in the Rossmann-like and phosphoenolpyruvate carboxykinase families in that only one Arg is implicated in the phosphoryl transfer mechanism and the Arg is located on the opposite face of the ribose sugar (the "D-face") (Additional file 1: Table S3). This Arg plays a role in the protonation of the C8. However, this residue is also in close enough proximity to the α-PO_4 _and the β-PO_4_, to facilitate proton transfer. A primary sequence alignment of the structures within the Rossmann-like kinases (members of the P-loop containing nucleoside triphosphate hydrolase PFam superfamily) shows two distinct sub-families, grouped based on the conserved residues associated with reaction mechanisms (Additional file 1: Table S4, Figure S8 & S9). The conserved amino acid residues identified in Additional file 1: Table S3 are highlighted in Additional file 1: Figures S8 & S9. The two distinct modes of functionality identified are not necessarily conserved within a family (Additional file 1: Table S4 & Figures S8 & S9). The shikimate kinase, uridine kinase and adenylate kinase families all contain two conserved Arg residues in the active site linked to the proton translocation (Mechanism 2A) while the second family contains only one conserved Arg residue (Mechanism 2B).

The initiation of phosphoryl transfer with the aspartokinase family within Group 2 also relies on the "push" mechanism however, the spatial arrangement of the residues responsible for the reaction differ from the P-loop containing nucleoside triphosphate hydrolase superfamily (Figure 4, Additional file 1: Figure S6). Mechanistically, the aspartokinase family diverges from the Rossmann-like kinases and phosphoenolpyruvate carboxykinase in that there is no Arg associated with the adenyl ring to facilitate the protonation of C8. Within the aspartokinase family protonation of C8 is achieved via the coordination of a backbone amide. However, common to this family is the transfer of the C8-H to an intermediate Asp/Ser which leads to the protonation of the α-PO_4_, as well as the presence of a co-ordinating Lys which is responsible for the inter-α-PO_4_/β-PO_4 _transfer (Figure 4; Additional file 1: Table S4, Figure S6, Figure S10). Within the aspartokinase family, the enzyme aspartokinase has an additional Arg residue between the Asp which facilitates the deprotonation of C8 and the protonation α-PO_4_. A primary sequence alignment of the proteins making up the aspartokinase family identified all the functional residues in a region of relatively high sequence homology (Additional file 1: Table S4 & Figure S10).

The Group 2 ribokinase-like superfamily use the distinctive "pull" mechanism based on the protonation of N7 initiating the formation of a carbene at C8 as occurs in the Group 4 kinases. As occurs in the Group 4 kinases the carbene is stabilized via a reaction with a coordinated backbone carbonyl arising from a conserved Gly residue. The Group 2 ribokinase-like superfamily requires a conserved Thr/Arg for the protonation of N7 and subsequent protonation of the α-PO_4 _from C8 via a conserved H_2_O. The proton transfer between the α- and β-PO_4 _is also mediated via a conserved Thr/Lys (Figure 4, Additional file 1: Table S4, Figure S7, Figure S11). A distinguishing feature of this group is the interaction of a peptide bond with the adenyl group, spanning C6-NH_2 _and N1, possibly stabilising the adenyl ring. The protonation of C8 occurs via the coordination of a conserved Thr/Lys. Within the ribokinase-, PFK- and hexokinase-like superfamilies the "pull" mechanism is achieved by similar spatial arrangements achieved by 3 distinct groups as is evident from their sequence alignments (Additional file 1: Figures S11). Also included in the Group 2 is phosphoglycerate kinase family [2,3]. This family, however, carries out the reverse reaction and therefore does not require the C8-H to facilitate phosphoryl transfer. Therefore, within the structure of phosphoglycerate kinase, no residues responsible for facilitating the formation of the pentavalent intermediate between the substrate and ATP are found. Within the phosphomethyl pyrimidine kinase and ADP-specific phosphofructokinase/glucokinase families of the ribokinase superfamily the reactions are either ADP dependent or transfer pyrophosphate and therefore do not have a residue responsible for proton transfer between the α- and β-phosphate.

### Group 3 kinases

The reaction mechanism identified for the Group 3 kinases is limited to the structural information obtained from the guanido family comprising creatine and arginine kinase (Figure 4, Additional file 1: Table S5, Figure S14). There is, however, significant sequence and structural homology between these enzymes (Additional file 1: Table S5 & Figure S15). As with many kinase groups discussed, this reaction is initiated by the "push" mechanism via coordination of a carbonyl group arising from the protein backbone, to the ATP C6-NH_2 _(spatial arrangement outlined in Figure 4). The acidification of the C8-H allows for the protonation of the coordinated Arg, allowing proton shuffling resulting in the required tautomeric forms of the α- and β-PO_4 _residues. This results in the protonation of the β-PO_4 _via the electron delocalization across the Arg guanidium group and proton transfer from the Arg NεH to β-PO_4_. The creatine/arginine substrate guanidium group is activated by deprotonation via a coordinating Arg residue allowing for the formation of the pentavalent intermediate between the substrate and the γ-PO_4 _as a leaving group (Figure 4, Additional file 1: Table S5). The inductive effect of the Mg^2+ ^renders the renders the γ-PO_4 _residue more electrophilic, promoting addition of the creatine nucleophile. The protonation state is restored by the interaction of proximal Asp and Arg residues, and the proton translocation is reversed after phosphoryl transfer. The conserved functionality within Group 3 is as outlined (Additional file 1: Figure S15). The Arg responsible for the substrate deprotonation is stabilized via a coordinated Asp residue which acts to deprotonate the Arg prior to the deprotonation of the substrate.

### Group 4 kinases

The Group 4 kinases are outlined as the definition of the "pull" mechanism. Within the FGGY family of the Actin-like superfamily, two distinct sub-families may be defined in terms of the residues required for functionality (Additional file 1: Table S8 & Figures S17 & S18). Within the ROK family of the Actin-like ATPase superfamily, the amino acid residues involved in phosphoryl transfer cannot be identified as no crystal structure containing a nucleotide exists (Additional file 1: Table S8 & Figure S4). The functionality is well conserved within the acetokinase family (Additional file 1: Table S8 & Figure S20). The residues involved in phosphoryl transfer within this acetokinase family differ from those in the hexokinase, FGGY, and ROK kinases. This mechanism involves a number of conserved His residues and may not involve the C8-H. However, protonation of the phosphate backbone of ATP is still required for phosphoryl transfer. The proton may originate from the substrate and be transferred along to the α-PO_4 _via a cascade of conserved His residues (Additional file 1: Figure S20). The hexokinase family appears to have a similar mechanism as occurs within all the Group 4 kinases; however, only a single structure containing a nucleotide ligand exists (Additional file 1: Table S8 & Figure S21).

### Group 5 kinases

Group 5 (TIM β/α-barrel kinases) contains pyruvate kinase as the example which, as with phosphoglycerate kinase in Group 2, carries out the reverse reaction synthesizing ATP. Both examples contain no amino acid side-chains associated with the acidification of C8-H coordinated to the adenyl group of the nucleotide. Side-chains deemed to be necessary for the acidification of C8-H are the coordinating backbone carbonyl group capable of interacting with the N6-NH_2 _and/or side-chains coordinated with either N7 or C8.

### Group 6 kinases

The Group 6 kinases (Figure 4, Additional file 1: Table S9) comprises three known structures that have a conserved active site in which the adenyl ring is in a *syn *conformation relative to the ribose, allowing for the coordination of the C3-H to the α-PO_4_. The critical question that arises as to the putative role C3-H may play in inducing phosphoryl transfer due to its proximity to the α-PO_4 _and hence, by analogy to the situation in the case of the C8-H in other group, would be purely speculative. No KIE evidence was obtained in steady-state enzyme reactions to suggest that the C3-H of adenine plays a similar role in this group of kinases as the C8-H does within the other groups [4]. It is however conceivable that the ATP only binds in the *syn *conformation and is induced to rotate on its glycosidic bond to the *anti *conformation as part of the overall mechanism to binding specificity. Using the same theme of using conserved amino acid residues within a group of kinases to suggest mechanistic implications, it is proposed that this reaction is initiated by the coordination of the conserved Asn γC-carbonyl group to the ATP C6-NH_2 _(Additional file 1: Figure S22). The coordination of a conserved lysine with the concomitant delocalization of the electrons of the adenyl group results in the re-hybridization of C8 from sp^2 ^to sp^3^, that, along with the change of the conformation of the adenyl group from the *syn *to the *anti*-conformation there allows for the protonation of C8 by the lysine residue forming the carbene with the concomitant protonation of the α-PO_4 _from C8. The proton translocation from the α-PO_4 _creating the pentavalent intermediate then occurs. The lysine residue serves two roles, one being the deprotonation of the Asn amide in the activation of the amide to facilitate the deprotonation of the ATP C6-NH_2_, and the second role is in the protonation of C8 in the stabilization of the carbene. A proton required for the creation of the pentatvalent intermediate also originates from the substrate via the Glu/Asp deprotonation of the substrate. The members of Group 6 are within the Ribosomal protein S5 s-like superfamily comprising the GHMP family (Additional file 1: Table S10 & Figure S23).

### Group 7 kinases

The Group 7 kinases comprise thiamine monophosphate kinase and selenide water dikinase. The thiamine monophosphate kinase (EC 2.7.4.16) is from a prokaryotic system and uses ATP to phosphorylate thiamine monophosphate to thiamine pyrophosphate, while producing ADP. This thiamine monophosphate kinase differs from the thiamine pyrophosphate kinase (Group 2) and 2-amino-4-hydroxy-6-hydroxymethyldihydropteridine pyrophosphokinase (Group 3) in that the latter two are pyrophosphotransferases transferring a pyrophosphate group from a nucleoside triphosphate, such as ATP to the hydroxyl of thiamine. All enzymes in Group 7 are homo-dimeric, with the active site comprising amino acid residues from both subunits (Figure 4, Additional file 1: Table S11, Figure S24). It is only on the formation of the dimer that the reaction can occur, since the carbonyl group coordinating ATP C6-NH_2 _in the "pull" mechanism implied here, as well as a conserved backbone carbonyl which stabilizes the carbene formed at C8, both arise from residues in one subunit, with the remainder of the coordinating residues arising from the second subunit. The initiation of phosphoryl transfer therefore only occurs on dimerization, enabling adequate association of the various residues with the ATP molecule. The reaction mediated by the thiamine monophosphate kinase is initiated once proton transfer has occurred from C8-H to the α-PO_4_. This is followed by protonation of the substrate via a cascade involving a conserved His residue and a series of Asp residues (Figure 4; Additional file 1: Figure AF 10). The interatomic distances are: α-PO_4 _to His = 2.748 ± 1.25 Å, His to Asp = 4.020 ± 1.411 Å and Asp to Asp = 3.923 ± 1.357 Å. The protonation of the substrate allows the pentavalent intermediate to be formed, and the coordination of the Mg^2+ ^to migrate from being the β-PO_4_/γ-PO_4 _coordinated to being α-PO_4_/β-PO_4 _coordinated, with the concomitant re-protonation of C8.

### Groups 8

There is only one member in each of Group 8, Group 9, Group 10 and Group 11 kinases. Group 8 consists of riboflavin kinase from *Schizosaccharomyces pombe *and *Homo sapiens *(flavokinase family) (Additional file 1: Table S12). The postulated phosphoryl transfer mechanism is similar to all other groups utilizing the "push"mechanism (Additional file 1: Table S12, Figure S25 & Figure S26). The mechanism occurs via coordination of the adenyl C6-NH_2 _and protonation of C8 via a coordinated Lys changing C8 from sp2 to sp3 hybridization, and alters the protonation of C8-H. The C8-H becomes more acidic, allowing for the protonation of the β-PO_4_, via a conserved Asp to Arg proton transfer. The H^+ ^transfer is directly to the β-PO_4_, facilitating the formation of the pentavalent intermediate between the γ-PO_4 _and the substrate nucleophile. There is a simultaneous Glu-mediated deprotonation of the substrate-OH that allows for the nucleophilic attack by the substrate, creating the pentavalent intermediate and allowing phosphoryl transfer. The protonated Glu then transfers the proton to the γ-PO_4 _changing the coordination of the Mg^2+ ^from being β-PO_4 _to γ-PO_4 _coordinated to being α-PO_4 _to β-PO_4 _coordinated. The H^+ ^originally arising from the C8 is then transferred back to C8, allowing the electron density of the adenyl moiety to return to the "ground-state" distribution.

### Groups 9 to 12 kinases

Group 9 contains dihydroxacetone kinase from *Citrobacter freundii*, Group 10 contains glycerate kinase from *Neisseria meningitides *and Group 11 polyphosphate kinase from *Escherichia coli *(Additional file 1: Table S13). Not enough structural data was found for Groups 9, 10 and 12 to allow for the elucidation of the reaction mechanisms. The postulated phosphoryl transfer mechanism found in the Group 11 kinases which utilizes the "push"mechanism but also undergoes an autophosphorylation of a histidine residue (polyphosphate family) (Additional file 1: Table S14, Figure S27 & Figure S28) [8,9]. The mechanism occurs via coordination of the adenyl C6-NH_2 _from the protein backbone to the ATP C6-NH_2 _and an Asn carbonyl which acts to stabilize the carbene formed at C8. The C8-H becomes more acidic, allowing for the protonation of the α-PO_4_, via a conserved Arg proton transfer. A second conserved Arg transfers the proton from the α-PO_4 _to the γ-PO_4 _facilitating the formation of the pentavalent intermediate to allow for phosphoryl transfer. There is a simultaneous deprotonation of a conserved His allowing for the nucleophilic attack of the His for the γ-PO_4_. There is also a putative deprotonation of the substrate phosphate by a second His allowing for the concomitant formation of the phosphate dimer (polymer). The H^+ ^originally arising from the C8 is then transferred back to C8, allowing the electron density of the adenyl moiety to return to the "ground-state" distribution.

## Discussion

The role of C8 in electrophilic substitution reactions is not unprecedented in nucleotides under basic conditions, where the lone pairs on N3 and N7 are available to donate electron density through the conjugated system to facilitate the electrophilic attack at C8 [10-12]. A number of conserved mechanisms have been identified whereby the C8H of ATP plays a direct role in the initiation of phosphoryl transfer in kinase reactions. A required aspect of this is the locking of the adenyl moiety into position by interactions with C6-NH_2_, impacting on the delocalization of electrons in the adenyl moiety as a result. Within the group classification as outlined by Cheek *et al*, this role of the C6-NH_2 _is found in all groups other than Group 4. In these groups resonance interactions giving the backbone peptide bond the planar nature with double character and the concomitant impact on the C6-NH_2_, facilitates this process via the charge distribution across the peptide bond and the charge to charge interaction with the N6-NH_2_. In Group 4 the interaction of a conserved Arg residue interacting with the N7/C8 facilitates the change in hybridization of C8 back to sp^2 ^with the concomitant release of the C8H initiating phosphoryl transfer.

It is from this point that phosphoryl transfer is initiated via a number of family-specific proton transfer cascades. Two mechanisms therefore arise by which the adenyl moiety of ATP plays a direct role in the initiation of phosphoryl transfer; a "push" mechanism arising from the coordination of a conserved backbone moiety coordinated to the C6-NH_2 _and a "pull" mechanism arising from the protonation of N7 to facilitate the creation of a carbene at C8. Each of these mechanisms would allow for the regulation of the rate of formation of the pentavalent intermediate between the ATP and the substrate nucleophile. Phosphoryl transfer within kinases is therefore a specific event mediated and regulated via the coordination of the adenyl moiety of ATP. Within families the alignment of sequences generally gave very low sequence identities. However, the residues associated with phosphoryl transfer are always conserved and, more importantly, the residues identified for their role in the general acid and base catalysis associated with the initiation of the reaction and the creation of the pentavalent transition are also always conserved within families. The secondary structure elements within families are conserved. The evolution of these kinase catalytic mechanisms appears to be convergent evolution as within groups and families both prokaryotic and eukaryotic organisms are found. This is especially born out in the Protein Ser/Thr-Tyr kinases and Atypical protein kinases as the enzymes arise from a large array of organisms utilizing varied substrates. In all cases phosphorylation is of the substrate hydroxyl group. The evolution of these kinase catalytic mechanisms appears to be based on controlling the rates two simultaneous mechanisms that occur during substrate phosphorylation. The first being the rate of deprotonation of the substrate nucleophile and the second being the rate of protonation fteγ-PO_4_of the ATP to allow for the formation of the pentavalent intermediate. In thekinase enzymes therefore, within the catalytic site of the enzyme, the rate of reaction is dependent on two factors, the induced *pK*_a _of the substrate and rate of induction of the C8H to become labile allowing for the effective transfer of a proton to the γ-PO_4 _of the ATP to allow for the formation of the pentavalent intermediate. These mechanisms have therefore evolved independently but convergently to achieve specific rates of reactions. These reaction rates probably allow for the overall rate of reaction of each enzyme to be regulated within a range. The range is dictated by the mechanistic classification. Each mechanistic group has a different number of bonds through which the tautomerization and proton translocations need to occur.

A significant number of kinase enzymes also undergo large molecular dynamic motions during the course of each reaction. The molecular dynamics associated with the binding mechanisms and their role in the orientation of specific amino residues to ensure the release of the C8-H to allow for phosphoryl transfer is borne out in a number of systems. In the remodeling of the σ54* by the bacterial enhancer protein belonging to the NtrC subclass of the AAA + ATPases, the ATP binding site resides at the interface between two subunits [13-15]. Comparing the ATP-bound structure with the ADP-bound structure demonstrated large-scale conformational changes concomitant with ATP binding. Movement induced in the walker subunit in strand β7 and Linker 1 is associated with the partial unwinding and translocation of helix H8 and with the upward and outward role of the L1 loop and its σ54-binding GAFTA motif. Concomitant to the major conformational changes which occur in the walker subunit associated with σ54-binding is a major re-alignment of the residues associated with the "push" mechanism. The "push" mechanism utilized is similar to that used by the Group 3 kinases. A comparison of the inter-atomic distances in the ATP- and ADP-bound structures in the residues associated with the "push" mechanism demonstrated significant reduction in the inter-atomic distances (PDB: 3M0E and 1NY6[14,15]. The inter-atomic distances for the ADP- and ATP-bound structures between the ATP/ADP C6-NH_2 _to backbone carbonyl (Val140) inducing the tautomeric change in the adenyl ring, are 2.710 and 2.053 Å, respectively. The reaction occurs via a carbene mechanism with the carbene being stabilized via the interaction of a conserved backbone carbonyl (Gly170) that is within bonding distance of C8, causing C8-H to become more acidic, allowing for the direct protonation of the α-PO_4_, with the inter-atomic distances for the ADP- and ATP-bound structures being 4.688 and 3.896 Å, respectively (See Group 7 kinases). The proton transfer creating the pentavalent intermediate occurs via Arg357 with the inter-atomic distances for the ADP- and ATP-bound structures being .5.807 and 2.221 Å to the α-PO_4_, 8.673 and 4.046 Å to the β-PO_4 _and 2.254 to the γ-PO_4_, respectively. The conformational changes induced in the structure of the ATP are caused by the interactions of Arg299 on the Rfinger subunit interacting with the ATP in the active site of the Walker subunit, the inter-atomic distances for the ADP- and ATP-bound structures being between Arg299 and the β-PO_4 _being 11.136 and 4.056 Å, respectively.

Cyclin dependent kinase-2 (CDK2) is a Ser/Thr protein kinase belonging to Group 1 which only shows catalytic activity when the subunit is bound by the allosteric Cyclin protein and the catalytic domain has been phosphorylated on Thr160 located within the kinase "activation loop" motif [16,17]. This enzyme also undergoes significant catalytic site closing motion on binding the peptide substrate and a second Mg^2+ ^ion [18]. The high-resolution refinement of CDK2 reveals 12 ordered water molecules in the ATP binding pocket of the apoenzyme and five ordered waters in that of the ATP complex. Despite a large number of hydrogen bonds between ATP-phosphates and CDK2, binding studies of cyclic AMP-dependent protein kinase with ATP analogues show that the triphosphate moiety contributes little and the adenine ring is most important for binding affinity [19,20]. Critical to the Group 1 mechanism is the ordered water molecule that resides between the Glu131 αC-C = O and C8-H. The extent of the molecular dynamics associated with the binding of the substrate and the ATP is indicated in the change in the inter-atomic distances between the structure only containing an γ-S ATP and 1 Mn^2+ ^and a structure containing ADP, MgF_3_, 2 Mg^2+^and the peptide substrate (Additional file 1: Table S1).

Structural evidence for the formation of the pentavalent intermediate in a bimolecular system as found in a kinase reaction has been shown in the phosphorylation of arginine by arginine kinase [21]. Using the transition state analogue complex, the nitrate mimics the planar γ-phosphoryl during associative in-line transfer between ATP and arginine and it is possible to measure how precisely the reactants are pre-aligned by the enzyme. The alignments were found to be exquisite. Substrates are positioned not only in close proximity but with orbitals aligned close to optimally. Consistent with in-line transfer, the donor and acceptor atoms (ADP β-O, and guanidinyl N_η_) are positioned to form bonds within 0.2° orthogonal to the phosphoryl (nitrate) plane. With respect to the guanidinyl N_η_, the optimal direction for nucleophilic attack by its lone pair is at an angle of approximately 110° to the N_η_-C_γ _bond in a plane perpendicular to the guanidinium plane. The nitrate nitrogen, mimicking the P_γ_, is at 115°, close to ideal.

## Conclusions

The kinase enzymes have been classified into 25 families of homologous proteins, with the families assembled into 12 fold-groups based on the similarity of their structural folds [2]. However, this classification relays little, if any, information on the catalytic and regulatory mechanisms employed in nucleotide binding and phosphoryl transfer. Within a single group, both prokaryotic and eukaryotic organisms are represented with kinase isoenzymes that appear to be kinetically and functionally distinct based on the rate of phosphoryl transfer and the regulation thereof. Clearly the regulation of enzyme activity in kinases is complex, which manifests in the apparent *K*_M _of the kinases ranging from less than 0.4 μM to in excess of 1000 μM for ATP (Carna Biosciences, Inc., Kinase Profiling Book:http://www.carnabio.com, [22]). It is conceivable that the various conserved "push" and "pull" mechanisms associated with the release of C8-H, the proton transfer cascades and the resultant/concomitant creation of the pentavalent transition state are the mechanism by which the kinase enzymes achieve this 2500 fold variation in the *K*_M_. Permutations and combinations of the various mechanisms identified are therefore employed by the kinase enzymes to achieve the variation in enzyme kinetics of this diverse group of proteins effectively carrying out the same phosphorylation reaction. The initiation and regulation of the rate of this reaction is achieved by the manner in which the C8-H is induced to be labile and the mechanism by which the resultant proton transfer cascade is achieved in the ultimate creation of the pentavalent transition state between the γ-phosphate of the ATP and the substrate nucleophile. Within the active site of the enzyme the rate of reaction is therefore governed firstly by the various mechanisms by which the C8-H is induced to be labile and secondly, by the mechanisms by which the resultant effective translocation of a proton from C8-H to the pentavalent transition state between the γ-phosphate of the ATP and the substrate nucleophile occur. In kinase enzyme steady state enzyme kinetics the overall perceived reaction rate therefore comprises the contribution of this catalytic effect as well as the contribution of the first order concentration effect of the ATP. The rate of reaction in kinases is therefore dictated by the hydrogen bonding network that is set up between the amino acids on the surface of the protein binding site, the ATP molecule and any substrate molecule(s) that may be present. The key result of this interaction network is the change in the distribution of electron density in the heteroaromatic ring by two possible means, each facilitating the weakening of the C8-H bond and translocation of the proton to the triphosphate chain. This, in turn, ensures the formation of the pentavalent transition state through the hydrogen-bond mediated activation of the gamma phosphate group and subsequent nucleophilic attack thereof by the substrate. A number of conserved mechanisms have been identified by which the translocation of the C8 proton and subsequent activation of the gamma phosphate may occur. The contribution of these steps, both the electron redistribution in the heterocycle and subsequent activation of the triphosphate chain, to the overall reaction rate depends on the free energy contribution of each hydrogen bond used by the specific conserved mechanisms in question to the activation energy of the pentavalent transition state. The free energy contributions across the entire system, both small molecules and the protein, can probably be considered as a linear combination of the wave functions describing the electron distribution in the heterocycle, as well as the subsequent hydrogen bonding events.

## Methods

### Structural bioinformatics

Discovery Studio^® ^(Accelrys Inc) was used for all molecular modelling protocols. Structures were identified within the wwPDB using the EC numbers in the families and groups outlined by Cheek *et al *[2,3]. Only structures containing AMP, ADP, ATP or their non-hydrolysable analogues were used. Interatomic and hydrogen bonding distances were measured between the nucleotide and the coordinating amino acid. where required the pyrimidine/β-D-sugar torsion angle of the nucleotide, Thr/Ser -OH torsion angle and the torsion angles within the coordinating arginine residues were rotated to optimize the interatomic distances obtained. Where required an energy-minimization was then carried out on the structure and a "bump" check was carried out on the structure to ensure that no inappropriate interactions occurred. Energy minimizations were carried out using the standard Accelrys Discovery Studio protocols using the 'Smart Minimizer' algorithm over a maximum of 10000 steps to a RMS equal to 0.1 and an energy change of 0.0. All the structural informatics accession numbers and the associated references are included in the supplementary information. All structural informatics references to be included in the additional information.

### Sequence alignments and structural analysis

The protein sequence alignments were carried using the Accelrys Discovery Studio (Client 2.5) sequence alignment software, using the dynamic alignment with a gap penalty of 10, a multiple gap extension penalty of 0.05 and the BLOSUM scoring matrix. PFam searches were carried out to identify the kinase superfamilies and families. Sequence alignments were carried out within the superfamlies/families to identify the extent to which the amino acids associated with catalysis are conserved. The conserved secondary structure elements within families were also used to facilitate the sequence alignments. All sequence alignments are showing the conserved residues and the secondary structure elements are included in the supplementary information.

## Competing interests

The authors declare that they have no competing interests.

## Authors' contributions

CPK formulated the concepts, carried out protein structural analysis, sequence alignments, defined reaction mechanisms and drafted and edited the manuscript, RLR carried out sequence alignments and edited the manuscript, CWvdW defined reaction mechanisms and edited the manuscript and CJP defined reaction mechanisms and edited the manuscript. All authors read and approved the final manuscript.

## Supplementary Material

Additional file 1 **Supplementary information**. Click here for file
