# Supplementary material for: Conserved phosphoryl transfer mechanisms within kinase families and the role of the C8 proton of ATP in the activation of phosphoryl transfer
Source: BMC Res Notes. 2012 Mar 8;5:131. doi: 10.1186/1756-0500-5-131 (PMC3327638; doi:10.1186/1756-0500-5-131)
Supplement: Additional file 1 — Supplementary information. [file 1756-0500-5-131-S1.DOC]

**ADDITIONAL INFORMATION**

**Table AF 1A.** Kinases representing **Group 1** and the identified conserved amino acid residues associated with the catalysis of phosphoryl transfer and the measured interatomic distances are shown. The PDB or uniprot accession numbers are indicated. Conserved residues replaced by conserved functionality are indicated by 3-letter code.

| **Kinase** | **Met-S or Gln-C=O to Aden-NH_2_^1^** | **C8-H to α-PO_4_** | **Lys-NH_3_ to α-PO_4_^2^** | **Lys-NH_3_ to β-PO_4_^2^** | **Asp-COOH to β-PO_4_^3^** | **Asp-COOH to Mg^2+,3^** | **Substrate H^+^ removal^4,5,6^** |
| --- | --- | --- | --- | --- | --- | --- | --- |
| **Protein Ser/Thr-Tyr kinases / Atypical protein kinases** | | | | | | | |
| Choline kinase (Choline-OH) 3G15 (*Homo sapiens*) | Gln207Cα  2.004 | 2.809 | Arg146 1.956 | Arg146 3.798 | Asp330 2.942 | Asp330 2.242 | Asp306 Asn311 |
| Protein Ser/Thr kinase (Protein Ser/Thr-OH) 2BZK (*Homo sapiens*) | Pro123 Cα  3.322 | 4.226 | Lys67 1.830 | Lys67 3.025 | Asp186 3.104 | No Mg^2+^ | Asp167 Asn172 |
| Phosphorylase kinase (Glycogen phosphorylase-Ser-OH) 2PHK (Rabbit) | Asp104 1.934 Met106 5.412 | 3.654 | Lys48 2.489 | Lys48 3.947 | Asp167 3.204 | Mn^2+^ coordinated | Asp149 Asn154 Arg7 |
| Homoserine kinase  P00547 (*Escherichia coli*) | NS |  |  |  |  |  |  |
| 1 Phosphotidylinositol 4-phosphate 5-kinase 2GK9 (*Homo sapiens*) | No ADP |  | Lys167 | Lys167 | Asp227 | Asp227 | Asp227  Lys171 |
| Streptomycin 6-kinase P08077 (*Streptomyces griseus*) | NS |  | Arg64 | Arg64 | Asp219 | Asp219 | His199 Asp201 |
| Ethanolamine kinase^7^ Q9HBU6 (*Homo sapiens*) | NS |  | Arg167 | Arg167 | Asp329 | Asp329 | Asp310 Asn315 |
| Streptomycin 3’-kinase P18150 (*Streptomyces griseus*) | NS |  | Arg60 | Arg60 | Asp210 | Asp210 | Asp190 Asn195 |
| Kanamycin kinase (Aminoglycoside-OH) 1L8T (*Enterococcus faecalis*) | Ser91 2.294 Met90 4.618 | 3.225 | Lys44 1.770 | Lys44 2.071 | Asp208 2.711 | Asp208 2.159 | Asp190 Asn195 |
| Methylthioribose kinase (APS^9^-ribose-OH) 2OLC (*Bacillus subtilis*) | Glu1151.786 Met114 4.452 | 3.052 | Lys61 1.750 | Lys61 2.508 | Asp250 3.243 | Asp250 2.492 | Asp233  His235 Open |
| Viomycin kinase^7^ D2B3F1 (*Streptosporangium roseum*) | NS |  | Arg61 | Arg61 | Asp211 | Asp211 | His187 Asp189 |
| [Hydroxymetylglutaryl-CoA reductase (NADPH_2_)] kinase | NS |  |  |  |  |  |  |
| Tyrosine kinase (Tyrosine-OH) 1U54 (*Homo sapiens*) | Gln206 2.113 Met203 8.518 | 3.068 | Lys158 2.762 | Lys158 1.944 | Asp270 3.104 | Asp270 2.421 | Asn252 Asn257 Arg356 |
| [Isocitrate dehydrogenase (NADP^+^)] kinase A3RX03 (*Ralstonia solanacearum*) prokaryote | NS |  | Arg225 | Arg225 | Asp399 | Asp399 | His376 Asp377 |
| [Myosin light-chain] kinase^7^ Q9H8B3 (*Homo sapiens*) | NS |  |  |  | Asp286 | Asp286 | His271 Asp274 |
| Hygromycin-B kinase^7^ P09979 (*Streptomyces hygroscopicus*) | NS |  |  |  |  |  |  |
| Calmodulin dependent protein kinase (Ser/Thr-OH) 3LIJ | Glu139 1.924 Met138 4.446 | 3.818 | Lys91 2.864 | Lys91 7.441 | Asp205 5.362 | Asp205 2.578 | Glu188  Asn189 Lys185 |
| Rhodopsin kinase (Ser/Thr-OH) 3C4W (*Bos taurus*) | Thr265 1.711 Met264 4.297 | 2.788 | Lys216 2.221 | Lys216 2.270 | Asp332 3.008 | Asp332 2.074 | Asp314  Asn319 Lys316 |
| β-Adrenergic-receptor kinase 1YM7 (*Bos taurus*) | No ADP |  |  |  |  |  |  |
| [Myosin heavy-chain] kinase^7^ Q4CQ29 (*Trypanosoma cruzi*) | NS |  |  |  |  |  |  |
| [Tau protein] kinase 0050321 (*Homo sapiens*) | NS |  |  |  |  |  |  |
| Macrolide 2’-kinase^7^ Q47396 (*Escherichia coli*) |  |  |  |  |  |  |  |
| 1 Phosphotidylinositol 3-kinase (Inositol-OH) 1E8X (*Sus scrofa*) wild boar | Gln288Cα  2.776 | 3.117 | Lys833 2.457 | Lys833 3.122 | Asp964 3.423 | Asp964 2.799 | Asp950 Asn951  His948 |
| [RNA-polymerase]-subunit kinase^7^ Q4A3R1 (Potato virus S) | NS |  |  |  |  |  |  |
| 1 Phosphotidylinositol 4,5-bisphosphate 3-kinase 2B3R (*Mus musculus*) | No ADP |  |  |  |  |  |  |
| 1-Phosphotidylinositol-4-phosphate 5-kinase 1BO1 (*Homo sapiens*) | No ADP |  | Lys214 | Lys214 | Asp369 |  | Asp278  Lys218 |
| **Lipid kinases** | | | | | | | |
| 1D-Myoinositol triphosphate kinase 1W2C (*Homo sapiens*) | Gln249  1.756 | 2.818 | Lys209 1.855 | Lys209 2.428 | Asp416 3.527 | Mn^2+^ coordinated | Glu262  Lys264 |
| Inositol tetrakiphosphate 5-kinase | NS |  |  |  |  |  |  |
| 1-Phosphotidylinositol-5-phosphate 4-kinase^7^ P78356 (*Homo sapiens*) | NS |  | Lys214 | Lys214 | Asp269 | Asp269 | Asp278 Lys218 |
| 1-Phosphotidylinositol-3-phosphate 5-kinase^7^ Q9Y217 (*Homo sapiens*) | NS |  |  |  |  |  |  |
| Inositol-polyphosphate multikinase 2IF8 ([*Saccharomyces cerevisiae*)](http://www.rcsb.org/pdb/search/smartSubquery.do?smartSearchSubtype=TreeEntityQuery&t=1&n=4932) | Gln118Cα 1.636 | 2.847 | Lys31 3.933 | Lys31 1.861 | Asp325 2.677 | No Mg^2+^ | Glu37  Lys133  His328 |
| 1-Phosphotidylinositol 4-phosphate 5-kinase 2GK9 (*Homo sapiens*) | No ADP |  | Lys167 | Lys167 | Asp227 | Asp227 | Asp208  Lys171 |
| **ATP-grasp fold** | | | | | | | |
| Inositol tetrakiphosphate 1-kinase 2QB5 (*Homo sapiens*) | Gln188Cα 1.879 | 2.853 | Lys157 1.543 | Distorted | Asp295 3.423 | Mn^2+^ coordinated | Asn297  His162 His167  His233 |
| Pyruvate phosphate dikinase 1DIK ([*Clostridium symbiosum*)](http://www.rcsb.org/pdb/search/smartSubquery.do?smartSearchSubtype=TreeEntityQuery&t=1&n=1512) | No ADP |  |  |  |  |  |  |
| Pyruvate, water dikinase^7^ Q8TKJ7 (*Methanosarcina acetivorans*) | NS |  |  |  |  |  |  |

| **Kinase** | **Met-S or Gln-C=O to Aden-NH_2_^1^** | **C8-H to α-PO_4_** | **Lys-NH_3_ to α-PO_4_^2^** | **Lys-NH_3_ to β-PO_4_^2^** | **Asp-COOH to β-PO_4_^3^** | **Asp-COOH to Mg^2+,3^** | **Substrate H^+^ removal^4,5,6^** |
| --- | --- | --- | --- | --- | --- | --- | --- |
| Phosphomevalonate kinase 3GON **(FROM GROUP 2)** | 50  4.557 | 2.715 | 4.57 | 3.916 | Distorted | Distorted |  |
|  |  |  |  |  |  |  |  |
| **Mean^10^** | **2.082** | **3.050** | **2.293** | **2.545** | **2.984** | **2.373** |  |
| **Standard Deviation** | **0.328** | **0.271** | **0.667** | **0.605** | **0.490** | **0.242** |  |
| **% Standard Deviation** | **20.561** | **8.887** | **29.105** | **23.770** | **16.429** | **10.218** |  |
| Cyclin dependent kinase-2 3JST (1Mn^2+^) | Glu81 1.568 | 2.581 | Lys33  2.921 | Lys33 1.757 | Asp127 8.312 | α-β-PO_4_ Mn^2+^ 5.299 |  |
| Cyclin dependent kinase-2 3QHW (3Mg^2+^) | Glu81 1.739 | 2.937 | Lys33 1.744 | Lys33 2.388 | Asp127 5.940 | α-β-PO_4_ Mn^2+^ 4.674 |  |

1. Coordination of the carbonyl of the backbone carbon and the thiol ether of Met to the hydrogen of the N6-NH_2_ and C8 respectively.
2. The coordinated Lys acts to transfer the H^+^ from the α-PO_4_ to the β-PO_4_.
3. Asp to β-PO_4_ coordinated and Asp is Mg^2+^
4. Residues associated with the deprotonation of the substrate.
5. Residues associated with the deprotonation of the substrate.
6. Residues associated with the deprotonation of the substrate.
7. Residues identified by sequence alignment using UniProt sequence (Accession Number as indicated).

NS = No structure in PDB.

**Figure MECH1.** Phosphoryl transfer mechanism found in the Group 1 kinases. Within the Protein kinase superfamily and protein kinase family a conserved methionine residue is found within the active site. It is proposed that a water molecule is hydrogen bonded between the sulphur of the methionine and C8 of the adenyl ring creating the proton addition complex analogous to the Wheland intermediate implicated in electrophilic aromatic substitution. The coordination of the backbone carbonyl to C6-NH_2_ creates a “push” mechanism that renders the C8-H more acidic, releasing the H^+^ to the oxygen of α-PO_4_. The “push” mechanism requires the protonation of C8 as part of rendering the C8-H more acidic. The C8-H is within H-bonding distance of the α-PO_4_ (3.050 ± 0.271 Å) and the H^+^ is transferred to the α-PO_4_, allowing for the effective transfer of the H^+^ to the β-PO_4_ via a coordinating lysine or arginine residue. The mean interatomic distances between the Lys residue and the α- and β-PO_4_ moieties are 2.293 ± 0.66 Å and 2.545 ± 0.605 Å, respectively. The protonation of the α-PO_4_ and a concomitant transfer of an H^+^ from the α-PO_4_ to β-PO_4_ via a conserved Lys facilitates the γ-PO_4_ becoming activated for nucleophilic attack. There is a concomitant deprotonation of the substrate-OH via a conserved Asp. This allows for the nucleophilic attack by the substrate, creating the pentavalent intermediate, and the phosphoryl transfer. The protonated Asp then transfers the proton to the γ-PO_4_. This changes the coordination of the Mg^2+^ from being β-PO_4_ to γ-PO_4_ coordinated to being α-PO_4_ to β-PO_4_ coordinated. The H^+^ originally arising from the C8 is then transferred back to C8, allowing the electron density of the adenyl moiety to return to the “ground-state” distribution.

**Table AF 1B.** Group 1 kinases.

|  | | **SUPERFAMILY^1^** | | **FAMILY / DOMAIN^2^** |
| --- | --- | --- | --- | --- |
| **Group 1 Kinases: Ser/Thr-Tyr** | | | | |
| 1. Protein Ser/Thr kinase (pdb2BZK) | | Protein kinase (CL0016) | | Protein kinase domain (PF00069) |
| 1. Phosphorylase kinase (pdb2PHK) | | Protein kinase (CL0016) | | Protein kinase domain (PF00069) |
| 1. Calmodulin-dependent protein kinase (pdb3LIJ) | | 1. Protein kinase (CL0016) 2. EF-hand like (CL0220) | | 1. Protein kinase domain (PF00069) 2. EF hand (x3) (PF00036) |
| 1. Rhodopsin kinase (pdb3C4W) | | 1. Protein kinase (CL0016) 2. RGS-like (CL0272) | | 1. Protein kinase domain (PF00069) 2. Regulator of G protein signaling domain (PF00615) |
| 1. β-Adrenergic-receptor kinase (pdb1YM7) | | 1. Protein kinase (CL0016) 2. RGS-like (CL0272) 3. PH domain-like | | 1. Protein kinase domain (PF00069) 2. Regulator of G protein signaling domain (PF00615) 3. PH domain (PF00169) |
| 1. Tyrosine kinase (pdb1U54) | | Protein kinase (CL0016) | | Protein tyrosine kinase (PF00069) |
| 1. Choline kinase (pdb3GI5) | | Protein kinase (CL0016) | | Choline/ethanolamine kinase (PF01633) |
| 1. Ethanolamine kinase (uniprotQ9HBU6) | | Protein kinase (CL0016) | | Choline/ethanolamine kinase (PF01633) |
| 1. Streptomycin 3-kinase (uniprotP18150) | | Protein kinase (CL0016) | | Phosphotransferase enzyme family (PF01633) |
| 1. Viomycin kinase (uniprotD2B3F1) | | Protein kinase (CL0016) | | Phosphotransferase enzyme family (PF01633) |
| 1. Hygromycin-B kinase (uniprotP09979) | | Protein kinase (CL0016) | | Phosphotransferase enzyme family (PF01633) |
| 1. Kanamycin kinase (pdb1L8T) | | Protein kinase (CL0016) | | Phosphotransferase enzyme family (PF01633) |
| 1. Methylthioribose kinase (pdb2OLC) | | Protein kinase (CL0016) | | Phosphotransferase enzyme family (PF01633) |
| 1. Macrolide 2’-kinase (uniprotQ47396) | | Protein kinase (CL0016) | | Phosphotransferase enzyme family (PF01633) |
| 1. Streptomycin 6-kinase (uniprotP08077) | | Protein kinase (CL0016) | | Amino glycoside/hydrourea antibiotic resistance kinase (PF04655) |
| 1. [Myosin heay-chain] kinase (uniprotQ4CQ29) | | nd^3^ | | Alpha kinase family (PF02816) |
| 1. Homoserine kinase (uniprotP00547) | | Ribosomal protein S5domain 2-like superfamily (CL0329) | | 1. GHMP kinases N-terminal domain (PF00288) 2. GHMP kinases C-terminal domain (PF08544) |
| 1. [Myosin light-chain] kinase (uniprotQ9H8B3) | | Immunoglobulin superfamily (CL0011) | | Immunoglobulin I-set domain (PF07679) |
| 1. 1-Phosphatidylinositol 3-kinase (pdb1E8X) | | 1. Ubiquitin (CL0072) 2. C2 superfamily (CL0154) 3. Tetratrico peptide repeat (CL0020) 4. nd | | 1. PI3-kinase family, ras-binding domain (PF00794) 2. Phosphoinositide 3-kinase C2 (PF00792) 3. Phosphoinositide 3-kinase family, accessory domain (PIK domain) (PF00613) 4. Phosphatidylinositol 3- and 4-kinase (PF00454) |
| 1. 1-Phosphatidylinositol 4,5-bisphosphate 3-kinase (uniprotO00443) | | 1. Ubiquitin (CL0072) 2. C2 superfamily (CL0154) 3. Tetratrico peptide repeat (CL0020) 4. nd 5. nd 6. C2 superfamily (CL0154) | | PI3-kinase family, ras-binding domain (PF00794)Phosphatide 3-kinase C2 family (PF00792)Phosphoinositide 3-kinase family, accessory domain (PIK domain) (PF00613)Phosphatidylinositol 3- and 4-kinase (PF00454)PX domain (PF00787)C2 domain (PF00168) |
| 1. 1-Phosphatidylinositol 4-phosphate 5-kinase (pdb2GK9) | | nd | | Phosphatidylinositol-4-phosphate 5-kinase (PF01504) |
| 1. 1-Phosphatidylinositol 4-phosphate 5-kinase (pdb1BO1) | | nd | | Phosphatidylinositol-4-phosphate 5-kinase (PF01504) |
| 1. [Isocitrate dehydrogenase (NADP^+^)] kinase (uniprotA3RX03) | | nd | | Isocitrate dehydrogenase kinase/phosphatase (PF06315) |
| 1. [RNA-polymerase]-subunit kinase (uniprotQA3R1) | |  | |  |
| 1. [Hydroxynethylglutaryl-CoA reductase (NADPH_2_)] kinase | | No sequence | |  |
| 1. [Tau protein] kinase | | No sequence | |  |
| **Group 1 Kinases: Lipid kinases** | | | | |
| 1. 1D-Myoinositol triphosphate kinase (pdb1W2C) | nd | | Inositol polyphosphate kinase (PF03770) | |
| 1. Inositol-polyphosphate multikinase (pdb2IF8) | nd | | Inositol polyphosphate kinase (PF03770) | |
| 1. Inositol tetrakiphosphate 5-kinase (uniprotQ9LY23) | nd | | Inositol polyphosphate kinase (PF03770) | |
| 1. 1-Phosphatidylinositol-4-phosphate 5-kinase (pdb1BO1) | nd | | Phosphatidylinositol-4-phosphate 5-kinase (PF01504) | |
| 1. 1-Phosphatidylinositol-5-phosphate 4-kinase (uniprotP78356) | nd | | Phosphatidylinositol-4-phosphate 5-kinase (PF01504) | |
| 1. 1-Phosphatidylinositol-5-phosphate 5-kinase (uniprotQ9Y217) | nd | | FYVE zinc finger  DEP domain  TCP-1/cpn60 chaperonin family  Phosphatidylinositol-4-phosphate 5-Kinase | |
| **Group 1 Kinases: ATP-grasp fold** | | | | |
| 1. Inositol tetrakiphosphate 1-kinase (pdb2QB5) | ATP-grasp (CL0179) | | Inositol 1, 3, 4-trisphosphate 5/6-kinase (PF05770) | |
| 1. Pyruvate phosphate dikinase (pdb1DIK) | 1. nd 2. nd 3. Pyruvate kinase-like TIM barrel superfamily (CL0151) | | Pyruvate phosphate dikinase, PEP/pyruvate binding domain (PF01326)PEP-utilising enzyme, mobile domain (PF00391)PEP-utilising enzyme, TIM barrel domain (PF02896) | |
| 1. Pyruvate water dikinase (uniprot Q8TKJ7) | 1. nd 2. nd 3. Pyruvate kinase-like TIM barrel superfamily (CL0151) | | Pyruvate phosphate dikinase, PEP/pyruvate binding domain (PF01326)PEP-utilising enzyme, mobile domain (PF00391)PEP-utilising enzyme, TIM barrel domain (PF02896) | |
| 1. Phosphomevalonate kinase (pdb3GON) | 1. Ribosomal protein S5 domain 2-like (CL0329) 2. nd | | 1. GHMP kinases N terminal domain(PF00288) 2. GHMP kinases C terminal (PF08544) | |

^1^ pfam clan classification in brackets

^2^ pfam family/domain classification in brackets

^3^ No Detectable (nd) similarity to conventional kinases

Where there are 2 or domains recognised, these are denoted by ‘a’, ‘b’, etc. One domain has been selected to position the protein within the table.


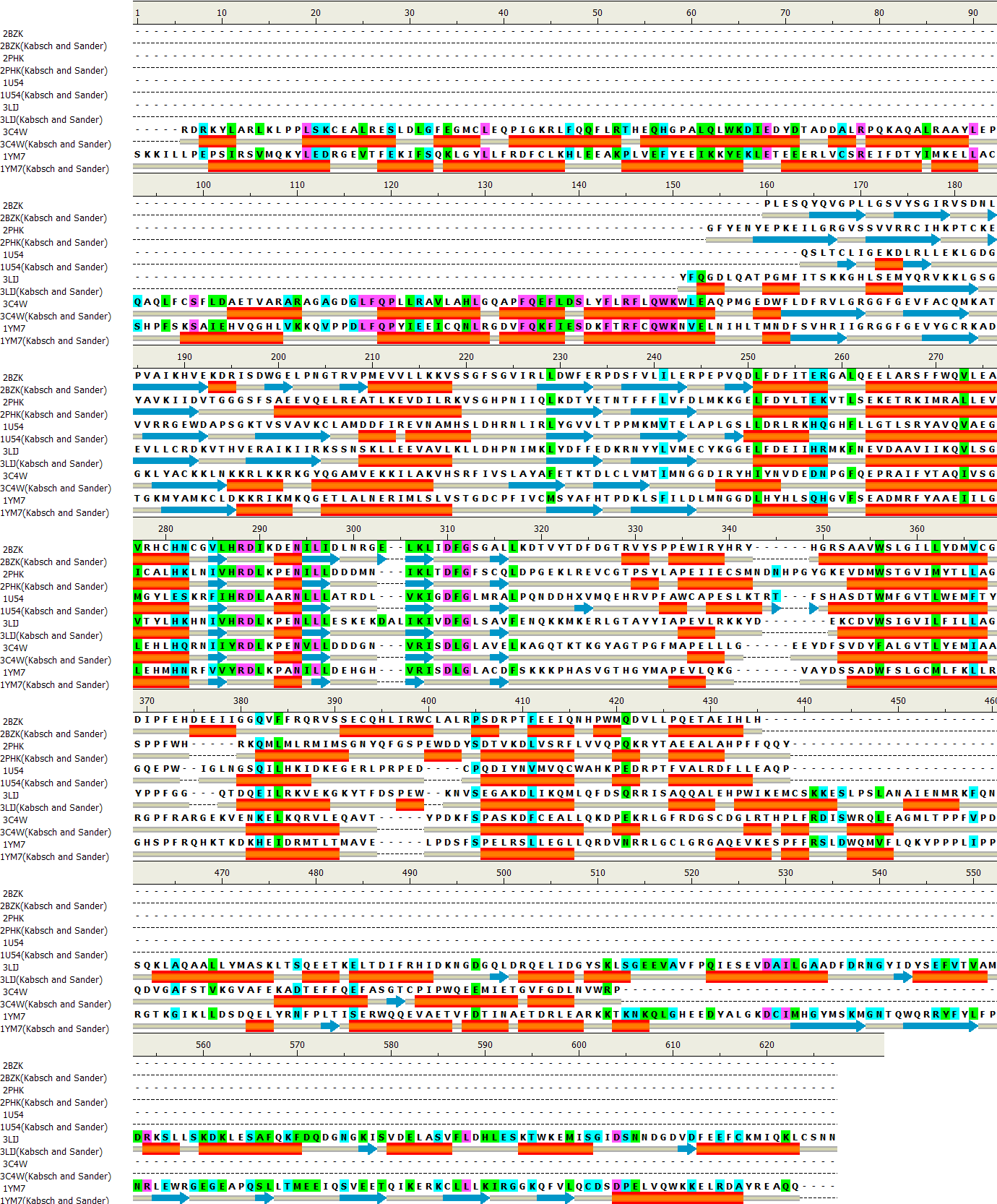


**↓ ↓ ↓ ↓**

**2 1**

**4 6 5 3**

**Figure SEQ1A.** Conserved functionality of selected **Group 1** kinase enzymes from within the ‘Protein Kinase’ superfamily and ‘protein kinase domain’ family. 2BZK, Protein Ser/Thr kinase; 2PHK, phosphorylase kinase; 1U54, tyrosine kinase; 3LIJ, calmodulin dependent protin kinase; 3C4W, rhodopsin kinase; 1YM7, β-adrenergic-receptor kinase. Pink = 100% identical, Green = >75% identical, turquoise = >50% identical. The indicated numbering is as per the identified residues as outlined in Sup Inf Table SI 1A. Secondary structure elements; Orange/Pink tube = Helix, Blue Arrow = Sheet, Grey = Coil. The total percentage of identical residues in this alignment is 21.45%.


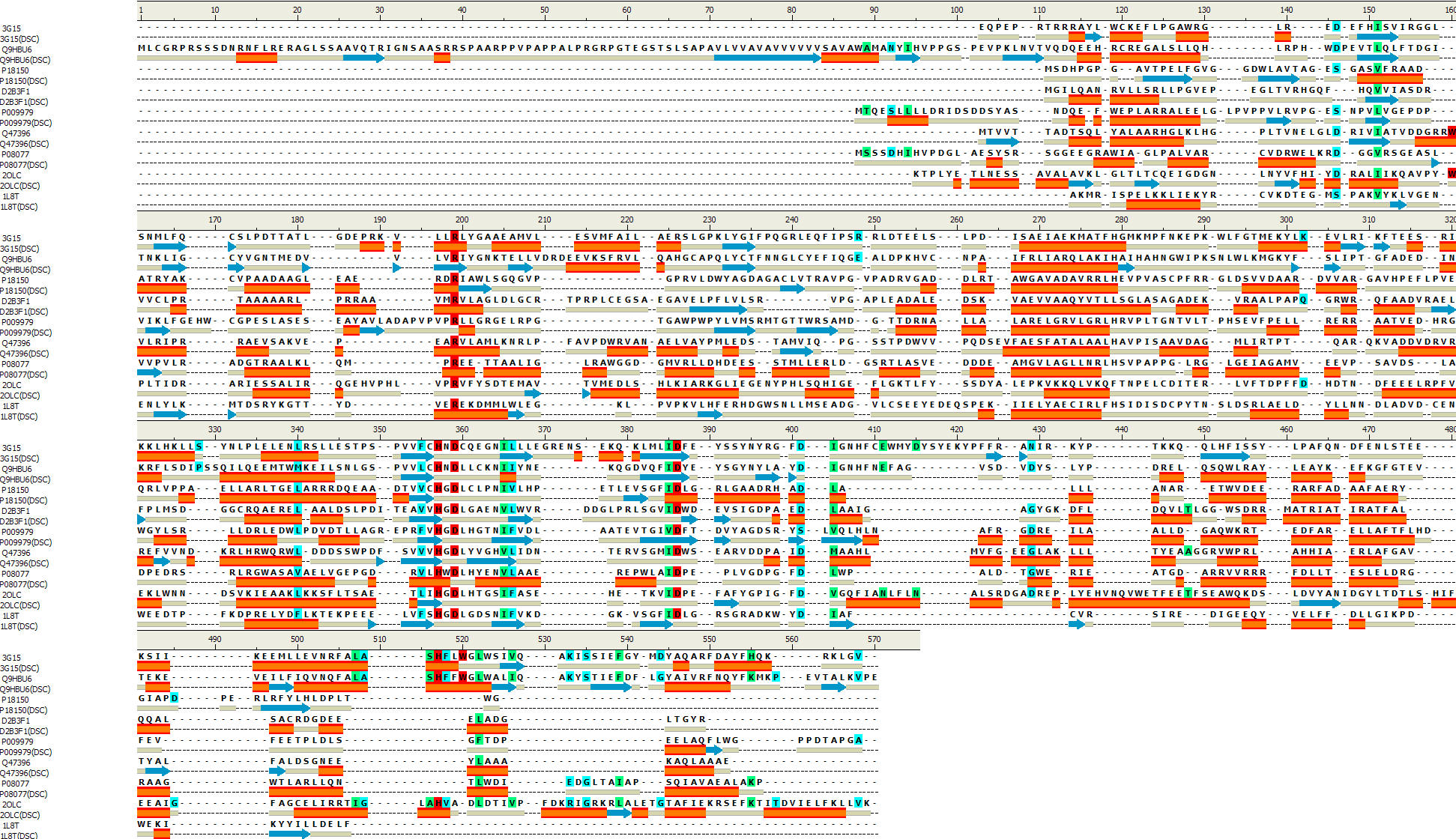


**6 4 3 6**

**↓ ↓ ↓ ↓**

**2**

**↓**

**Figure SEQ1B.** Conserved functionality of **Group 1** kinases that fall within the ‘Protein Kinase’ superfamily and ‘choline/ ethanolamine kinase’, ‘amino glycoside/hydrourea antibiotic resistance kinase’ and ‘phosphotransferase’ families. 3GI5, choline kinase; Q9HBU6, ethanolamine kinase; P18150, streptomycin 3’-kinase; D2B3F1, viomycin kinase; P09979, Hygromycin-B kinase; Q47396, macrolide 2’kinase; P08077, streptomycin 3’-kinase; 2OLC, methylthioribose kinase; 1L8T, kanamycin kinase. The indicated numbering is as per the identified residues as outlined in Sup Inf Table SI 1A, except for 6 which indicates conserved His and Asp/Glu residues responsible for stabilization involved in Mg^2+^ binding and substrate deprotonation. The total percentage of identical residues in this alignment is 22.87%.

**2**

**↓**


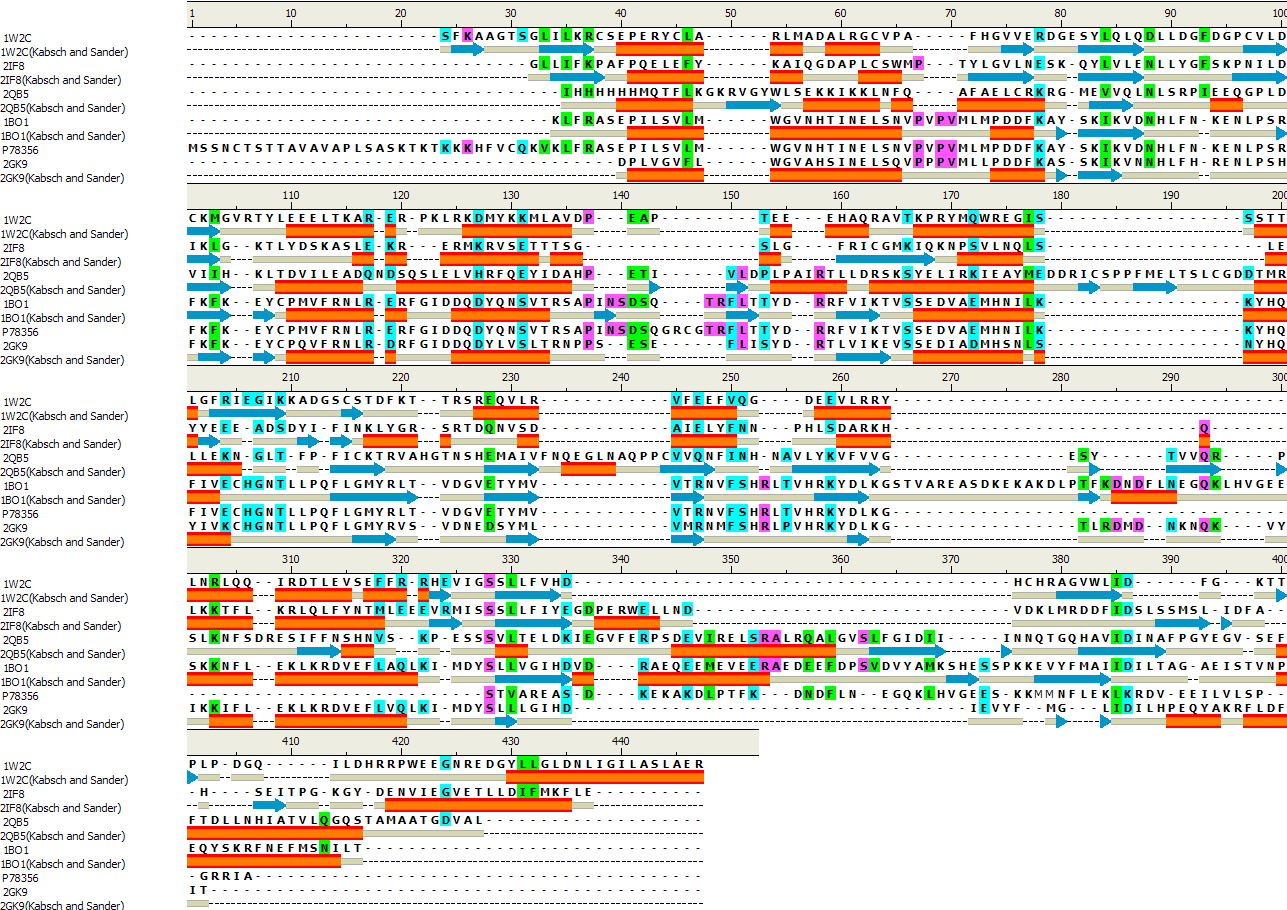


**2 2**

**4 3**

**↓ ↓**

**↓ ↓**

**Figure SEQ1C.** Conserved functionality within the ‘phosphatidylinositol-4-phosphate 5-kinase’ and the ‘inositol polyphosphate kinase’ families in **Group 1**. 1W2C, 1D-myoinositol triphosphate kinase; 2IF8, inositol-polyphosphate multikinase; 2QB5, inositol tetraphosphate 1-kinase; 1BO1, 1-phosphotidylinositol-4-phosphate 5-kinase; 2GK9, 1-phosphotidylinositol-4-phosphate 5-kinase; P78356, 1-phosphotidylinositol-5-phosphate 4-kinase. Pink = 100% identical, Green = >75% identical, turquoise = >50% identical. The indicated numbering is as per the identified residues as outlined in Sup Inf Table SI 1A. Secondary structure elements; Orange/Pink tube = Helix, Blue Arrow = Sheet, Grey = Coil. The indicated numbering is as per the identified residues as outlined in Sup Inf Table SI 1A. The total percentage of identical residues in this alignment is 22.87%.

**Table AF 2A.** Kinases representing **Group 2** and the identified conserved amino acid residues associated with the catalysis of phosphoryl transfer and the measured interatomic distances are shown. The PDB or uniprot accession numbers are indicated. Conserved residues replaced by conserved functionality are indicated by 3-letter code.

| **Kinase** | **αC-C=O to Aden-NH_2_^14^** | **N7-H to Arg^1^** | **C8-H to Arg^1^** | **C8-H to α-PO_4_** | **Arg-NH_3_ to α-PO_4_^2^** | **Arg-NH_3_ to β-PO_4_^2^** | **Lys-NH_3_ to γ-PO_4_ ^3^** | **Thr to C8-H^4^** | **Thr to α -PO_4_^4^** | **Arg to C8-H^5^** | **Thr to C8-H^6^** |
| --- | --- | --- | --- | --- | --- | --- | --- | --- | --- | --- | --- |
| **Rossmann-like kinases** |  | | | | | | | | | | |
| Gluconate kinase^1L^ 1KO5 ([*Escherichia coli*)](http://www.rcsb.org/pdb/search/smartSubquery.do?smartSearchSubtype=TreeEntityQuery&t=1&n=562) | Gln158 1.971 | Arg120 3.273 | Arg120 3.692 | 3.495 Direct | Arg124 2.940 | Arg124 Distorted | Lys21 γ 1.734 | Ser22 7.268 | Ser22 | 5.24 | 4.21 |
| Phosphoribulokinase 1A7J ([*Rhodobacter sphaeroides*](http://www.rcsb.org/pdb/search/smartSubquery.do?smartSearchSubtype=TreeEntityQuery&t=1&n=1063) ) | No ADP |  |  |  |  |  |  |  |  |  |  |
| Thymidine kinase 2ORW ([*Thermotoga maritima*)](http://www.rcsb.org/pdb/search/smartSubquery.do?smartSearchSubtype=TreeEntityQuery&t=1&n=2336) | Val139 1.778 |  |  | 4.738β | Lys16 1.678 | Lys16 1.732 |  |  |  |  |  |
| Pantothenate kinase^1D^ 2ZS9 ([*Mycobacterium tuberculosis*)](http://www.rcsb.org/pdb/search/smartSubquery.do?smartSearchSubtype=TreeEntityQuery&t=1&n=1773) | NC | Arg238 4.556 | Arg238 3.299 | 3.395 | Arg238 1.640 | Arg238 2.349 | Lys103 β 2.023 | Arg238 |  | Arg238 3.299 | Arg238 |
| Uridine kinase^1L§^ 2UVQ ([*Homo sapiens*)](http://www.rcsb.org/pdb/search/smartSubquery.do?smartSearchSubtype=TreeEntityQuery&t=1&n=9606) | Asp215COOH 2.386 | Arg168 4.116 | Arg168 5.455 | 4.936 | Arg172 3.178 | Arg172 3.094 | Lys36  β 2.744 | Thr38 3.324 | Thr38 2.591 | Arg168 3.827 | Thr38 3.526 |
| Shikimate kinase^1L^ 1L4U ([*Mycobacterium tuberculosis*)](http://www.rcsb.org/pdb/search/smartSubquery.do?smartSearchSubtype=TreeEntityQuery&t=1&n=1773) | Arg153 1.959 | Arg110 3.799 | Arg110 4.012 | 3.697 | Arg117 2.757 | Arg117 1.783 | Lys15  β-γ | Thr17 3.469 | Thr17 2.767 | Arg110 3.037 | Glu31 3.113 |
| 6-Phosphofructo-2-kinase 2I1V ([*Homo sapiens*)](http://www.rcsb.org/pdb/search/smartSubquery.do?smartSearchSubtype=TreeEntityQuery&t=1&n=9606) | Not Group 2 |  |  | 2.661 | Lys168 4.237 | Lys138 4.237 | Lys47 |  |  |  |  |
| Deoxyguasine kinase^1D^ 2JAS ([*Mycoplasma mycoides subsp.mycoides*](http://www.rcsb.org/pdb/search/smartSubquery.do?smartSearchSubtype=TreeEntityQuery&t=1&n=44101) ) | Asp83 1.611 | Arg78 4.104 | Arg78 2.492 | 5.310 | Lys13 1.890 | Lys13 1.820 | Lys13  α-γ | Glu 31 2.592 |  | Arg78 2.426 | Glu31 3.113 |
| Deoxyribonucleoside kinase 2JCS ([*Drosophila melanogaster*)](http://www.rcsb.org/pdb/search/smartSubquery.do?smartSearchSubtype=TreeEntityQuery&t=1&n=7227) | No ADP | Arg94 | Arg94 |  | Lys22 | Lys22 | Lys22 | Asp41 |  |  |  |
| Polyphosphate kinase 1XDP ([*Escherichia coli*)](http://www.rcsb.org/pdb/search/smartSubquery.do?smartSearchSubtype=TreeEntityQuery&t=1&n=562) | GROUP 3/11 |  |  |  |  |  |  |  |  |  |  |
| Phosphomevalonate kinase 3GON **(**[*Streptococcus pneumoniae* R6)](http://www.rcsb.org/pdb/search/smartSubquery.do?smartSearchSubtype=TreeEntityQuery&t=1&n=171101) | GROUP 1 |  |  |  |  |  |  |  |  |  |  |
| Adenylate kinase 2C95^1L^ ([*Homo sapiens*)](http://www.rcsb.org/pdb/search/smartSubquery.do?smartSearchSubtype=TreeEntityQuery&t=1&n=9606) | Gly177 1.906 | Arg128 4.372 | Arg128 4.789 | 4.042 | Arg132 4.958 | Arg132 2.136 | Lys21 γ 1.865 | Thr23 2.228 | Thr23 3.621 | Arg128 3.795 | Arg23 2.249 |
| Cytidylate kina+se 1QF9^1L^ ([*Dictyostelium discoideum*)](http://www.rcsb.org/pdb/search/smartSubquery.do?smartSearchSubtype=TreeEntityQuery&t=1&n=44689) | Arg176 1.812 | Arg127 4.076 | Arg127 4.734 | 3.338 | Arg131 2.018 | Arg131 3.720 | Lys19 γ 2.139 | Thr21 2.381 | Thr21 2.739 | Arg127 3.747 | Arg21 2.486 |
| **Phosphoenolpyruvate carboxykinase** |  | | | | | | | | | | |
| Protein kinase (HPr kinase)^10,1D^ 2GBL ([*Synechococcus elongatus*)](http://www.rcsb.org/pdb/search/smartSubquery.do?smartSearchSubtype=TreeEntityQuery&t=1&n=1140) | SerA330 1.249 | A326 3.618 | A326 3.987 | 4.643 | B459 4.055 | B459 2.147 | A294 |  |  | A319 3.359 | 4.409α 3.253β |
| Protein kinase (HPr kinase)^10^ 2GBL | SerA89 3.428 | A88 away from site | A88 away from site | 5.617 | B226 3.799 | B226 1.853 | A52 |  |  |  |  |
| Phosphoenolpyruvate carboxykinase 1AQ2 ([*Escherichia coli*)](http://www.rcsb.org/pdb/search/smartSubquery.do?smartSearchSubtype=TreeEntityQuery&t=1&n=562) | Ile450 1.623 | Arg449 4.541 | Arg449 4.291 | 5.461 | Arg333 4.491 | Arg333 4.605 | Lys254 β-γ | Thr256 3.512 | Thr256 3.858 |  | 2.093β 2.130γ |
| Glucokinase 3FGU FROM RIBOKINASE ([*Homo sapiens*)](http://www.rcsb.org/pdb/search/smartSubquery.do?smartSearchSubtype=TreeEntityQuery&t=1&n=9606) | Leu415 3.861 | Ser411 4.287 | Ser411 4.409 | 5.583 | Arg85 3.981 | Arg85 4.382 | Lys169 | Ser411 4.395 | Ser411 3.621 |  |  |
|  |  |  |  |  |  |  |  |  |  |  |  |
| **Mean** | **2.101** | **4.098** | **4.140** | **4.332** | **2.953** | **2.847** | **2.073** | **3.797** | **3.200** | **3.260** | **3.028** |
| **Standard Deviation** | **0.699** | **0.401** | **1.036** | **1.010** | **1.197** | **1.143** | **0.480** | **1.697** | **0.558** | **0.568** | **0.485** |
| **% Standard Deviation** | **33.28** | **9.77** | **25.02** | **23.32** | **40.52** | **40.15** | **23.16** | **44.70** | **17.45** | **17.415** | **16.022** |
| **Phosphoglycerate kinase^8^** | Reverse reaction |  |  |  |  |  |  |  |  |  |  |
| **Kinase** | **Gln-αC-C=O to Aden-NH_2_** | **N7 to backbone NH** | **Asp to N7** | **C8-H to α-PO_4_** | **Lys-NH_3_ to α-PO_4_^2^** | **Lys-NH_3_ to β-PO_4_^2^** | **Lys-NH_3_ to γ-PO_4_ ^3^** | **Thr to C8-H^4^** | **Thr to α-PO_4_^4^** |  | **Thr to C8-H^5a^** |
| **Aspartokinase** |  | | | | | | | | | | |
| Carbamate kinase 1WE5 ([*Escherichia coli*)](http://www.rcsb.org/pdb/search/smartSubquery.do?smartSearchSubtype=TreeEntityQuery&t=1&n=562) | His262 1.812 | Ala264 2.589 | Tyr328 4.322 | 6.684 | Lys271 4.012 | Lys272 1.788 | Lys209 | Ser267 2.812 | Ser267 5.622 |  | Ser267 2.690 |
| Aspartokinase 2J0W ([*Escherichia coli*)](http://www.rcsb.org/pdb/search/smartSubquery.do?smartSearchSubtype=TreeEntityQuery&t=1&n=562) | Tyr227 2.167 | Lys257 3.619 | Asp230 4.133 | 7.498 | Lys257 2.243 | Lys257 5.107 |  | Asp230 2.509 | Asp230 8.006 |  | 2.065 |
| Acetylglutamate kinase 1OH9 ([*Escherichia coli*)](http://www.rcsb.org/pdb/search/smartSubquery.do?smartSearchSubtype=TreeEntityQuery&t=1&n=562) | Leu209 1.847 | Thr211 2.220 | Thr 211 2.950 | 6.674 | Lys217 3.623 | Lys217 2.355 | Lys58 | Asp212 1.917 | Asp212 3.731 |  | 1.993 |
| Uridylate Kinase 2BRI ([*Pyrococcus furiosus*)](http://www.rcsb.org/pdb/search/smartSubquery.do?smartSearchSubtype=TreeEntityQuery&t=1&n=2261) | Tyr146 1.882 | Asp149 2.894 | Asp149 3.820 | 7.671 | Arg49 | Arg49 | Arg49 | Ser181 | Ser181 |  |  |
| **Mean** | **1.927** | **2.831** | **3.806** | **7.271** | **3.159** | **2.758** |  | **2.677** | **5.032** |  | **2.554** |
| **Standard Deviation** | **0.163** | **0.594** | **0.607** | **0.435** | **0.805** | **1.589** |  | **0.646** | **2.311** |  | **0.685** |
| **% Standard Deviation** | **8.787** | **20.969** | **15.953** | **5.988** | **25.481** | **57.597** |  | **24.128** | **45.921** |  | **26.836** |

| **Kinase** | **Gln- αC-C=O to Aden-NH_2_** | **C8-H to Ser** | **Ser to α-PO_4_** | **C8-H to α-PO_4_** | **Thr-OH to α-PO_4_^2^** | **Thr-OH to β-PO_4_^2^** | **H-bonding to N7^1^** | **Asp to substrate-OH** | **Asp to γ-PO_4_** | **C8 to Backbone C=O^13^** | **C8-H to α-PO_4_^6^** |
| --- | --- | --- | --- | --- | --- | --- | --- | --- | --- | --- | --- |
| **Phosphofructokinase** |  |  |  |  |  |  |  |  |  |  |  |
| 6-Phosphofructokinase 4PFK ([*Geobacillus stearothermophilus*)](http://www.rcsb.org/pdb/search/smartSubquery.do?smartSearchSubtype=TreeEntityQuery&t=1&n=1422) | Gln107 3.504 | Direct | Direct | 3.265 | Arg72 1.793 | Arg72 1.886 | Lys77 3.619 | No transfer group | No transfer group | Gly104 3.046 |  |
| 6-Phosphofructokinase 3F5M ([*Trypanosoma brucei*)](http://www.rcsb.org/pdb/search/smartSubquery.do?smartSearchSubtype=TreeEntityQuery&t=1&n=5691) | Arg203NH2 1.905 | Direct | Direct | 3.427 | Arg173 1.922 | Arg173 3.981 | Arg203 4.257 | No transfer group | No transfer group | Gly200 3.518 |  |
| 1-Phosphofructokinase **2AJR**/2JG5^7^ ([*Thermotoga maritima*)](http://www.rcsb.org/pdb/search/smartSubquery.do?smartSearchSubtype=TreeEntityQuery&t=1&n=2336) | No ADP |  |  |  | Ser68 | Ser68 | Arg105 |  |  | Gly102 |  |
| Diphosphate-fructose-6-phosphate 1-phosphotransferase **3K2Q**/3HN0/1KZH^7^ ([*Marinobacter aquaeolei* VT8)](http://www.rcsb.org/pdb/search/smartSubquery.do?smartSearchSubtype=TreeEntityQuery&t=1&n=351348) | No ADP |  |  |  | Arg78 | Arg78 | Arg81 |  |  | Gly114 |  |
| Diacylglycerol kinase 2QV7^7^ ([*Staphylococcus aureus*)](http://www.rcsb.org/pdb/search/smartSubquery.do?smartSearchSubtype=TreeEntityQuery&t=1&n=1280) | Open site |  |  |  | Thr63 | Thr63 | Asn94 |  |  | Gly91 |  |
| **Kinase** | **Gln- αC-C=O to Aden-NH_2_** | **C8-H to Ser** | **Ser to α-PO_4_** | **C8-H to α-PO_4_** | **Thr-OH to α-PO_4_^1^** | **Thr-OH to β-PO_4_^1^** | **H-bonding to N7^2^** | **Asp to substrate-OH^3R^** | **Asp to γ-PO_4_^14^** | **C8 to Backbone C=O^13^** | **C8-H to α-PO_4_^6^** |
| **Ribokinase** |  |  |  |  |  |  |  |  |  |  |  |
| Ketohexokinase 3HQQ ([*Leishmania mexicana*)](http://www.rcsb.org/pdb/search/smartSubquery.do?smartSearchSubtype=TreeEntityQuery&t=1&n=5665) | No ADP |  |  |  | Thr253 | Thr253 |  | Asp258 |  |  |  |
| 4-Fructokinase 3EPQ ([*Bacillus subtilis*)](http://www.rcsb.org/pdb/search/smartSubquery.do?smartSearchSubtype=TreeEntityQuery&t=1&n=1423) | NC | Direct | Direct | 3.041 | Thr130 4.827 | Thr130 1.757 | Gln233 2.619 | Asp103 NS | Asp103 3.617 | Gly230 3.143 | 2.880 |
| Ribokinase 1RKD ([*Escherichia coli*)](http://www.rcsb.org/pdb/search/smartSubquery.do?smartSearchSubtype=TreeEntityQuery&t=1&n=562) | NC | Direct | Direct | 3.279 | Thr223 2.276 | Thr223 3.972 | Thr250 3.437 | Asp255 2.703 | Asp255 3.244 | Gly225 3.673 | 3.620 |
| Adenosine kinase 2PKN ([*Mycobacterium tuberculosis*)](http://www.rcsb.org/pdb/search/smartSubquery.do?smartSearchSubtype=TreeEntityQuery&t=1&n=1773) | NC | Direct | Direct | 3.182 | Thr223 3.036 | Thr223 4.371 | Thr253 | Asp257 NS | Asp257 5.936 | Gly225 3.385 | 3.705 |
| Pyridoxal kinase 3IBQ (*Lactobacillus plantarum*) | Leu204 1.834 | Direct | Direct | 2.464 | Thr211 2.292 | Thr211 2.486 | Arg203 6.737 | Asp213 NS | Asp213 4.918 |  | 2.464 |
| 2-keto-3-deoxygluconate kinase 3KTN^7^ ([*Lactobacillus plantarum*)](http://www.rcsb.org/pdb/search/smartSubquery.do?smartSearchSubtype=TreeEntityQuery&t=1&n=1590) | No ADP |  |  |  | Thr239 | Thr239 |  | Asp278 | Asp278 |  |  |
| Hydroxyethyl thiazole kinase 3HPD^7^ ([*Pyrococcus horikoshii*)](http://www.rcsb.org/pdb/search/smartSubquery.do?smartSearchSubtype=TreeEntityQuery&t=1&n=70601) | No ADP |  |  |  |  |  |  |  |  |  |  |
| 1-phosphofructokinase 2AJR^7^ (([*Thermotoga maritima*)](http://www.rcsb.org/pdb/search/smartSubquery.do?smartSearchSubtype=TreeEntityQuery&t=1&n=2336) | No ADP |  |  |  |  |  |  |  |  |  |  |
| Tagatose-6-phosphate kinase **2JG1**/2JGV ([*Staphylococcus aureus subsp. aureus*)](http://www.rcsb.org/pdb/search/smartSubquery.do?smartSearchSubtype=TreeEntityQuery&t=1&n=93061) |  | Direct | Direct | 2.805 | Lys183 4.291 | Lys183 2.213 | Ser252 3.564 | Asp254 NS | Asp254 3.696 | Gly224 4.022 | 2.805 |
| ADP-dependent phosphofructokinase 3DRW^12^ ([*Pyrococcus horikoshii*)](http://www.rcsb.org/pdb/search/smartSubquery.do?smartSearchSubtype=TreeEntityQuery&t=1&n=53953) | Val422 1.943 | Direct | Direct | 3.167 | Asp287 3.079 | ADP | Thr428 3.542 | Asp433 | Asp433 |  |  |
| ADP-dependent glucokinase^11^ 1GC5 (*Thermococcus litoralis*) | Val440 2.106 | Direct | Direct |  | Glu306 4.28 | ADP | Thr446 3.621 | Asp451 | Glu451 |  |  |
| Phosphomethyl pyrimidine kinase 1UB0^7^ /1JXH ([*Thermus thermophilus*)](http://www.rcsb.org/pdb/search/smartSubquery.do?smartSearchSubtype=TreeEntityQuery&t=1&n=274) | No ADP |  |  |  |  |  | Thr208 | Cys210 | Cys210 |  |  |
| **Thiamin pyrophosphokinase** |  |  |  |  |  |  |  |  |  |  |  |
| Thiamin pyrophosphate kinase 2F17^12^ ([*Mus musculus*)](http://www.rcsb.org/pdb/search/smartSubquery.do?smartSearchSubtype=TreeEntityQuery&t=1&n=10090) | Gly129 3.241 | Direct | Direct | 2.331 | Asp46 3.074 | Pyr-PO_4_ | Arg131 4.983 | Asp73 | Asp73 | Asn25  3.811 |  |
| **Glycerate kinase** |  |  |  |  |  |  |  |  |  |  |  |
| Glycerate kinase 1TO6/1B8N/3CWC ([*Neisseria meningitidis* serogroup A](http://www.rcsb.org/pdb/search/smartSubquery.do?smartSearchSubtype=TreeEntityQuery&t=1&n=65699)) | No ADP |  |  |  |  |  |  |  |  |  |  |
| **Mean** | **2.865** |  |  | **3.200** | **3.163** | **3.086** | **4.042** |  | **4.429** | **3.390** | **3.337** |
| **Standard Deviation** | **1.812** |  |  | **0.799** | **0.979** | **0.961** | **1.366** |  | **1.235** | **0.398** | **0.398** |
| **% Standard Deviation** | **63.239** |  |  | **24.966** | **30.963** | **31.131** | **33.784** |  | **27.880** | **11.752** | **11.927** |

1. Arg/Lys/Thr coordinated to C8-H and N7
2. Arg/Lys coordinated to the α- and β-PO_4_
3. Lys responsible for the protonation of the γ-PO_4_
4. Thr/Ser coordinated to the C8-H of the ATP residue responsible for the protonation of C8 during the rehybridization change from sp2 to sp3
5. Arg to C8-H taking the hybridization change from sp2 to sp3 and the concomitant conversion of C8-H to C8-H_2_ into account.
6. Ser/Thr to C8-H taking the hybridization change from sp2 to sp3 and the concomitant conversion of C8-H to C8-H_2_ into account.
7. Residues identified by sequence alignment using UniProt sequence (Accession Number as indicated)
8. Carries out reverse reaction.
9. Location of the arginine residue relative to the pyrimidine ring, D = on the d-orientation of the ribose, L = on the l-orientation of the ribose.
10. Active site comprises 2 subunits with amino acid numbering prefixed by A and B (see group 7).
11. ADP-dependent kinase requires β-PO_4_ to be leaving group and therefore does not require proton transfer between α-PO_4_ and β-PO_4_.
12. Transfers pyrophosphate therefore requires β-PO_4_ to be leaving group and therefore does not require proton transfer between α-PO_4_ and β-PO_4_.
13. C8-H induction mechanism, comprising the amino acid and the adenyl nitrogen to which it is coordinated and the creation of a carbene.
14. Substrate coordinated Asp/Glu.
15. Coordination of backbone carbonyl to the adenyl C6-NH_2_.

§ adenyl group in crystal in *syn* conformation re-orientated to the *anti* conformation as found in the rest of the group.

NC = No coordinating residue.

IS = Incomplete structure

NS = No substrate.

**Figure MECH3.** Phosphoryl transfer mechanism found in the Group 2 kinases (Rossmann-like fold and phosphoenolpyruvte carboxykinase-like sequences). The initiation of phosphoryl occurs via the coordination of the ATP C6-NH_2_ to a carbonyl arising from the protein backbone by the “push” mechanism resulting in the protonation of C8 via the coordination of a conserved Arg. This renders the C8-H more acidic, allowing for the protonation of the α-PO_4_, via a conserved Ser/Thr carrier. There is a concomitant transfer of an H^+^ from the α-PO_4_ to β-PO_4_ via a conserved Arg, thereby facilitating the formation of the pentavalent intermediate between the γ-PO_4_ and the substrate nucleophile. There is a simultaneous ATP-mediated deprotonation of the substrate -OH, allowing for the nucleophilic attack by the substrate to create the pentavalent intermediate and allow phosphoryl transfer. A protonated Lys then transfers the proton to the γ-PO_4_, changing the transfer of the Mg^2+^ from being β-PO_4_ to γ-PO_4_ coordinated to being α-PO_4_ to β-PO_4_ coordinated. The H^+^ originally arising from the C8 is then transferred back to C8, allowing the electron density of the adenyl moiety to return to the “ground-state” distribution.

**Figure MECH4.** Phosphoryl transfer mechanism found in the Group 2 kinases (Aspartokinase-like sequences). Protonation of the C8 occurs via a backbone amide. The C8-H becomes more acidic, allowing for the protonation of the α-PO_4_ via a Ser or Asp residue. There is a concomitant transfer of an H^+^ from the α-PO_4_ to β-PO_4_ via a conserved Lys, facilitating the γ-PO_4_ as a leaving group. The substrate moiety to be phosphorylated is a carboxylate group and therefore does not require deprotonation for the nucleophilic attack by the substrate. The protonated Lys then transfers a proton to the γ-PO_4_, allowing the creation the pentavalent intermediate and subsequent phosphoryl transfer. A monovalent metal counter-ion such as K^+^ facilitates the transfer of the Mg^2+^ from being β-PO_4_ to γ-PO_4_ coordinated to being α-PO_4_ to β-PO_4_ coordinated. The H^+^ originally arising from the C8 is then transferred back to C8, allowing the hybridization of the adenyl moiety to return to the “ground-state” tautomer.

**Figure MECH5.** Phosphoryl transfer mechanism found in the Group 2 kinases (Ribokinase-like sequences). Generally within this group a backbone peptide bond is coordinated to the C6NH­­­_2_ and the N1. The protonation of N7 occurs via the coordination of a conserved Thr/Arg that renders the C8-H more acidic via the formation of a carbene, allowing for the direct protonation of the α-PO_4_ with a concomitant transfer of an H^+^ from the α-PO_4_ to β-PO_4_ via a conserved Thr/Ser/Lys. This facilitates the γ-PO_4_ as a leaving group. There is a concomitant Asp-mediated deprotonation of the substrate –OH, allowing for the nucleophilic attack by the substrate. In all the structures available the only structure containing the non-ATP substrate is ribokinase where there is not sufficient order to identify the amino acid responsible for the transfer a proton to the γ-PO_4_ creating the pentavalent intermediate and phosphoryl transfer. The proton arising from the substrate facilitates the transfer of the Mg^2+^ from being β-PO_4_ to γ-PO_4_ coordinated to being α-PO_4_ to β-PO_4_ coordinated. The H^+^ originally arising from the C8 is then transferred back to C8, allowing the electron density of the adenyl moiety to return to the “ground-state” distribution.

**Table AF 2B.** Group 2 kinases

|  | **SUPERFAMILY^1^** | **FAMILY / DOMAIN^2^** | **Mechanism** |
| --- | --- | --- | --- |
| **Group 2 kinases (Rossmann-like sequences)** | | | |
| 1. Gluconate kinase (pdb1KO5) | P-loop containing nucleoside triphosphate hydrolase | Shikimate kinase | 2A |
| 1. Phosphoribulokinase (pdb1A7J) | P-loop containing nucleoside triphosphate hydrolase | Phosphoribulokinase / Uridine kinase family | 2B |
| 1. Thymidine kinase (pdb2ORW) | P-loop containing nucleoside triphosphate hydrolase | Thymidine kinase | 2B |
| 1. Pantothenate kinase (pdb2ZS9) | P-loop containing nucleoside triphosphate hydrolase | Phosphoribulokinase / Uridine kinase family |  |
| 1. Uridine kinase (pdb2UVQ) | P-loop containing nucleoside triphosphate hydrolase | Phosphoribulokinase / Uridine kinase family | 2A |
| 1. Shikimate kinase (pdb1L4U) | P-loop containing nucleoside triphosphate hydrolase | Shikimate kinase | 2A |
| 1. 6-Phosphofructo-2-kinase (pdb2I1V) | P-loop containing nucleoside triphosphate hydrolase / Phosphoglycerate mutase-like | 6-phosphofructo-2-kinase / Phosphoglycerate mutase family |  |
| 1. Deoxyguanisine kinase (pdb2JAS) | P-loop containing nucleoside triphosphate hydrolase / Phosphoglycerate mutase-like | Deoxynucleoside kinase | 2B |
| 1. Deoxyribonuceoside kinase (pdb2JCS) | P-loop containing nucleoside triphosphate hydrolase / Phosphoglycerate mutase-like | Deoxynucleoside kinase | 2B |
| 1. Polyphosphate kinase (pdb1XDP) | n/a | Polyphosphate kinase |  |
| 1. Adenylate kinase (pdb2C95) | P-loop containing nucleoside triphosphate hydrolase /  Phosphoglycerate mutase-like | Adenylate kinase | 2A |
| 1. Cytidylate kinase (pdb1QF9) | P-loop containing nucleoside triphosphate hydrolase /  Phosphoglycerate mutase-like | Adenylate kinase | 2A |
| 1. HPr kinase (Protein kinase) (pdb2GBL) | P-loop containing nucleoside triphosphate hydrolase /  Phosphoglycerate mutase-like | KaiC |  |
| 1. Phosphoenolpyruvate carboxykinase (pdb1AQ2) | PEP carboxykinase-like | Phosphoenolpyruvate carboxykinase |  |
| 1. Glucokinase (pdb3FGU) | Actin-like ATPase | Hexokinase | 2B |
| **Group 2c (Aspartokinase )** | | | |
| 1. Carbamate kinase (pdb1WE5) | nd^3^ | Amino acid kinase |  |
| 1. Aspartokinase (pdb2J0W) | 1. nd 2. ACT-like domain | 1. Amino acid kinase family 2. ACT domain |  |
| 1. Aspartokinase (pdb2HMF) | 1. nd 2. ACT-like domain (x2) | - 1. Amino acid kinase family   2. ACT domain (x2) |  |
| 1. Acetylglutamate kinase (pdb1OH9) | 1. nd 2. ACT-like domain | 1. Amino acid kinase family 2. ACT domain |  |
| 1. Uridylate kinase (pdb2BRI) | 1. nd 2. ACT-like domain | 1. Amino acid kinase family 2. ACT domain |  |
| **Group 2d (Ribokinase-like )** | | | |
| 1. Glucokinase: (pdb3FGU) | Actin-like ATPase | Hexokinase |  |
| 1. Ketohexokinase: (pdb3HQQ). | Ribokinase-like | PkfB carbohydrate kinase |  |
| 1. 4-fructokinase: (pdb3EPQ) | Actin-like ATPase | ROK (Repressor, ORF, Kinase) |  |
| 1. Ribokinase: (pdb1RKD) | Ribokinase-like | PkfB carbohydrate kinase |  |
| 1. Adenosine kinase: (pdb2PKN) | Ribokinase-like | PkfB carbohydrate kinase |  |
| 1. Pyridoxal kinase: (pdb3IBQ) | Ribokinase-like | Phosphomethylpyrimidine kinase |  |
| 1. 2-keto-3-deoxygluconate kinase (pdb3KTN) | Ribokinase-like | PkfB carbohydrate kinase |  |
| 1. Hydroxyethyl thiazole kinase (pdb3HPD) | Ribokinase-like | hydroxyethyl thiazole kinase |  |
| 1. 1-phosphofructokinase (pdb2AJR) | Ribokinase-like | PkfB carbohydrate kinase. |  |
| 1. Tagatose-6-phosphate kinase (pdb2JG1) | Ribokinase-like | PkfB carbohydrate kinase. |  |
| ADP-dependent phosphofructokinase (pdb3DRW) | Ribokinase-like | ADP-specific Phosphofructokinase/Glucokinase. |  |
| ADP-dependent glucokinase (pdb1GC5) | Ribokinase-like | ADP-specific Phosphofructokinase/Glucokinase |  |
| Phosphomethyl pyrimidine kinase (pdb1UB0) | Ribokinase-like | ADP-specific Phosphofructokinase/Glucokinase |  |

^1^ pfam clan classification in brackets

^2^ pfam family/domain classification in brackets

^3^ No Detectable similarity to conventional kinases

Where there are 2 or domains recognised, these are denoted by ‘a’, ‘b’, etc. One domain has been selected to position the protein within the table.

**4 3**

**↓ ↓**


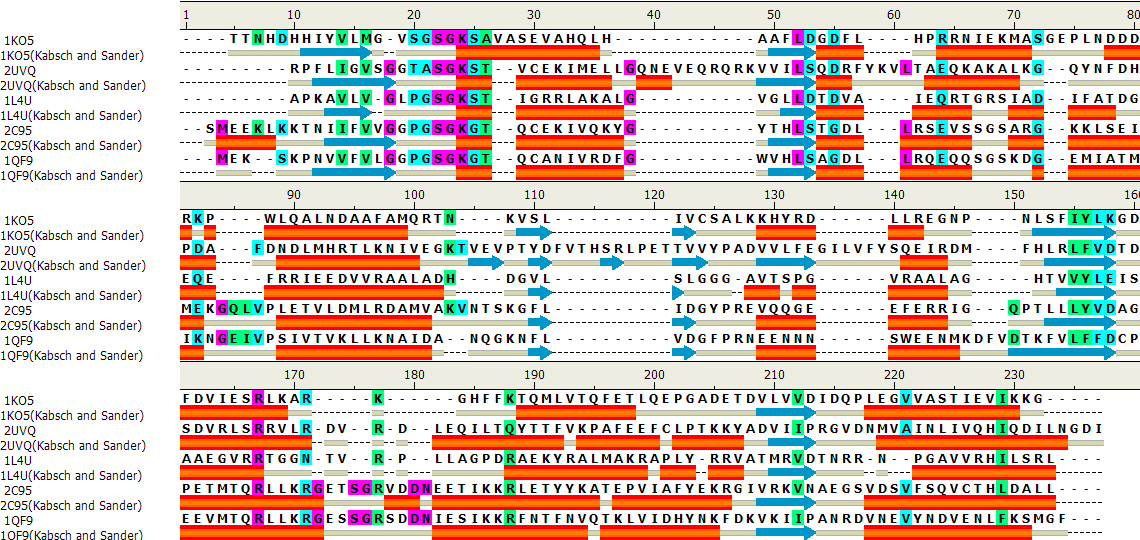


**1 2**

**↓ ↓**

**Figure SEQ2A.** The **Group 2** kinases belonging to the ‘P-loop containing nucleodside triphosphate hydrolase’ superfamily comprising the ‘shikimate kinase’, ‘phosphoribulokinase/uridine kinase’ and ‘adenylate kinase’ families which contain two conserved Arg residues in the active site linked to the proton translocation (Mechanism 2A, Table 2B). Pink = 100% identical, Green = >75% identical, turquoise = >50% identical. The indicated numbering is as per the identified residues as outlined in Sup Inf Table SI 1A. Secondary structure elements; Orange/Pink tube = Helix, Blue Arrow = Sheet, Grey = Coil. The indicated numbering is as per the identified residues as outlined in Sup Inf Table SI 1A. 1KO5, gluconate kinase; 2UVQ, uridine kinase; 1L4U, shikimate kinase, 2C95, adenylate kinase; 1QF9, cytidylate kinase.


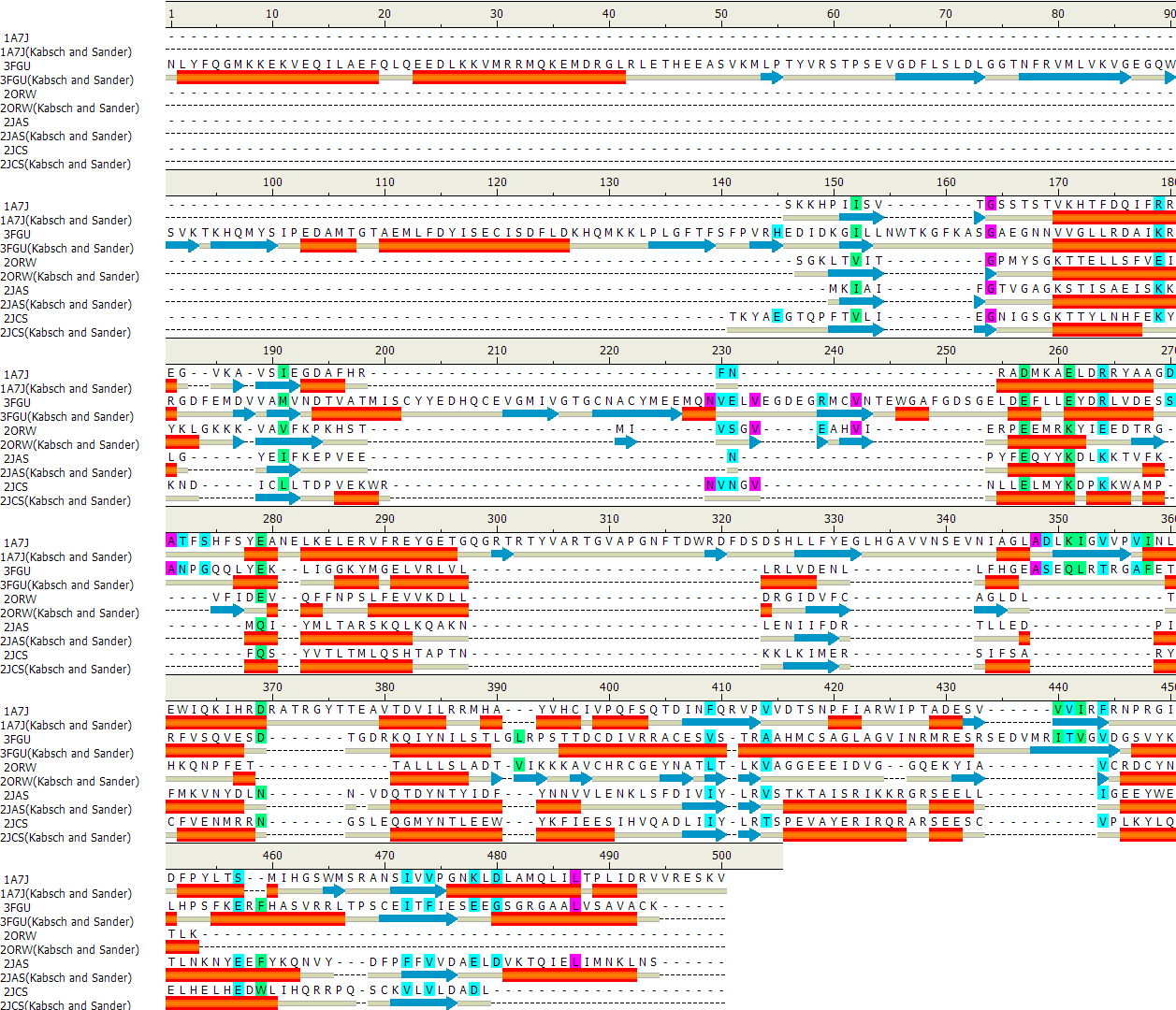


**↓**

**2**

**2 4**

**↓ ↓**

**1**

**Figure SEQ2B.** The **Group 2** kinases belonging to the ‘P-loop containing nucleoside triphosphate hydrolase’ superfamily comprising phosphoribulokinase /uridine kinase’ and ‘deoxynucleoside kinase’ families which contain a single conserved Arg residue in the active site linked to the proton translocation (Mechanism 2B, Table 2B). The indicated numbering is as per the identified residues as outlined in Sup Inf Table SI 2A. Pink = 100% identical, Green = >75% identical, turquoise = >50% identical. The indicated numbering is as per the identified residues as outlined in Sup Inf Table SI 1A. Secondary structure elements; Orange/Pink tube = Helix, Blue Arrow = Sheet, Grey = Coil. The indicated numbering is as per the identified residues as outlined in Sup Inf Table SI 1A. 1A7J; phosphoribulokinase; 2ORW, thymidine kinase; 2JAS, deoxyguanisine kinase; 2JCS, deoxyribonucleoside kinase; 3FGU, glucokinase.

**2**


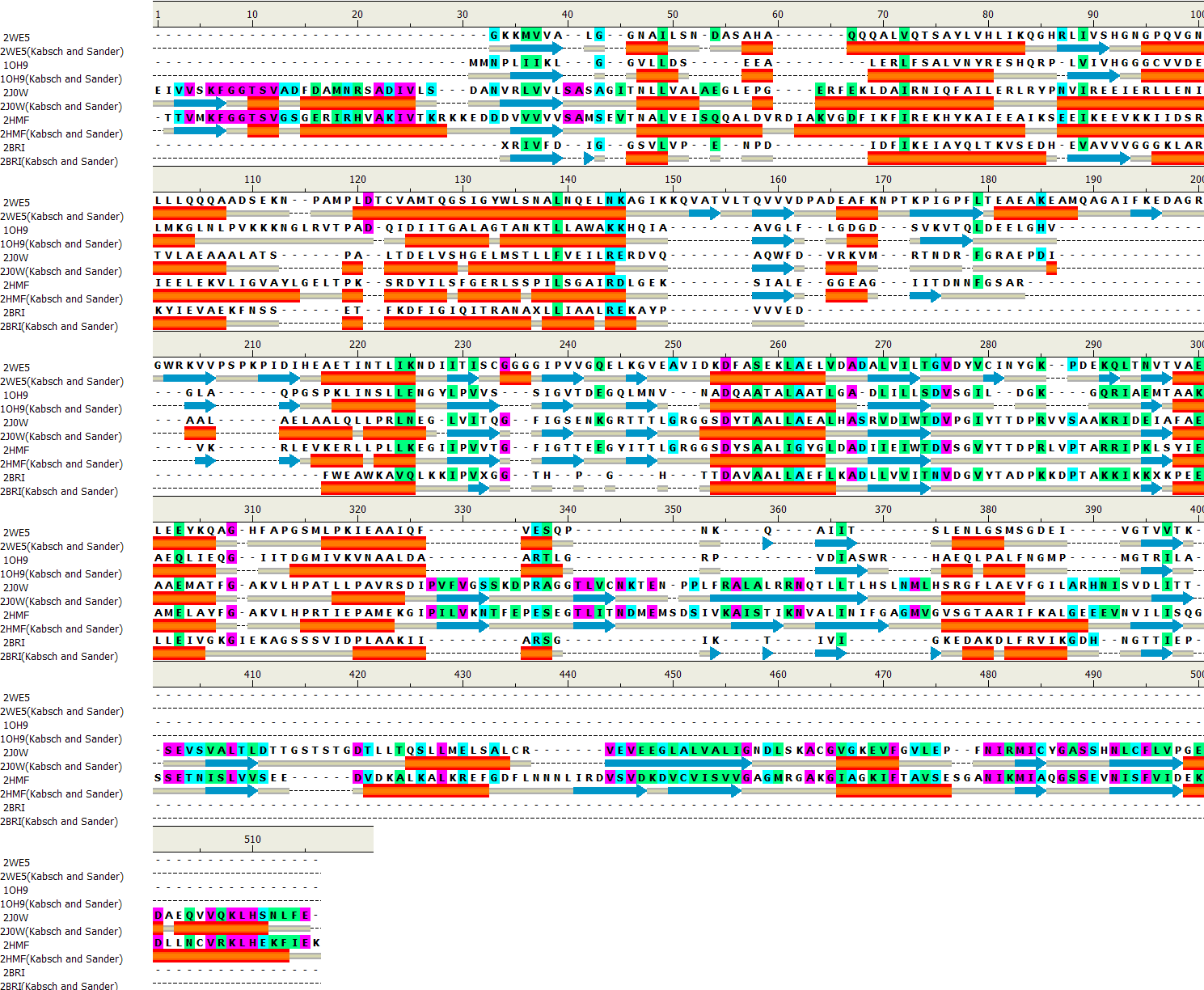


**↓**

**4**

**↓**

**2 4 2**

**↓ ↓ ↓**

**2**

**Figure SEQ2C.** A sequence alignment of the proteins making up the Group 2 aspartokinase family. The aspartokinase enzymes and uridylate enzymes contain an additional Arg residue facilitating phosphoryl transfer. Pink = 100% identical, Green = >75% identical, turquoise = >50% identical. The indicated numbering is as per the identified residues as outlined in Sup Inf Table SI 1A. Secondary structure elements; Orange/Pink tube = Helix, Blue Arrow = Sheet, Grey = Coil. The indicated numbering is as per the identified residues as outlined in Sup Inf Table SI 2A. 1WE5, carbamate kinase; 1OH9, acetylglutamate kinase; 2J0W, aspartokinase; 2BRI, uridylate kinase.


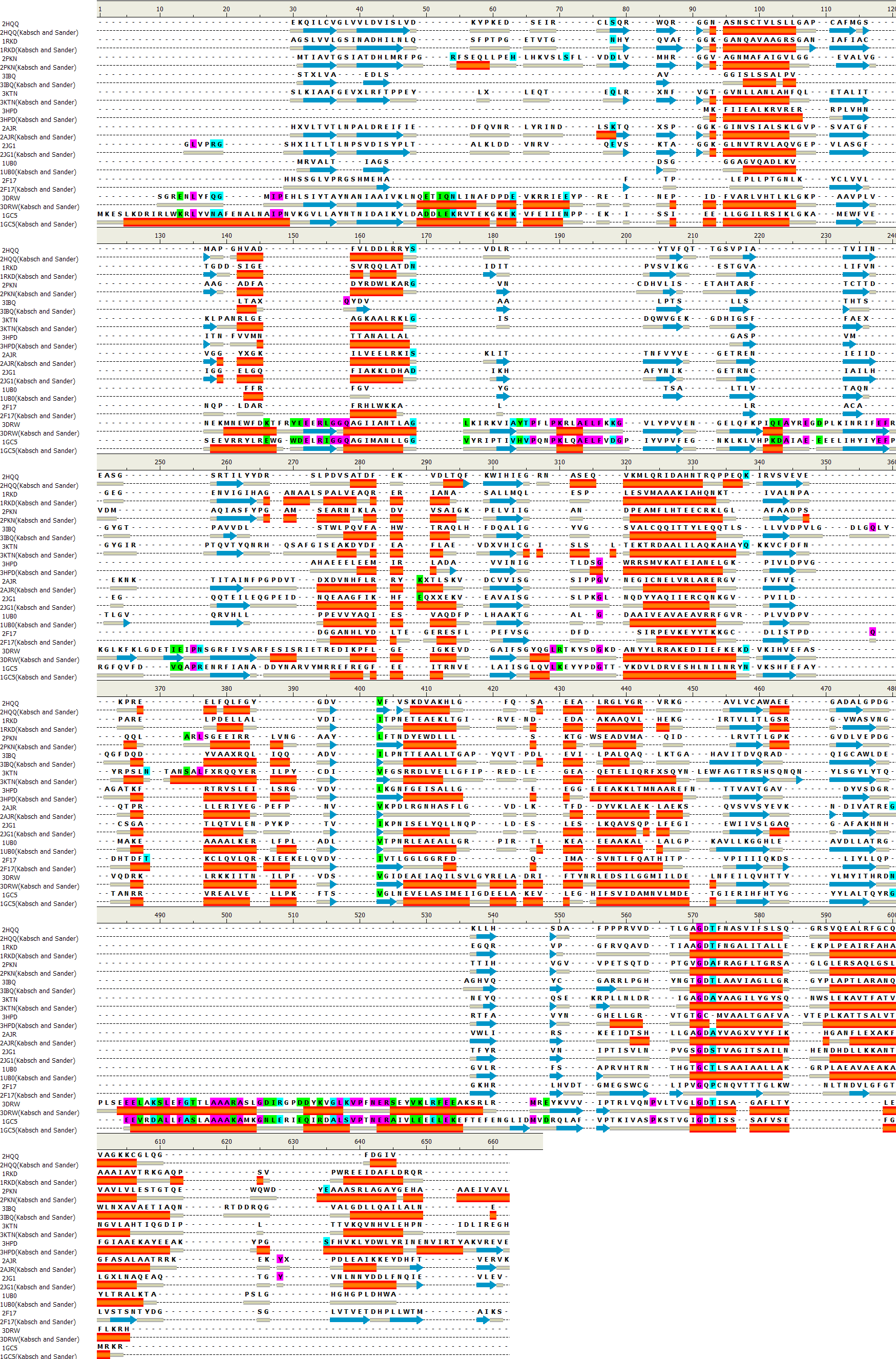


**13**


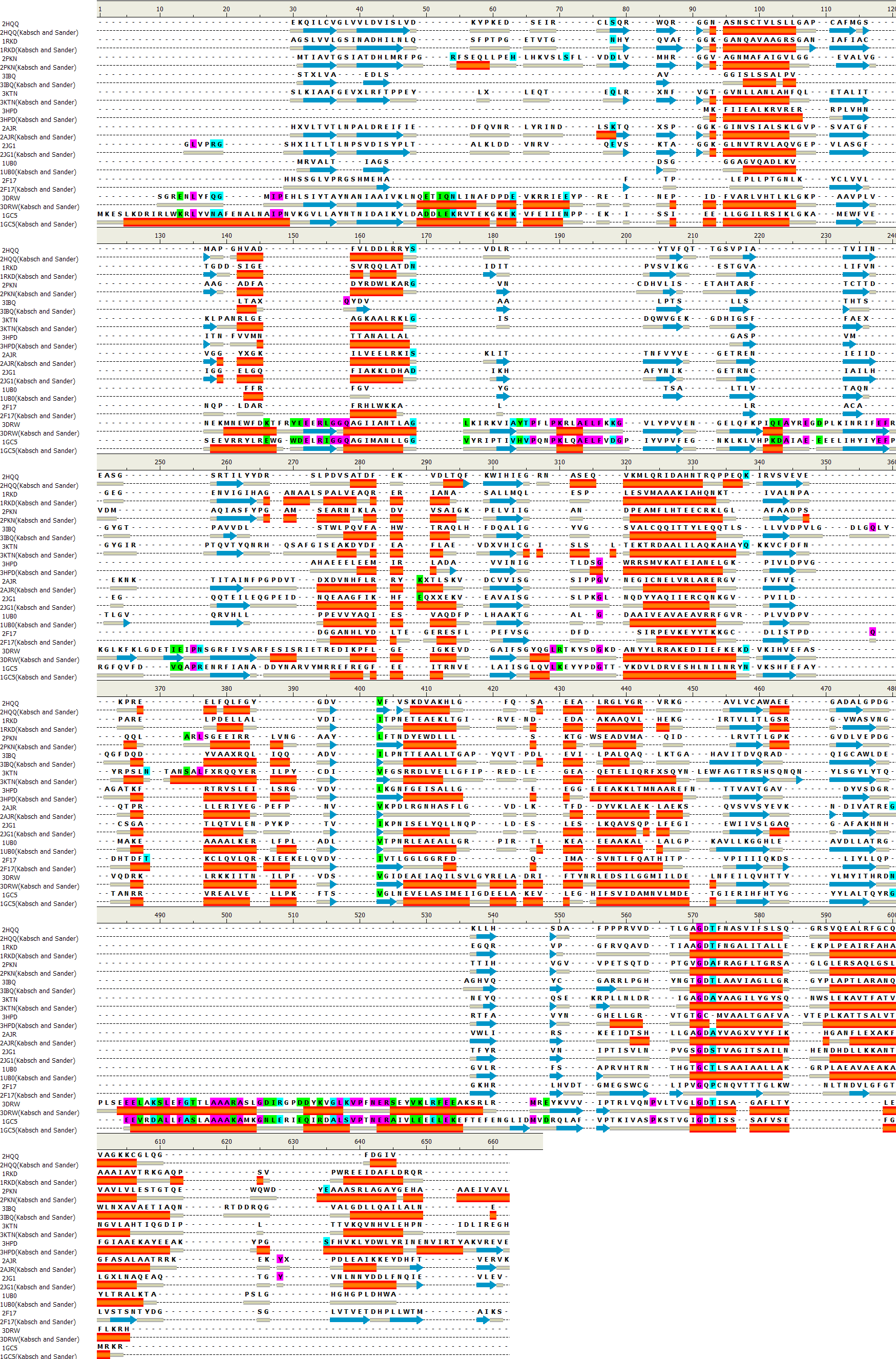


**1**

**1**

**1**

**13**

**1**

**3**

**2**


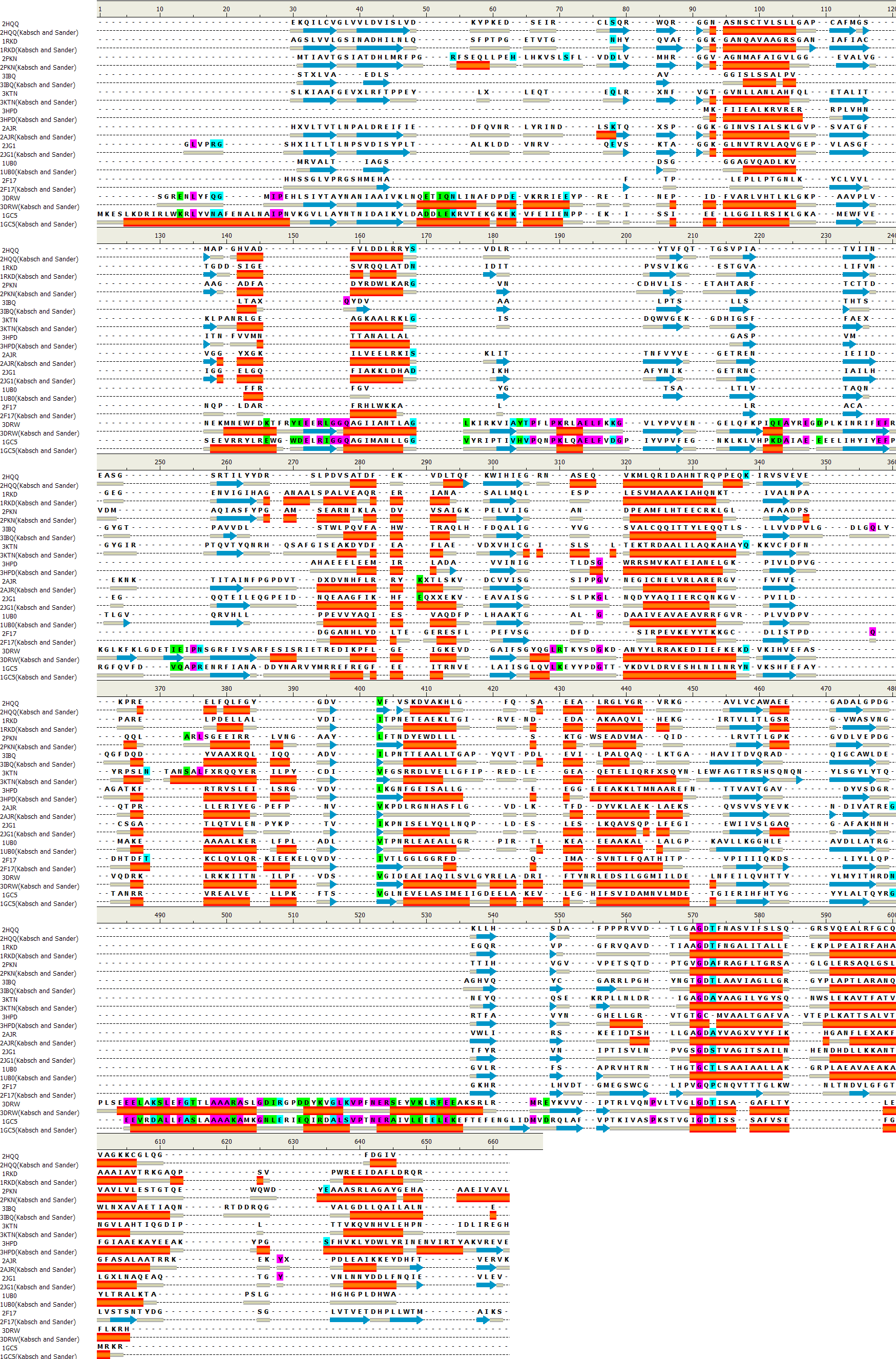


**3**

**2**

**2**

**Figure SEQ2D.** The conserved Thr and Asp residues responsible for the initiation of phosphoryl transfer within the ribokinase-like superfamily of the **Group 2** kinases. Pink = 100% identical, Green = >75% identical, turquoise = >50% identical. The indicated numbering is as per the identified residues as outlined in Sup Inf Table SI 2A. Secondary structure elements; Orange/Pink tube = Helix, Blue Arrow = Sheet, Grey = Coil. 3HQQ, ketohexokinase; 1RKD, ribokinase; 2PKN, adenosine kinase; 3IBQ, pyridoxal kinase; 3KTN, 2-keto-3-deoxygluconate kinase; 3HPD, Hydroxyethyl thiazole kinase; 2AJR, 1-phosphofructokinase; 2JG1, tagatose-6-phosphate kinase; 3IBQ, Pyridoxal kinase; 3DRW, ADP-dependent phosphofructokinase; 1GC5, ADP-dependent glucokinase; 1UB0, phosphomethyl pyrimidine kinase; 2F17, Thiamin pyrophosphate kinase.


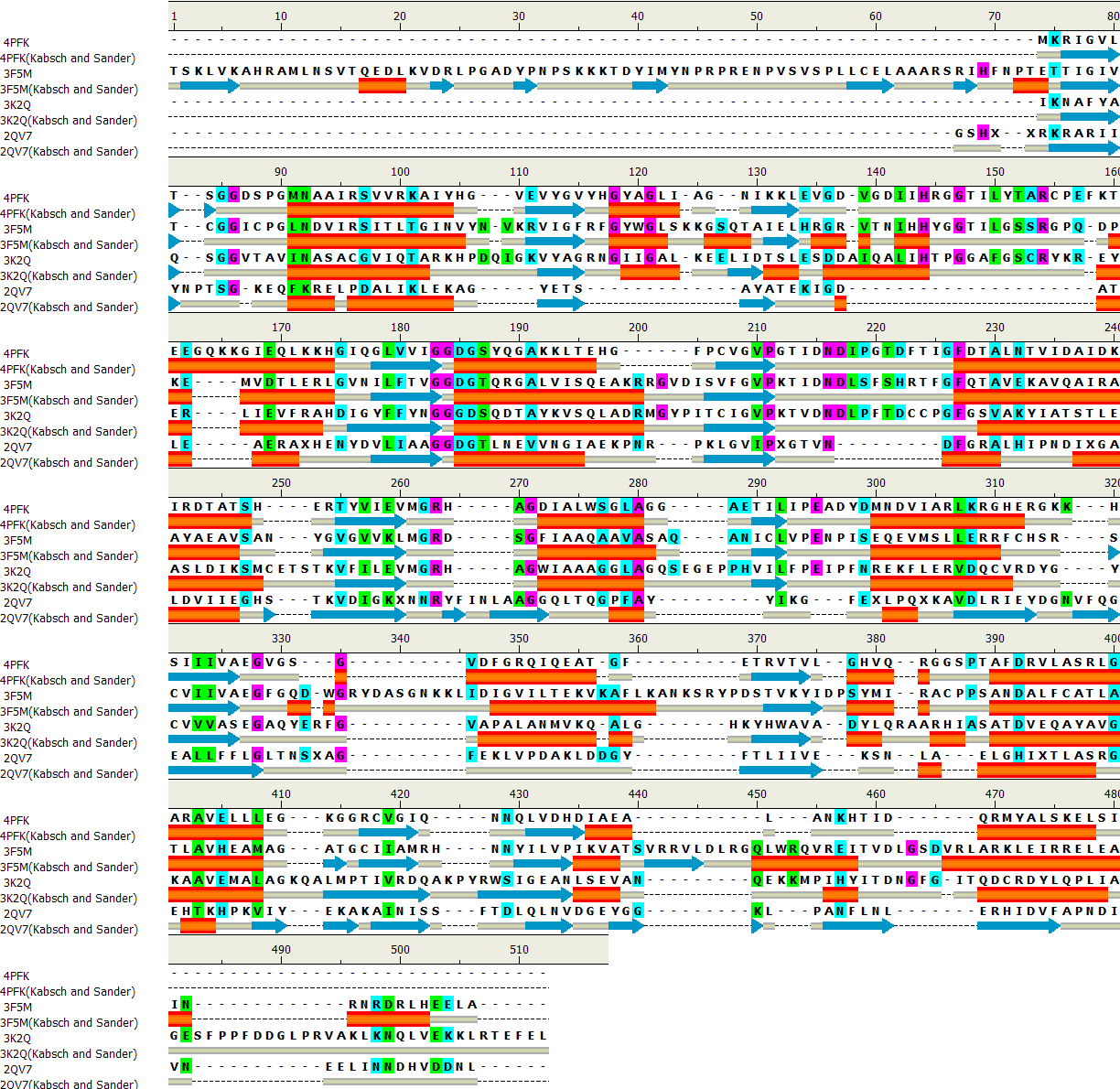


**3**

**1**

**2**

**1**

**Figure SEQ2E.** The conserved Arg and Lys residues responsible for the initiation of phosphoryl transfer within the PFK-like superfamily of the **Group 2** kinases. The indicated numbering is as per the identified residues as outlined in Sup Inf Table SI 2A. Secondary structure elements; Orange/Pink tube = Helix, Blue Arrow = Sheet, Grey = Coil. 4PFK, 6-Phosphofructokinase; 3F5M , 6-Phosphofructokinase; 2AJR, 1-Phosphofructokinase; 3K2Q , Diphosphate-fructose-6-phosphate 1-phosphotransferase; 2QV7, Diacylglycerol kinase.


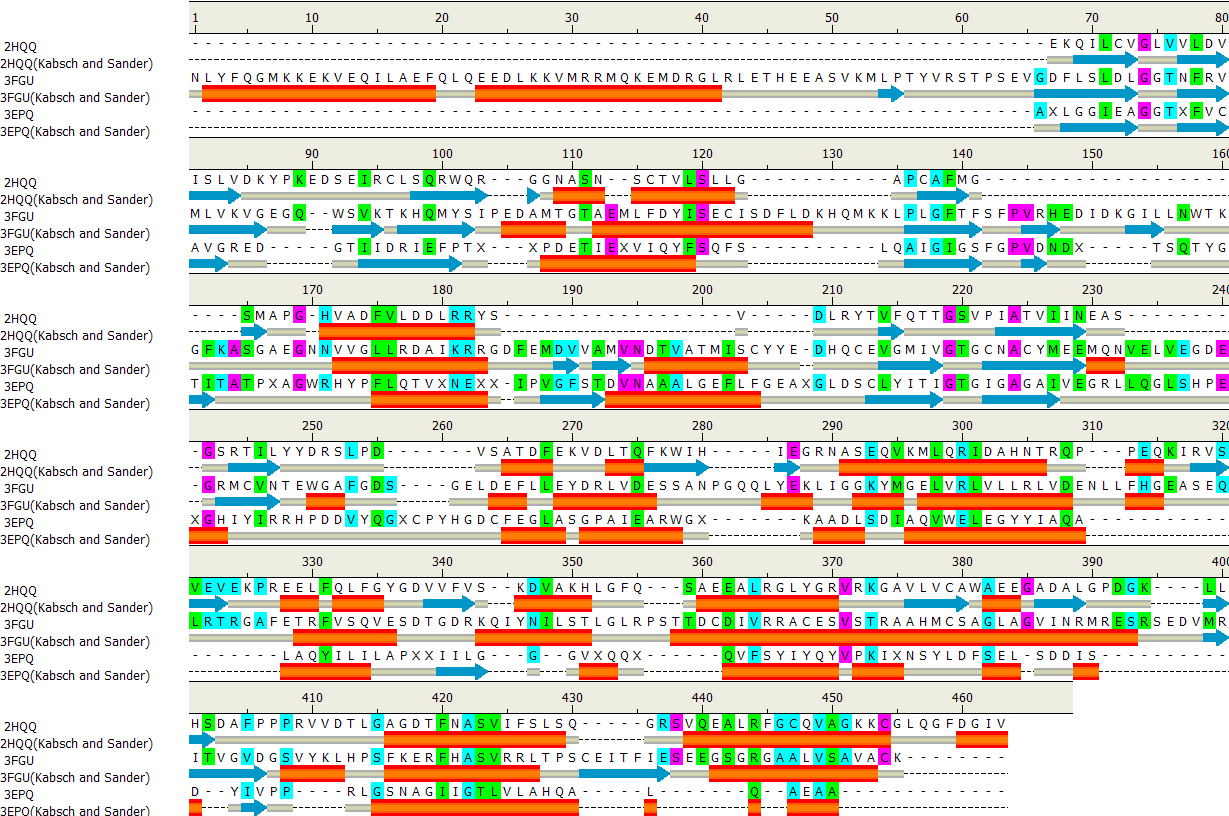


**2**

**1**

**Figure SEQ2F.** The conserved Thr and Asp residues responsible for the initiation of phosphoryl transfer within the Actin-like ATPase superfamily of the **Group 2** kinases. 3FGU, Hexokinase; 3HQQ, Ketohexokinase, 3EPQ, 4-Fructokinase.

**Table AF 3A.** Kinases representing **Group 3** and the identified conserved amino acid residues associated with the catalysis of phosphoryl transfer and the measured interatomic distances are shown. The PDB or uniprot accession numbers are indicated. Conserved residues replaced by conserved functionality are indicated by 3-letter code.

| **Kinase** | | **C-C=O to Adn-NH_2_** | **N7 to Arg^1^** | **C8-H to Arg^1^** | **Arg^1^ to α-PO_4_** | **Arg^1^ Nε to α-PO_4_** | **C8-H to α-PO_4_** | **C8-H to Asp^2^** | **Arg^3^-NH_3_ to β-PO_4_** | **C8-H to Arg^3^** | **Arg^3^-NH_3_ to β -PO_4_** | **Arg^4^-NH_3_ to α-PO_4_^-^** | **Arg^5^ PO_4_^-^** |
| --- | --- | --- | --- | --- | --- | --- | --- | --- | --- | --- | --- | --- | --- |
|  | **Ferredoxin-like fold kinases** | | | | | | | | | | | | |
| Nucleoside bis-phosphate kinase 2BEF ([*Dictyostelium discoideum*)](http://www.rcsb.org/pdb/search/smartSubquery.do?smartSearchSubtype=TreeEntityQuery&t=1&n=44689) | | NC | Arg62 2.883 | Arg62 2.725 | NC | NC | 3.249 | His59 2.950 | Thr98 3.853 | Arg109 | Arg109 |  |  |
| **HPPK** | |  |  |  |  |  |  |  |  |  |  |  |  |
| 2-amino-4-hydroxy-6-hydroxymethyldihydropteridine pyrophosphokinase 1EQ0 ([*Escherichia coli*)](http://www.rcsb.org/pdb/search/smartSubquery.do?smartSearchSubtype=TreeEntityQuery&t=1&n=562) | |  |  |  |  |  |  |  |  |  |  |  |  |
|  | **Guanido kinases** | | | | | | | | | | | | |
| Guanidoacetate kinase^6^ P51546 9 (*Drosophila melanogaster*) | |  |  | Arg287 | Arg287 | Arg287 |  | Asp330 | Arg125 | Arg125 | Arg127 | Arg315 | Arg231 |
| Creatine kinase 2GL6 ([*Homo sapiens*)](http://www.rcsb.org/pdb/search/smartSubquery.do?smartSearchSubtype=TreeEntityQuery&t=1&n=9606) | | Gly328 3.068 | Arg326 2.413 | Arg326 2.413 | Arg326 1.666 | Arg326 2.338 | 3.689 | Asp369 2.977 | Arg164 1.847 | Arg164 3.384 | Arg166 1.635 | Arg354 1.952 | Arg270 |
| Arginine kinase 1BG0 ([*Limulus polyphemus*)](http://www.rcsb.org/pdb/search/smartSubquery.do?smartSearchSubtype=TreeEntityQuery&t=1&n=6850)  Atlantic horseshoe crab | | Ser122 2.233 | Arg280 1.898 | Arg280 1.898 | Arg280 3.382 | Arg280 1.935 | 4.574 | Asp324 2.900 | Arg124 1.867 | Arg124 2.864 | Arg126 1.812 | Arg309 1.843 | Arg229 |
| Lombricine kinase^6^ O15991 (*Eisenia foetida*) red worm | |  |  | Arg281 | Arg281 | Arg281 |  | Asp323 | Arg118 | Arg118 | Arg120 | Arg308 | Arg324 |
|  | **Histidine kinase** | | | | | | | | | | | | |
| Histidine kinase | |  |  |  |  |  |  |  |  |  |  |  |  |
| [Pyruvate dehydrogenase (lipoamine)] kinase^6^ Q9SBJ1(*Arabidopsis thaliana*) | |  |  | Arg285 | Arg285 | Arg285 |  |  |  |  |  |  |  |
| [3-Methyl-2-oxobutanoate dehydrogenase (lipoamide)] kinase | |  |  |  |  |  |  |  |  |  |  |  |  |
| Polyphosphate kinase 1XDP  From Group 9 ([*Escherichia coli*)](http://www.rcsb.org/pdb/search/smartSubquery.do?smartSearchSubtype=TreeEntityQuery&t=1&n=562) | | Asp587 3.287 | Arg564 3.213 | Arg564 2.614 | NC | NC | 7.406 | NC | Arg375 3.353 |  | Arg405 2.994 |  | Arg594 3.281 |
| **Mean** | | **2.651** | **2.156** | **2.156** | **2.524** | **2.137** | **4.132** | **2.939** | **1.857** | **3.124** | **1.724** | **1.898** |  |
| **Standard Deviation** | | **0.590** | **0.364** | **0.364** | **1.213** | **0.285** | **0.626** | **0.054** | **0.014** | **0.368** | **0.125** | **0.077** |  |
| **% Standard Deviation** | | **22.276** | **16.894** | **16.894** | **48.074** | **13.338** | **15.147** | **1.853** | **0.762** | **11.770** | **7.262** | **4.062** |  |

1. Arg coordinated to C8-H, α-PO_4_, and the ArgNεH to β-PO_4_.

2. Coordinated Asp residue responsible for the protonation C8 during the rehybridization change from sp2 to sp3.

3,4. ATP phosphate backbone stabilization.

5. Arg responsible for substrate deprotonation and γ=PO_4_ protonation.

6. Residues identified by sequence alignment using UniProt sequence (Accession Number as indicated).

NC = No Coordinating residue

IS = Incomplete Structure.

**Figure MECH6.** Phosphoryl transfer mechanism found in the Group 3 kinases (guanido family comprising creatine and arginine kinase). This occurs via coordination of the adenyl C6-NH_2_ and protonation of C8 via a coordinated Asp changing C8 from sp2 to sp3 hybridization, and alters the protonation of C8-H. The C8-H becomes more acidic, allowing for the protonation of the β-PO_4_, via a conserved Arg. There is a concomitant transfer of an H^+^ to the β-PO_4_, facilitating the formation of the pentavalent intermediate between the γ-PO_4_ and the substrate nucleophile. There is a simultaneous Arg-mediated deprotonation of the substrate-NH that allows for the nucleophilic attack by the substrate, creating the pentavalent intermediate and allowing phosphoryl transfer. The protonated Arg then transfers the proton to the γ-PO_4_ changing the coordination of the Mg^2+^ from being β-PO_4_ to γ-PO_4_ coordinated to being α-PO_4_ to β-PO_4_ coordinated. The H^+^ originally arising from the C8 is then transferred back to C8, allowing the electron density of the adenyl moiety to return to the “ground-state” distribution.

**Table AF 3B.** Group 3 kinases

|  | **SUPERFAMILY^1^** | **FAMILY / DOMAIN^2^** |
| --- | --- | --- |
| **Group 3 Kinases** | | |
| 1. Nucleoside bis-phosphate kinase (pdb2BEF) | nd^3^ | Nucleoside diphosphate kinase (PF00334) |
| 1. 2-amino-4-hydroxy-6-hydroxymethyldeihydopter-idine pyrophosphokinase (pdb1EQ0) | nd | 7,8-dihydro-6-hydroxymethylpterin-pyrophosphokinase (PF01288) |
| 1. Guanidoacetate kinase (uniprotP51546) | 1. nd 2. gamma-glutamylcysteine synthetase/glutamine synthetase (CL0286) | 1. ATP:guanido phosphotransferase, N-terminal domain (PF02807) 2. ATP:guanido phosphotransferase, C-terminal catalytic domain (PF00217) |
| 1. Creatine kinase (pdb2GL6) | 1. nd 2. gamma-glutamylcysteine synthetase/glutamine synthetase (CL0286) | 1. ATP:guanido phosphotransferase, N-terminal domain (PF02807) 2. ATP:guanido phosphotransferase, C-terminal catalytic domain (PF00217) |
| 1. Arginine kinase (pdb1BG0) | 1. nd 2. gamma-glutamylcysteine synthetase/glutamine synthetase (CL0286) | 1. ATP:guanido phosphotransferase, N-terminal domain (PF02807) 2. ATP:guanido phosphotransferase, C-terminal catalytic domain (PF00217) |
| 1. Lombricine kinase (uniprotO15991) | 1. nd 2. gamma-glutamylcysteine synthetase/glutamine synthetase (CL0286) | 1. ATP:guanido phosphotransferase, N-terminal domain (PF02807) 2. ATP:guanido phosphotransferase, C-terminal catalytic domain (PF00217) |
| 1. Pyruvate dehydrogenase kinase (uniprotQ9SBJ1) | 1. nd 2. His Kinase A (phospho-acceptor) domain (CL0025) | 1. Mitochondrial branched-chain alpha-ketoacid dehydrogenase kinase (PF10436) 2. Histidine kinase-, DNA gyrase B-, and HSP90-like ATPase (PF02518) |

^1^ pfam clan classification

^2^ pfam family/domain classification

^3^ No Detectable similarity to conventional kinases

Where there are 2 or domains recognised, these are denoted by ‘a’, ‘b’, etc. One domain has been selected to position the protein within the table.


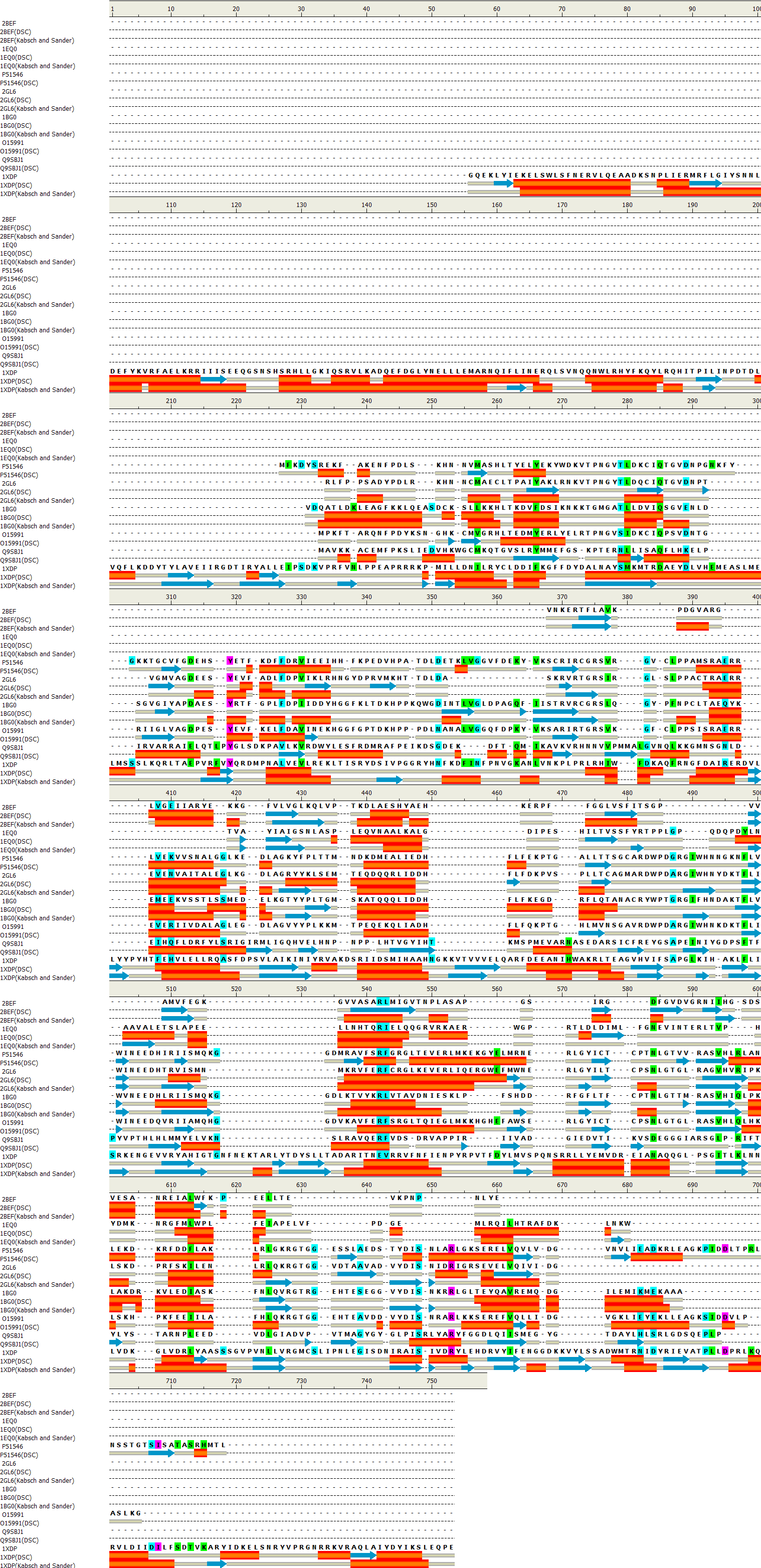


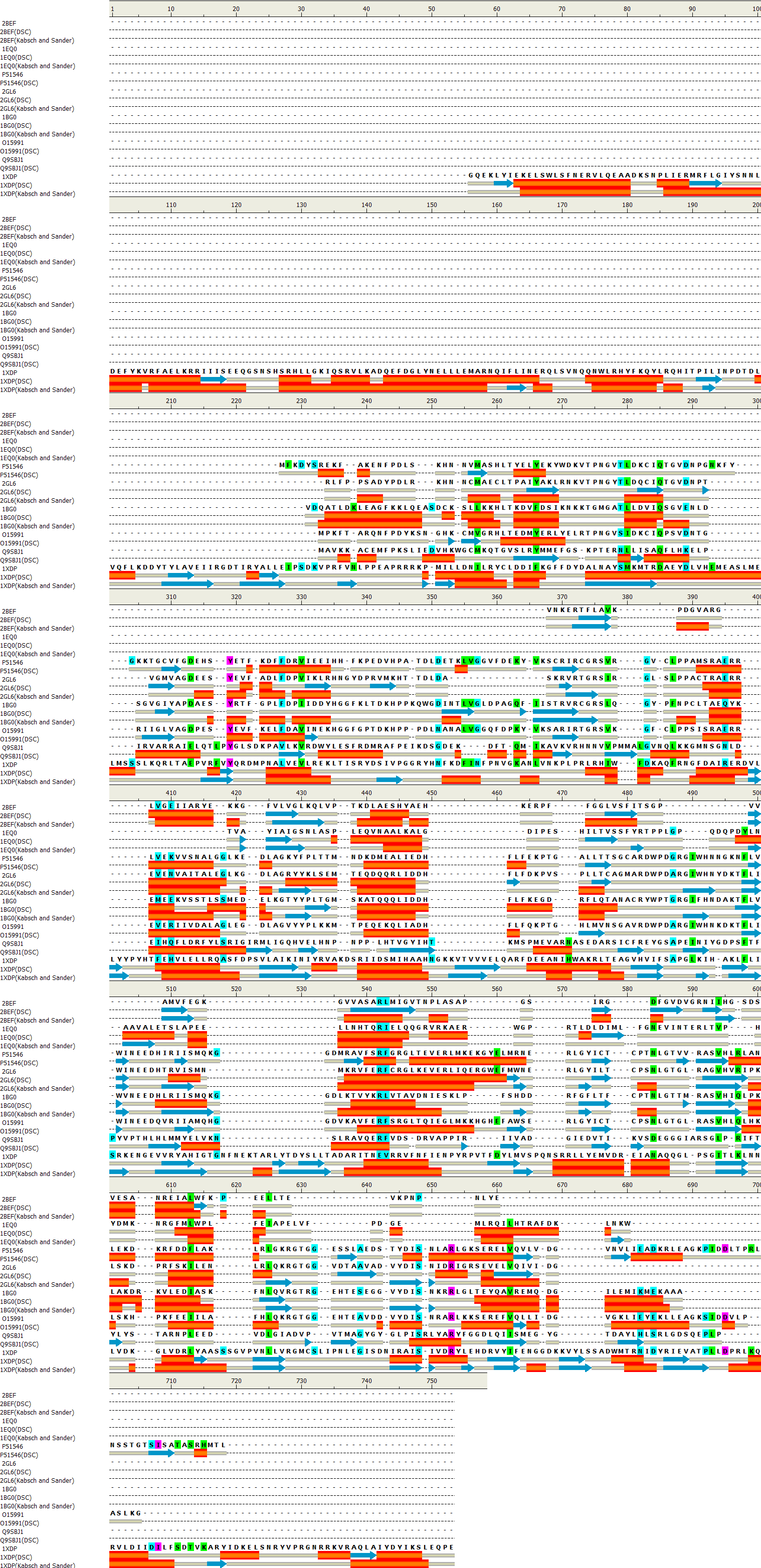


3

4


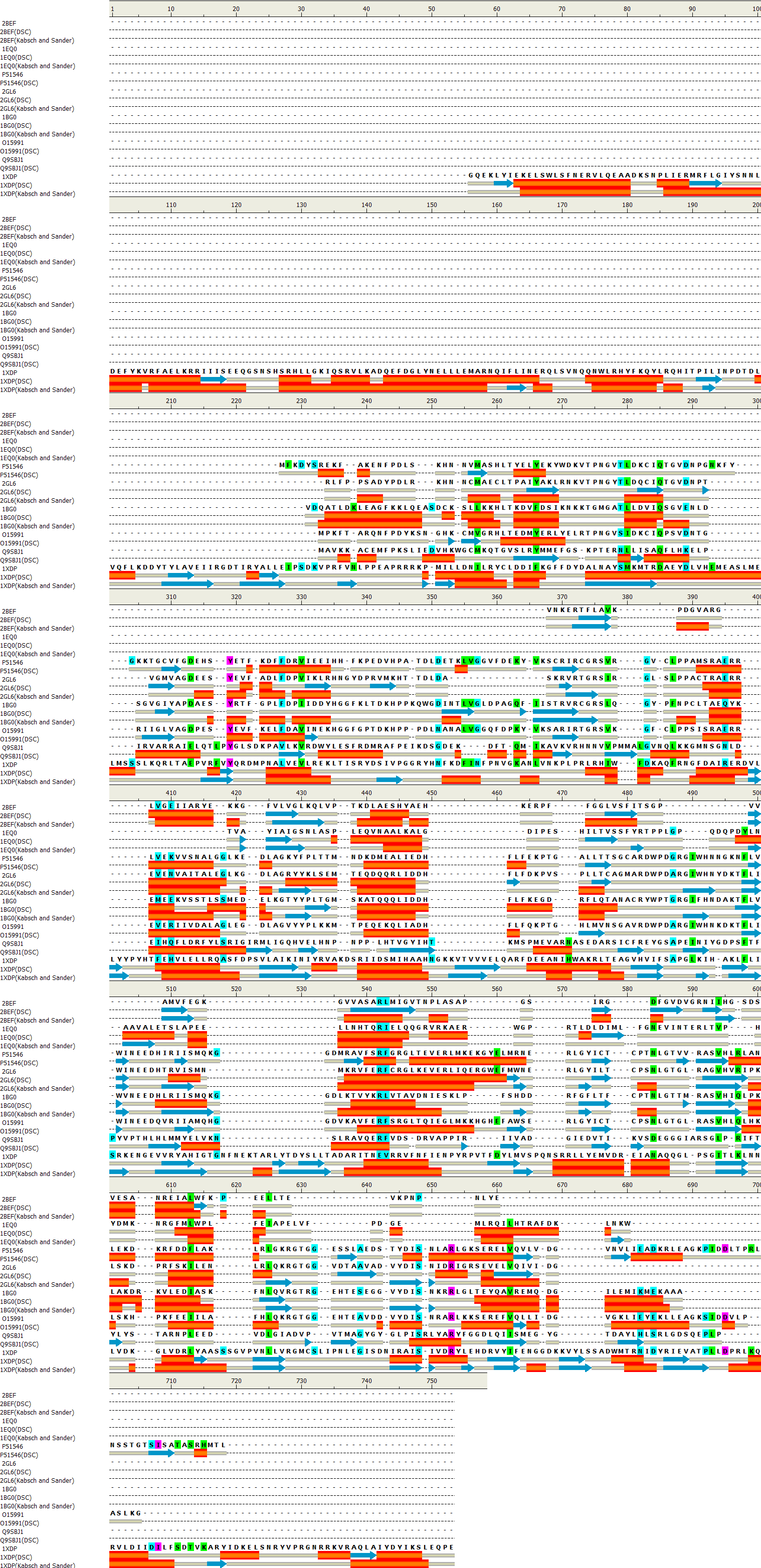


3

4

6

1

1

4

3


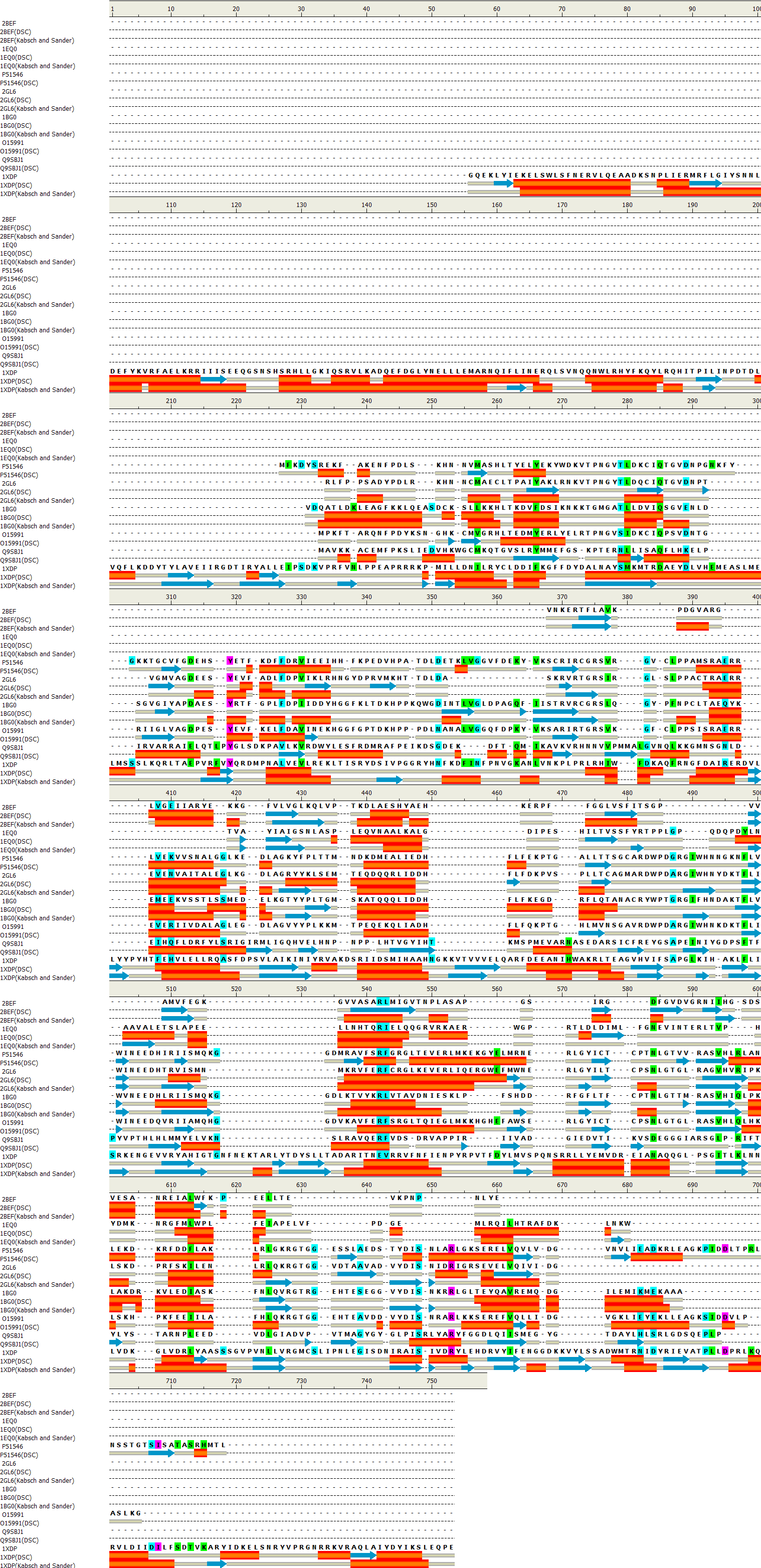


6

5

1

2

**Figure SEQ3**. A sequence alignment of the proteins making up the ‘ATP guanido’ kinase family within **Group 3** indicating the identified conserved amino acid residues responsible for initiating phosphoryl transfer. The indicated numbering is as per the identified residues as outlined in Sup Inf Table SI 3A. Pink = 100% identical, Green = >75% identical, turquoise = >50% identical. Secondary structure elements; Orange/Pink tube = Helix, Blue Arrow = Sheet, Grey = Coil. BEF 2, Nucleoside bis-phosphate kinase; 1EQ0, 2-amino-4-hydroxy-6-hydroxymethyldeihydopter-idine pyrophosphokinase; P51546, Guanidoacetate kinase; 2GL6, Creatine kinase; 1BG0, Arginine kinase; O15991, Lombricine kinase; Q9SBJ1, Pyruvate dehydrogenase (lopiamine) kinase; 1XDP, Polyphosphate kinase.

**Table AF 4A.** Kinases representing **Group 4** and the identified conserved amino acid residues associated with the catalysis of phosphoryl transfer and the measured inter-atomic distances are shown. The PDB or uniprot accession numbers are indicated. Conserved residues replaced by conserved functionality are indicated by 3-letter code.

| **Kinase** | **C8-H to α-PO_4_** | **Arg^1^** | **Glu^2^** | **Arg^3^** | **Arg^4^** | **Ser/Thr^5^** | **Ser/Thr^6^** | **Asp/Substrate^7^** | **Gly Loop^13^** |
| --- | --- | --- | --- | --- | --- | --- | --- | --- | --- |
| **Ribonuclease H-like sequences** | | | | | | | | | |
| Hexokinase^9,10^ 1DGK ([*Homo sapiens*)](http://www.rcsb.org/pdb/search/smartSubquery.do?smartSearchSubtype=TreeEntityQuery&t=1&n=9606) | 7.097 5.825^9^ | Lys785 αC=O 1.513 | NCR | NCR | NCR | Thr863^11^ 3.895 | Thr863^11^ 2.981 | NSub |  |
| Hexokinase^9,10^ 2E2P ([*Sulfolobus tokodaii*)](http://www.rcsb.org/pdb/search/smartSubquery.do?smartSearchSubtype=TreeEntityQuery&t=1&n=111955) | 7.097 5.825^9^ | Arg251 1.513 | NCR | NCR | NCR | Arg251^12^ 3.511 | Arg251^11^ 2.169 | NSub | Leu245-Arg251 Gly248 3.550 |
| Glucokinase^10^ 3FGU ([*Homo sapiens*)](http://www.rcsb.org/pdb/search/smartSubquery.do?smartSearchSubtype=TreeEntityQuery&t=1&n=9606) | 4.650 | Arg333 OA | Glu331 OA | Arg85^12^ 2.987 | Arg85^12^ 3.197 | Ser411^11^ 3.487 | Ser411^11^ 3.313 | Sugar/Asp205 2.041 | O-P 2.681 |
| Fructokinase 3LKI (*Xylella fastidiosa*) | 2.878 Direct | Val266 αC=O 3.870 |  | Lys203 | Lys203 2.144 |  | Thr238 3.268 |  | Arg178 2.333 |
| Rhamulokinase^10^ 2CGJ **(**[*Escherichia coli*)](http://www.rcsb.org/pdb/search/smartSubquery.do?smartSearchSubtype=TreeEntityQuery&t=1&n=469008) | 5.001 | Arg37 6.090 | Glu427 1.990 | Arg17 2.152 | Arg17 2.184 | Thr259 | Arg37^12^ 3.849 | Fructose/Asp237 1.813 | Ile399-Gln405 Gly402 2.525 |
| Mannokinase Q0BVN7 (*Granulibacter bethesdensis*) | NS |  |  |  |  |  |  |  |  |
| Gluconokinase^10^ 3LL3 ([*Lactobacillus acidophilus*)](http://www.rcsb.org/pdb/search/smartSubquery.do?smartSearchSubtype=TreeEntityQuery&t=1&n=1579) | 5.632 | Arg305 3.595 | Asp312 OAS | Lys15 OAS | Lys15 OAS | Thr259 | Thr259 4.116 | D-xylulose/Asp237 3.471 | Asn396-Leu402 Gly400 2.245 |
| L-ribulokinase 3JVP ([*Bacillus halodurans*)](http://www.rcsb.org/pdb/search/smartSubquery.do?smartSearchSubtype=TreeEntityQuery&t=1&n=86665) | No ADP | Lys452 | Asp455 | Arg18 | Arg18 | Thr296 | Thr296 | Asp274 |  |
| Xylulokinase^10^ 3HZ6 ([*Chromobacterium violaceum*)](http://www.rcsb.org/pdb/search/smartSubquery.do?smartSearchSubtype=TreeEntityQuery&t=1&n=536) | 5.068 | Arg412 4.004 N7 | Asp433 OAS | Lys16 OAS | Lys16 OAS | Thr263 6.618 | Thr263 3.450 | D-xylulose/Asp241 3.019 | Arg405-Ala411 Gly409 2.839 |
| Erythritol kinase Q92NH0 (*Rhizobium meliloti*) | NS | Arg421 | Glu441 | Lys15 | Lys15 | Ser264 | Ser264 | Asp242 |  |
| Glycerol kinase^10^ 1BWF **(**[*Escherichia coli*)](http://www.rcsb.org/pdb/search/smartSubquery.do?smartSearchSubtype=TreeEntityQuery&t=1&n=469008) | 5.856 | Arg436 3.951 | Asp325 4.589 | Arg17 2.229 | Arg17 3.978 | Arg436^12^ 3.951 | Arg436^12^ 2.033 | Glycerol/Asp245 2.755 | Arg417-Ala414 Gly411 3.189 |
| Pantothenate kinase^9,10^ 3BF1 ([*Thermotoga maritima*)](http://www.rcsb.org/pdb/search/smartSubquery.do?smartSearchSubtype=TreeEntityQuery&t=1&n=2336) | 6.169 6.070^9^ | His154 αC=O 3.116 | NCR | NCR | NCR | Thr10 3.921 | Thr10 2.507 | Pantothenate/Asp105 2.414 |  |
| D-ribulokinase Q8YBC1 (*Brucella melitensis*) | NS | Arg351 | Asp361 | Arg20 | Arg20 | Thr286 | Thr286 | Asp258 |  |
| L-fuculokinase P11553 **(**[*Escherichia coli*)](http://www.rcsb.org/pdb/search/smartSubquery.do?smartSearchSubtype=TreeEntityQuery&t=1&n=469008) | NS | Arg322 | Glu329 | Lys27 | Lys27 | Thr273 | Thr273 |  |  |
| L-xylulokinase B7MFE7 **(**[*Escherichia coli*)](http://www.rcsb.org/pdb/search/smartSubquery.do?smartSearchSubtype=TreeEntityQuery&t=1&n=469008) | NS | His405 | Asp415 | Lys16 | Lys16 | Thr265 | Thr265 | Asp243 |  |
| Allose kinase 3HTV **(**[*Escherichia coli*)](http://www.rcsb.org/pdb/search/smartSubquery.do?smartSearchSubtype=TreeEntityQuery&t=1&n=469008) | No ADP |  |  |  |  |  |  |  |  |
| 2-dehydro -3-deoxygaltonokinase | NS |  |  |  |  |  |  |  |  |
| Acetyl-glucosamine kinase 2CH6 ([*Homo sapiens*)](http://www.rcsb.org/pdb/search/smartSubquery.do?smartSearchSubtype=TreeEntityQuery&t=1&n=9606) | 4.382 | Arg218 2.364 | Glu222 OA | Arg14 3.654 | Arg14 3.973 | Ser27111 3.081 | Ser27111 3.022 | GlcNAc/Asp107 2.220 | Leu267-Trp273 Ser271 3.554 |
| Acetyl-mannosamine kinase 3EO3 ([*Homo sapiens*)](http://www.rcsb.org/pdb/search/smartSubquery.do?smartSearchSubtype=TreeEntityQuery&t=1&n=9606) | No ADP |  |  |  |  |  |  |  |  |
| Polyphosphate-glucose phosphotransferase 1WOQ ([Arthrobacter sp.](http://www.rcsb.org/pdb/search/smartSubquery.do?smartSearchSubtype=TreeEntityQuery&t=1&n=184230)) |  |  |  |  |  |  |  | Polyphosphate substrate |  |
| Beta-glucoside kinase Q926Y3 (*Listeria innocua*) | NS |  |  |  |  |  |  |  |  |
| Acetate kinase9 1TUY ([*Methanosarcina thermophila*)](http://www.rcsb.org/pdb/search/smartSubquery.do?smartSearchSubtype=TreeEntityQuery&t=1&n=2210) | 4.487 | Arg285 3.156 | Asp286 |  |  | Asn355 2.673 |  | Glu384 Arg241 His208 His180 | Ala330-Ser336 Gly331 2.161 |
| Butyrate kinase βPO4 1SAZ ([*Thermotoga maritima*)](http://www.rcsb.org/pdb/search/smartSubquery.do?smartSearchSubtype=TreeEntityQuery&t=1&n=2336) |  | Arg257 2.907 |  |  |  | His307 |  | Glu213 Arg214 His154 His182 | Val300-Ala306 Gly304 2.946 |
| Branched-chain-fatty-acid kinase Q3ETP1 (*Bacillus thuringiensis*) | NS |  |  |  |  |  |  |  | Ile322-Gly328 Gly326 2.566 |
| Propionate kinase 1X3N ([*Salmonella enterica* subsp*. enterica*)](http://www.rcsb.org/pdb/search/smartSubquery.do?smartSearchSubtype=TreeEntityQuery&t=1&n=90371) | 4.512 | Arg280 3.866 |  |  |  | Asn330 |  | Asp143 Arg236 His175 His203 |  |
|  |  |  |  |  |  |  |  |  |  |
| **Mean** | **4.932** | **3.155** |  | **2.756** | **3.095** | **3.495** | **2.672** | **2.533** | **2.781** |
| **Standard Deviation** | **0.876** | **0.815** |  | **0.708** | **0.908** | **0.446** | **0.537** | **0.583** | **0.488** |
| **% Standard Deviation** | **12.535** | **26.847** |  | **25.681** | **25.484** | **37.689** | **19.980** | **23.006** | **15.881** |

1. N7 coordinated.
2. Stabilization of Arg^1^ interatomic distance.
3. α-PO_4_ coordinated Arg.
4. β-PO_4_ coordinated Arg.
5. C8-H coordinated.
6. β-PO_4_ coordinated.
7. Sugar-OH coordinated Asp.
8. γ-PO_4_ coordinated.
9. C8-H β-PO_4_ coordinated.
10. Optimization of the pyrimidine/β-d-sugar torsion angle.
11. Optimization of the Thr/Ser-OH torsion angle.
12. Optimization of the Arg torsion angles, not included in the interatomic distance calculation.
13. Loop containing conserved Gly responsible for the responsible for the stabilization of the carbene.

OAS = Active site partially open.

OA = orientated away from imidazole moiety of ATP.

NCR = no coordinating residue.

NSub = no substrate for phosphorylation in structure.

**Figure MECH7.** Phosphoryl transfer mechanism found in the Group 4 kinases (hexokinase family with polyol substrate). This occurs via coordination of an arginine residue to the N7/C8 of the imidazole moiety mediating the change in C8 hybridization from sp2 to sp3, and altering the protonation of N7 and C8. Protonation of the N7 occurs via a conserved the Arg residue with the NH_2_ being coordinated directly to N7, with an interatomic distance of 3.065 ± 0.823 Å. The Arg residue in Group 4 kinases is always stabilized by an associated Asp/Glu residue. The reaction occurs via a carbene mechanism with the carbene being stabilized via the interaction of a conserved backbone carbonyl that is within bonding distance of C8, causing C8-H to become more acidic, allowing for the protonation of the α-PO_4_, via a conserved Ser/Thr. There is a concomitant transfer of an H^+^ from the α-PO_4_ to the β-PO_4_ via a conserved Arg, thereby facilitating the formation of the pentavalent intermediate between the γ-PO_4_ and the substrate nucleophile. There is a concomitant Asp-mediated deprotonation of the substrate –OH, allowing for the nucleophilic attack by the substrate. This creates the pentavalent intermediate and allows phosphoryl transfer. The protonated Asp then transfers the proton to the γ-PO_4_, changing the coordination of the Mg^2+^ from being β-PO_4_ to γ-PO_4_ coordinated to being α-PO_4_ to β-PO_4_ coordinated. The H^+^ originally arising from the C8 is then transferred back to C8, allowing the electron density of the adenyl moiety to return to the “ground-state” distribution.

**Table AF 4B.** Group 4 kinase

|  | **SUPERFAMILY^1^** | **FAMILY / DOMAIN^2^** |
| --- | --- | --- |
| **Group 4 Kinases** | | |
| 1. Hexokinase (pdb1DGK) | Actin-like ATPase (CL0108) | Hexokinase 1 (PF00349) and Hexokinase 2 (PF03727) |
| 1. Glucokinase (pdb3FGU) | Actin-like ATPase (CL0108) | Hexokinase 1 (PF00349) and Hexokinase 2 (PF03727) |
| 1. Fructokinase (pdb3LKI) | Ribokinase-like (CL0118) | pfkB family carbohydrate kinase (PF00294) |
| 1. Rhamulokinase (pdb2CGL) | Ribokinase-like (CL0118) | FGGY family of carbohydrate kinases, N-terminal domain (PF00370) and C-terminal domain (PF02782) |
| 1. Mannokinase (uniprotQ0BVN7) | Actin-like ATPase (CL0108) | ROK family (PF00480) |
| 1. Glucokinase (pdb3LL3) | Actin-like ATPase (CL0108) | FGGY family of carbohydrate kinases, N-terminal domain (PF00370) and C-terminal domain (PF02782) |
| 1. L-ribulokinase (pdb3JVP) | Actin-like ATPase (CL0108) | FGGY family of carbohydrate kinases, N-terminal domain (PF00370) and C-terminal domain (PF02782) |
| 1. Xylulokinase (pdb3HZ6) | Actin-like ATPase (CL0108) | FGGY family of carbohydrate kinases, N-terminal domain (PF00370) and C-terminal domain (PF02782) |
| 1. Erythritol kinase (uniprotQ92NH0) | Actin-like ATPase (CL0108) | FGGY family of carbohydrate kinases, N-terminal domain (PF00370) and C-terminal domain (PF02782) |
| 1. Glycerol kinase (pdb1BWF) | Actin-like ATPase (CL0108) | FGGY family of carbohydrate kinases, N-terminal domain (PF00370) and C-terminal domain (PF02782) |
| 1. Pantothenate kinase(pdb3BF1) | Actin-like ATPase (CL0108) | Bordetella pertussis Bvg accessory factor family (PF03309) |
| 1. D-ribulokinase (uniprotQ8YBC1) | Actin-like ATPase (CL0108) | FGGY family of carbohydrate kinases, N-terminal domain (PF00370) and C-terminal domain (PF02782) |
| 1. L-fuculokinase (uniprotP11553) | Actin-like ATPase (CL0108) | FGGY family of carbohydrate kinases, N-terminal domain (PF00370) and C-terminal domain (PF02782) |
| 1. L-xylulokinase (uniprotB7MFE7) | Actin-like ATPase (CL0108) | FGGY family of carbohydrate kinases, N-terminal domain (PF00370) and C-terminal domain (PF02782) |
| 1. Allose kinase (pdb3HTV) | Actin-like ATPase (CL0108) | ROK family (PF00480) |
| 1. 2-dehydro-3-deoxygalactonokinase (uniprotP31459) | nd^3^ | 2-keto-3-deoxy-galactonokinase (PF05035) |
| 1. Acetyl-glucosamine kinase (pdb2CH6) | Actin-like ATPase (CL0108) | BadF/BadG/BcrA/BcrD ATPase family (PF01869) |
| 1. Acetyl-mannosamine kinase (pdb3EO3) | Actin-like ATPase (CL0108) | ROK family (PF00480) |
| 1. Polyphosphate-glucoase phosphotransferase (pdb1WOQ) | Actin-like ATPase (CL0108) | ROK family (PF00480) |
| 1. Beta-glucoside kinase (uniprotQ926Y3) | Actin-like ATPase (CL0108) | ROK family (PF00480) |
| 1. Acetate kinase (pdb1TUY) | Actin-like ATPase (CL0108) | Acetokinase family (PF00871) |
| 1. Butyrate kinase (pdb1SAZ) | Actin-like ATPase (CL0108) | Acetokinase family (PF00871) |
| 1. Branched-chain fatty acid kinase (uniprotQ3ETP1) | Actin-like ATPase (CL0108) | Acetokinase family (PF00871) |
| 1. Propionate kinase (uniprotP11868) | Actin-like ATPase (CL0108) | Acetokinase family (PF00871) |

^1^ pfam clan classification in brackets

^2^ pfam family/domain classification in brackets

^3^ No Detectable similarity to conventional kinases

Where there are 2 or domains recognised, these are denoted by ‘a’, ‘b’, etc. One domain has been selected to position the protein within the table.


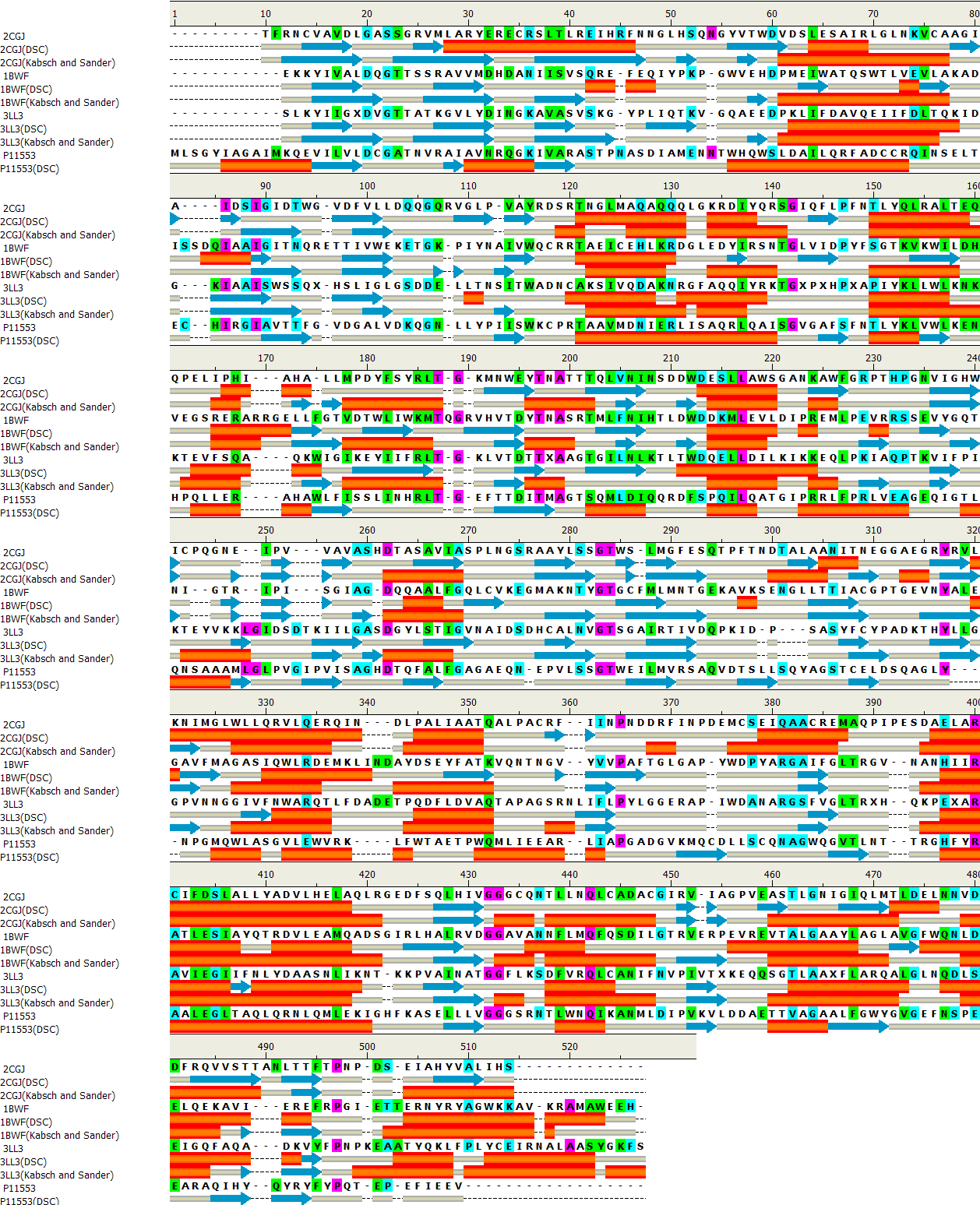


3

7

5

1

1

2

2

**Figure SEQ4A**. A sequence alignment of the proteins making up the **Group 4** ‘FGGY’ family, within the Type-A subfamily. The indicated numbering is as per the identified residues as outlined in Sup Inf Table SI 4A. Pink = 100% identical, Green = >75% identical, turquoise = >50% identical. Secondary structure elements; Orange/Pink tube = Helix, Blue Arrow = Sheet, Grey = Coil. 2CGJ, Rhamulokinase; 3LL3, Gluconokinase; 1BWF, Glycerol kinase; P11553, L-Fuculokinase.

**
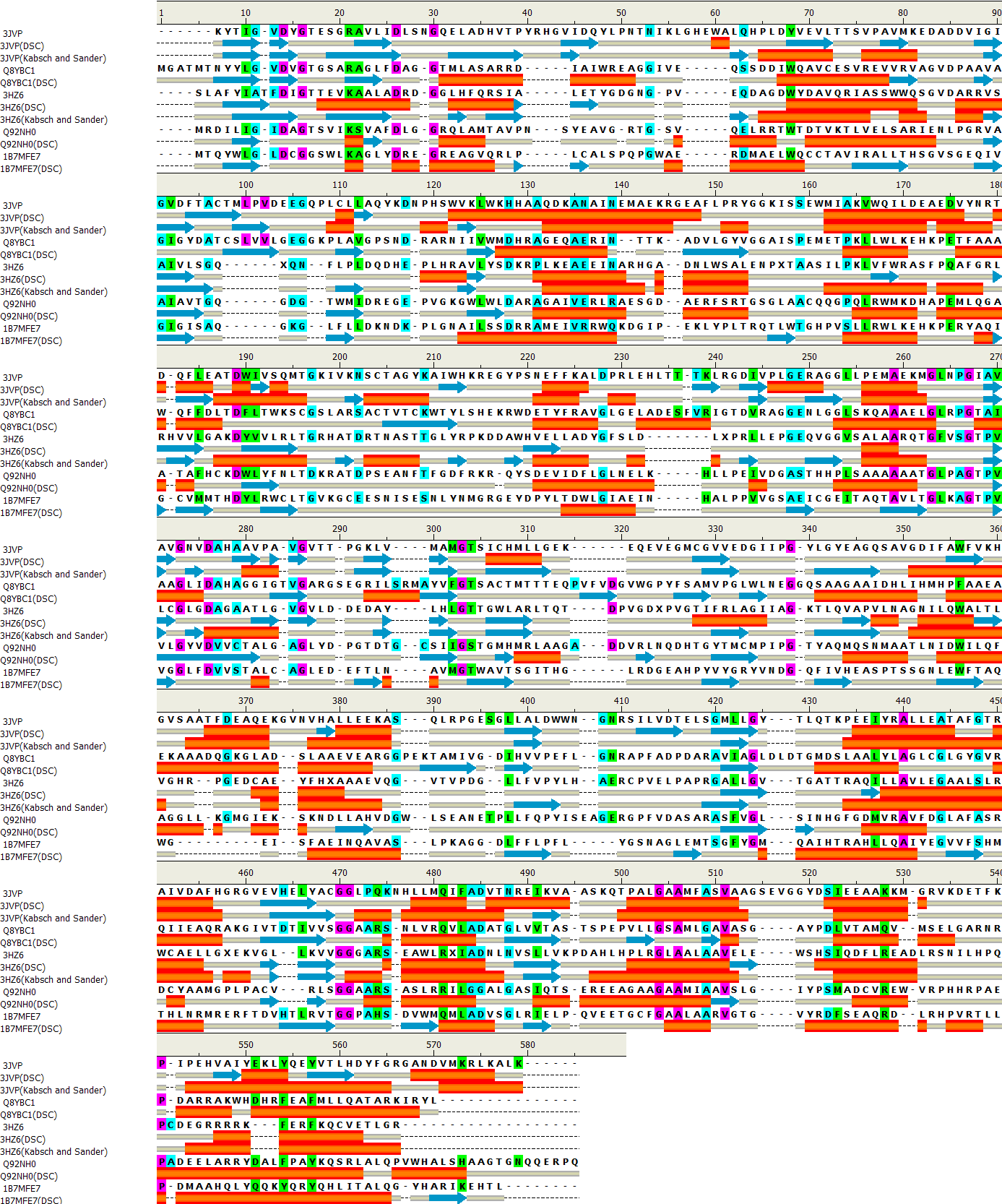
**

3

2

7

5

1

**Figure SEQ4B.** A sequence alignment of the proteins making up the **Group 4** ‘FGGY’ family, within the Type-B subfamily. The indicated numbering is as per the identified residues as outlined in Sup Inf Table SI 4A. Pink = 100% identical, Green = >75% identical, turquoise = >50% identical. Secondary structure elements; Orange/Pink tube = Helix, Blue Arrow = Sheet, Grey = Coil. 3JVP, L-ribulokinase; Q8YBC1, L-ribulokinase; 3HZ6, D-Xylulokinase; Q92NH0, Erythretol kinase; 1B7MFE7, L-Xylulokinase.


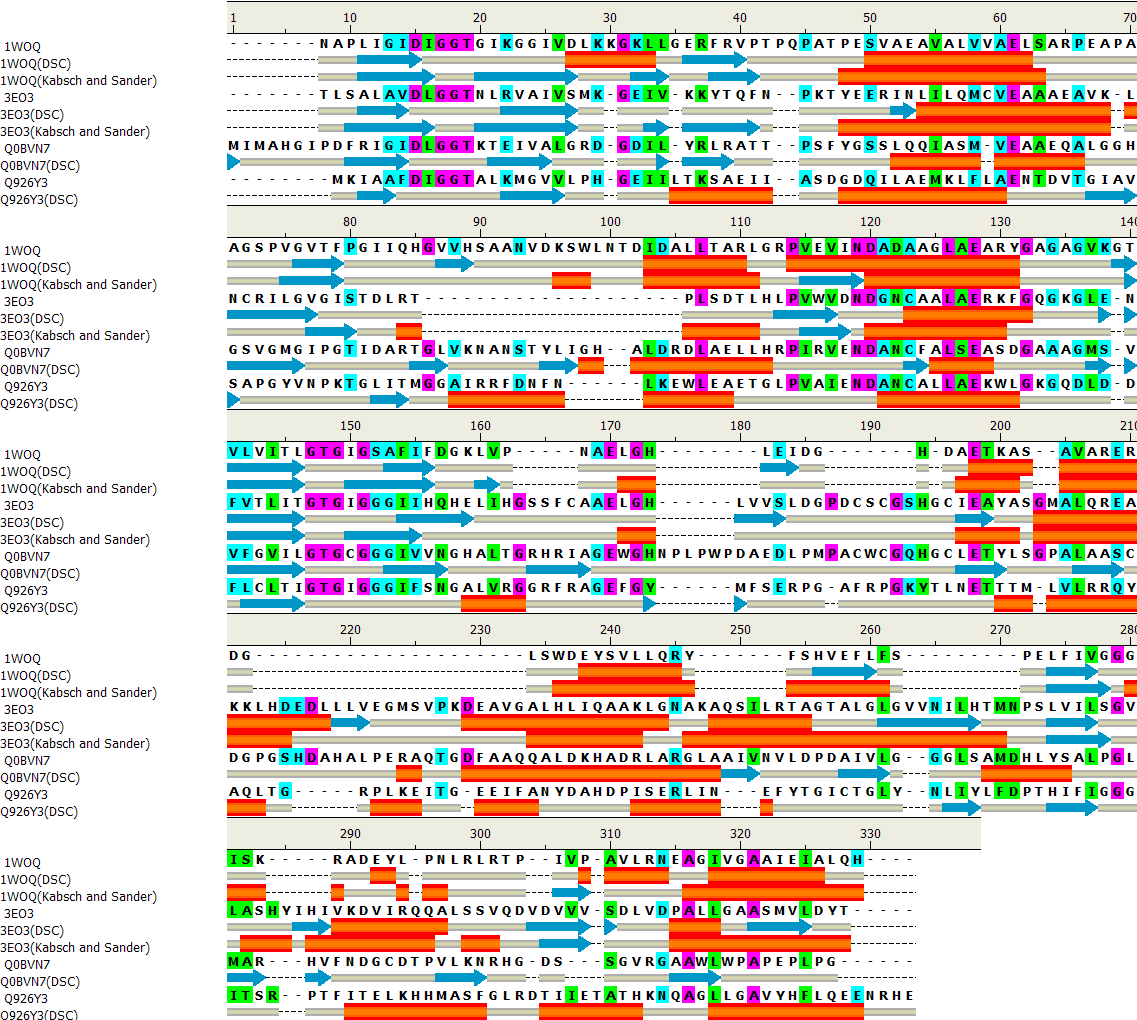


**Figure SEQ4C.** Conserved functional residues within the **Group 4** ROK family of kinases. The indicated numbering is as per the identified residues as outlined in Sup Inf Table SI 4A. Pink = 100% identical, Green = >75% identical, turquoise = >50% identical. Secondary structure elements; Orange/Pink tube = Helix, Blue Arrow = Sheet, Grey = Coil. 1WOQ, Polyphosphate-glucose phosphotransferase; 3EO3, Acetyl-mannoseamine kinase; Q0BVN7, Mannokinase; Q926Y3, β-Glucoside kinase.


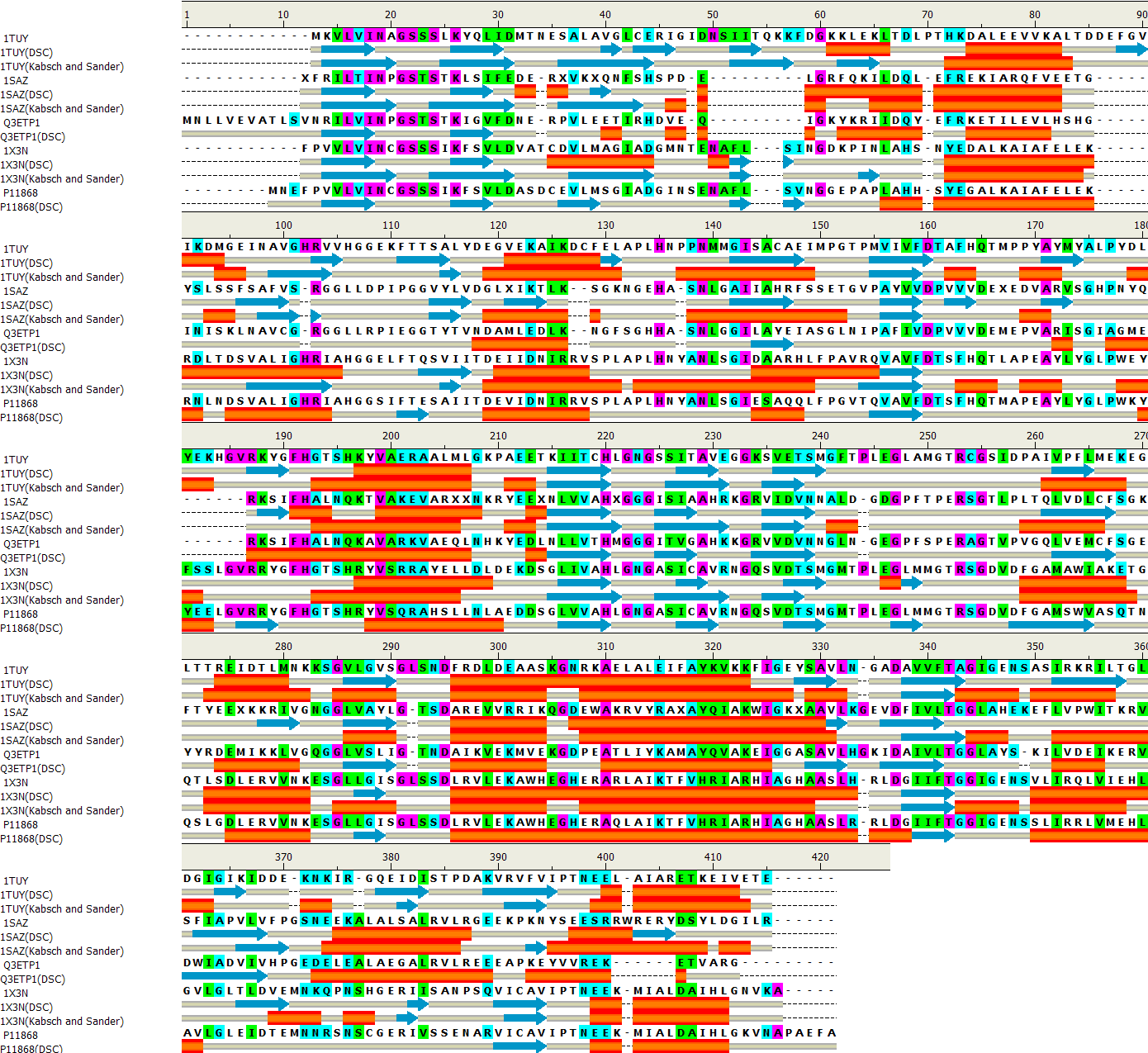


7βb

7α

7γ

7δ

**Figure SEQ4D.** Conserved functional residues within the **Group 4** acetokinase family of kinases. The indicated numbering is as per the identified residues as outlined in Sup Inf Table SI 4A. Pink = 100% identical, Green = >75% identical, turquoise = >50% identical. Secondary structure elements; Orange/Pink tube = Helix, Blue Arrow = Sheet, Grey = Coil. 1TUY, Acetate kinase; 1SAZ, Butyrate kinase; Q3ETP1, Branched-chain-fatty-acid kinase; 1X3N/P11868, Propionate kinase.

7α


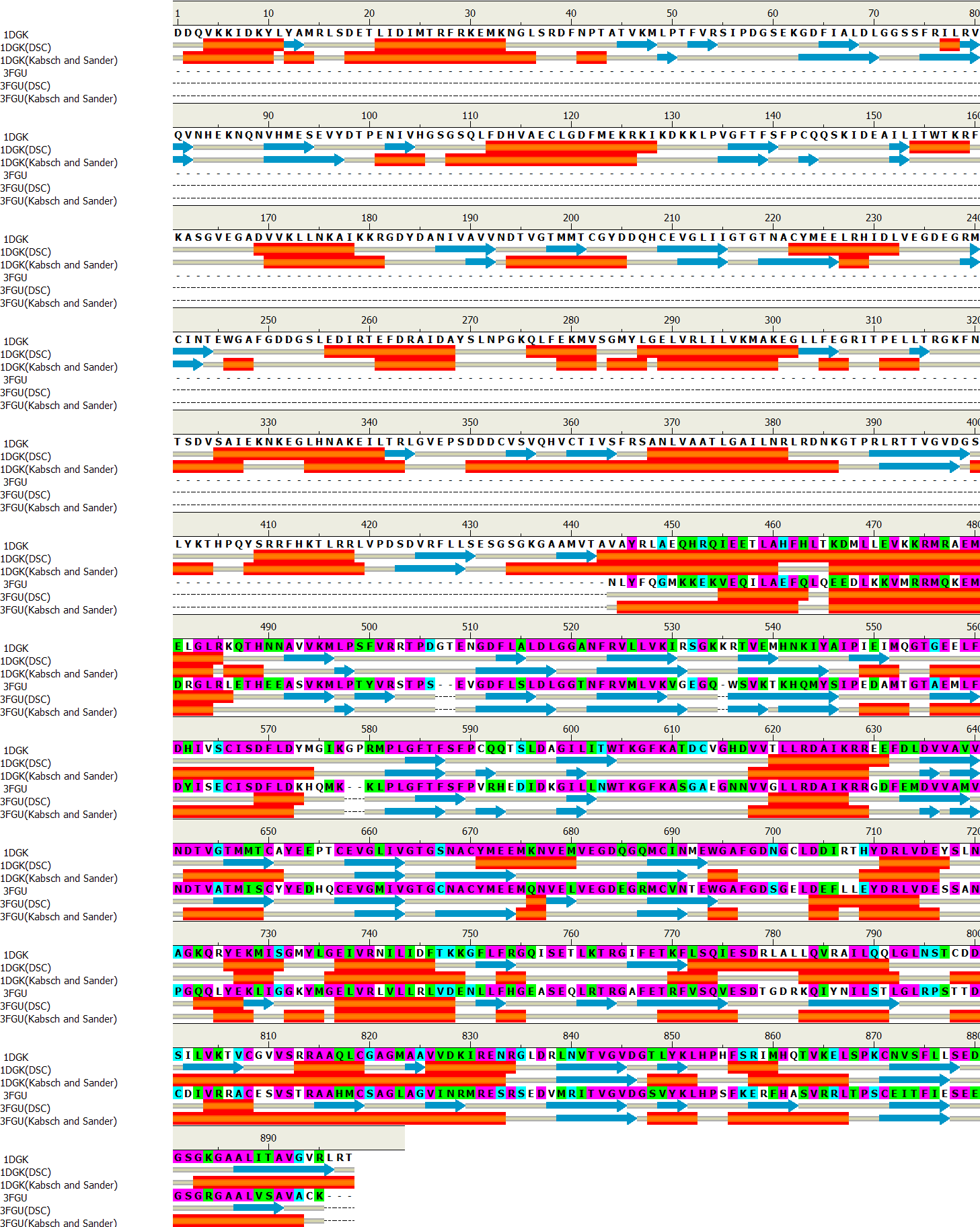


3

2

1

5

**Figure SEQ4E.** Conserved functional residues within the **Group 4** hexokinase family. The indicated numbering is as per the identified residues as outlined in Sup Inf Table SI 4A. Pink = 100% identical, Green = >75% identical, turquoise = >50% identical. Secondary structure elements; Orange/Pink tube = Helix, Blue Arrow = Sheet, Grey = Coil. 1DGK, Hexokinase; 3FGU, Glucokinase.

**Table AF 5A.** Kinases representing **Group 6** and the identified conserved amino acid residues associated with the catalysis of phosphoryl transfer and the measured inter-atomic distances are shown. Also indicated are the residues associated with the rotation of the adenyl moiety from the *syn-* to the *anti-* conformation. The PDB or uniprot accession numbers are indicated. Conserved residues replaced by conserved functionality are indicated by 3-letter code.

| **Kinase** | **Nucleo-tide Config.^1^** | **Asn-δO1 to Aden-NH_2_^2^** | **Lys to Aden C6-NH_2_^3^** | **Lys to Asn-NH_2_^3^** | **Ser to C8-H^4^** | **Ser to α-PO_4_^4^** | **Lys to C8-H^5^** | **Lys to α-PO_4_^5^** | **C8-H to α-PO_4_^6^** | **Lys/Ser to α-PO_4_^7^** | **Lys to β-PO_4_^7^** | **Glu-γC-C=O substrate^8^** |
| --- | --- | --- | --- | --- | --- | --- | --- | --- | --- | --- | --- | --- |
| **GHMP kinase** | | | | | | | | | | | | |
| Galactokinase 1S4E ([*Pyrococcus furiosus*)](http://www.rcsb.org/pdb/search/smartSubquery.do?smartSearchSubtype=TreeEntityQuery&t=1&n=2261) | *Syn-* | His51 3.412 | Tyr35 2.735 | Tyr35 4.151 | Ser106 7.155 | Ser106 5.044 |  |  |  |  |  |  |
|  | ***Anti-*** | His51 4.354 | Tyr35 4.171 | Tyr35 7.040 | Ser106 2.860 | Ser106 4.069 |  |  |  | 9 | 9 |  |
| Mevalonate kinase 2V8P ([*Aquifex aeolicus*)](http://www.rcsb.org/pdb/search/smartSubquery.do?smartSearchSubtype=TreeEntityQuery&t=1&n=63363) | ***Syn-*** | Asn99  2.180 | Lys 85 2.934 | Lys85 3.465 | Ser97 8.582 | Ser97 3.574 | - | - | - | - | - |  |
|  | *Anti-* | Asn99  4.782 | Lys 85 6.068 | Lys 85 5.056 | Ser97 4.096 | Ser97 2.990 | Lys85 3.057 | Lys85 3.326 | 3.030D | Ser97 2.990 | Ser97 5.286 | Asp130 |
| Homoserine kinase 1FWK ([*Methanocaldococcus jannaschii*)](http://www.rcsb.org/pdb/search/smartSubquery.do?smartSearchSubtype=TreeEntityQuery&t=1&n=2190) | ***Syn-*** | Asn62  1.971 | Lys 87 3.046 | Lys87 5.715 | Ser98 6.375 | Ser98 4.511 | - | - | - |  |  |  |
|  | *Anti-* | Asn62  3.549 | Lys 87 3.325 | Lys 87 7.370 | Ser98 2.791 | Ser98 2.313 | Lys87 4.797 | Lys87 6.012 | 4.781^9^ | Ser98 2.386 | Ser98 3.153 | Asp140 |
| L-arabinokinase O23461 (*Arabidopsis thaliana*) |  | NS^7^ |  |  |  |  |  |  |  |  |  |  |
| Fucokinase Q8N0W3 (*Homo sapiens*) |  | NS^7^ |  |  |  |  |  |  |  |  |  |  |
| Shikimate kinase^11^ |  | NS^7^ |  |  |  |  |  |  |  |  |  |  |
| ISPE^12^ 2V2Z ([*Aquifex aeolicus*)](http://www.rcsb.org/pdb/search/smartSubquery.do?smartSearchSubtype=TreeEntityQuery&t=1&n=63363) | ***Syn-*** | Asn99 1.919 | Lys 85 3.324 | Lys85 3.710 | Ser97 9.325 | Ser97 3.658 | - | - | - |  |  |  |
|  | *Anti-* | Asn99 4.344 | Lys 85 6.208 | Lys85 4.377 | Ser97 6.054 | Ser97 4.431 | Lys85 4.381 | Lys85 4.278 | 4.672 | Ser97 | Ser97 |  |
| Phosphomevalonate kinase 1GON ([*Streptomyces* sp.](http://www.rcsb.org/pdb/search/smartSubquery.do?smartSearchSubtype=TreeEntityQuery&t=1&n=1931)) | *Syn-* | Met35 3.066 | Lys 94 2.545 | Lys94 4.053 | - | Ser106 5.698 | - | - | - |  |  |  |
|  | ***Anti-*** | Met35 4.107 | Lys 94 6.098 | Lys94 9.114 | Ser106 1.750 | Ser106 1.675 | Lys94 7.220 | Lys94 7.495 | 2.716 | Lys101 4.285 | Lys101 2.810 | Asp150 |
|  |  |  |  |  |  |  |  |  |  |  |  |  |
| **Mean** |  | **4.2272** | **5.174** | **6.591** | **3.510** | **3.096** | **4.864** | **5.278** | **3.800** | **3.220** | **3.750** |  |
| **Standard Deviation** |  | **0.451** | **1.337** | **1.899** | **1.647** | **1.159** | **1.737** | **1.850** | **1.079** | **0.970** | **1.341** |  |
| **% Standard Deviation** |  | **10.658** | **25.835** | **28.817** | **46.925** | **37.438** | **35.718** | **35.047** | **28.388** | **30.128** | **35.777** |  |
|  |  |  |  |  |  |  |  |  |  |  |  |  |

1. Bold type indicates configuration of adenyl moiety relative to ribose sugar in the coordinated structure in the PDB.
2. Coordination of the carbonyl the γ-carbonyl of Asn to the hydrogen of the C6-NH_2_ of the nucleotide with the Asn carbonyl acting as a general base catalyst.
3. Coordination of the Lys ε-NH_3_ to the γ-carbonyl of Asn and the hydrogen of the C6-NH_2_ of the nucleotide.
4. α- and β-PO_4_ coordinated Ser/Thr.
5. Lysine coordination to C8-H and α-PO_4_.
6. Direct transfer from C8-H to β-PO_4_.
7. α- and β-PO_4_ proton transfer.
8. Asp responsible for substrate deprotonation.
9. C8-H to β-PO_4_ transfer therefore no residue required.
10. No structure in PDB.
11. archaeal shikimate kinase.
12. 4-(cytidine 5’-diphospho)-2-C-methyl-D-erythritol kinase.

**Figure MECH8.** The postulated phosphoryl transfer mechanism found in the Group 6 kinases differs from all the other groups in that the adenyl group is in a *syn* conformation relative to the ribose, allowing for the coordination of the C8-H to the α-PO_4_. The reaction is initiated by the coordination of the conserved Asn γC-carbonyl group to the ATP C6-NH_2_. The coordination of a conserved lysine with the concomitant delocalization of the electrons of the adenyl group results in the re-hybridization of C8 from sp^2^ to sp^3^, that, along with the change of the conformation of the adenyl group from the *syn* to the *anti*-conformation there allows for the protonation of C8 by the lysine residue forming the carbene with the concomitant protonation of the α-PO_4_ from C8. The proton translocation from the α-PO_4_ creating the pentavalent intermediate then occurs. The proton required for the creation of the pentatvalent intermediate originates from the substrate via the Glu/Asp deprotonation of the substrate.

**Table AF 5B.** Group 6 kinases

|  | **SUPERFAMILY^1^** | **FAMILY / DOMAIN^2^** |
| --- | --- | --- |
| **Group 6 Kinases** | | |
| 1. Mevalonate kinase (pdb2V8P) | Ribosomal protein S5 s-like (CL0329) | GHMP^4^ kinase N terminal domain (PF00288)  GHMP kinase C terminal domain (PF08544) |
| 1. Homoserine kinase (pdb1FWK) | Ribosomal protein S5 s-like (CL0329) | GHMP kinase N terminal domain (PF00288)  GHMP kinase C terminal domain (PF08544) |
| 1. L-arabinokinase (No sequence) | Ribosomal protein S5 s-like (CL0329) | GHMP kinase N terminal domain (PF00288)  GHMP kinase C terminal domain (PF08544) |
| 1. Fucokinase (uniprotQ8N0W3) | Ribosomal protein S5 s-like (CL0329) | GHMP kinase N terminal domain (PF00288)  GHMP kinase C terminal domain (PF08544) |
| 1. Shikimate kinase (No sequence) | Ribosomal protein S5 s-like (CL0329) | GHMP kinase N terminal domain (PF00288)  GHMP kinase C terminal domain (PF08544) |

^1^ pfam clan classification in brackets

^2^ pfam family/domain classification in brackets

^3^ No Detectable similarity to conventional kinases

^4^ GHMP family includes galactokinases, homoserine kinases and mevalonate kinases

Where there are 2 or domains recognised, these are denoted by ‘a’, ‘b’, etc. One domain has been selected to position the protein within the table.


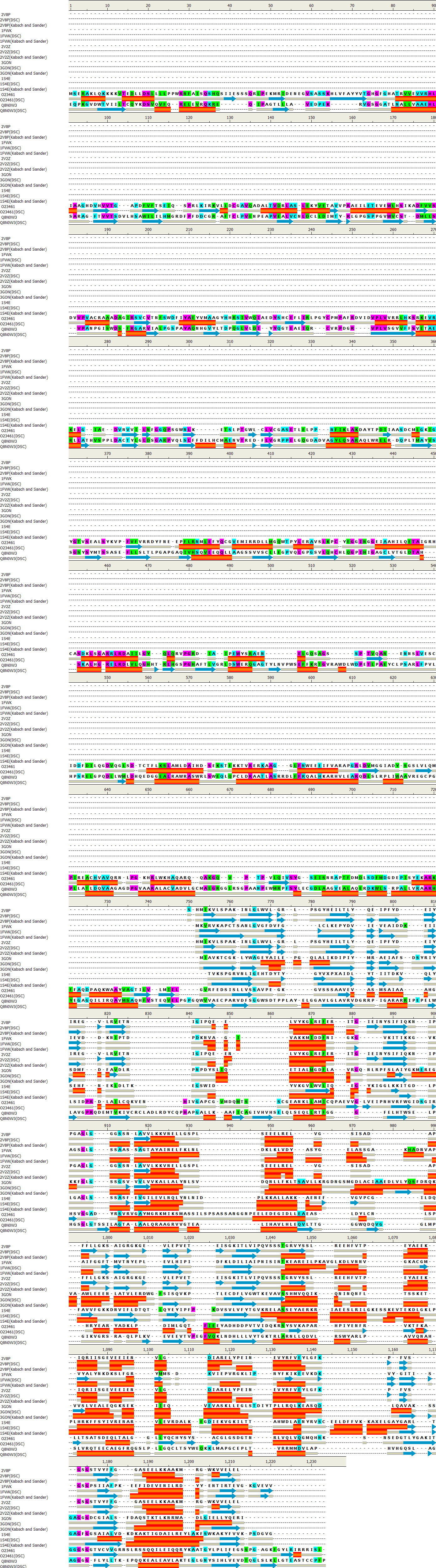


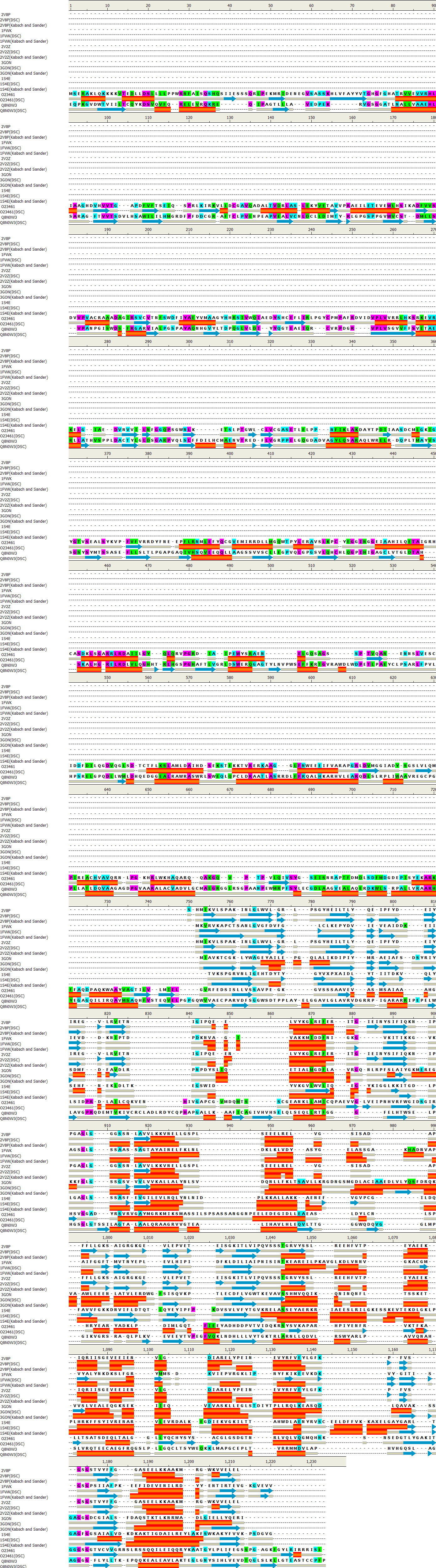


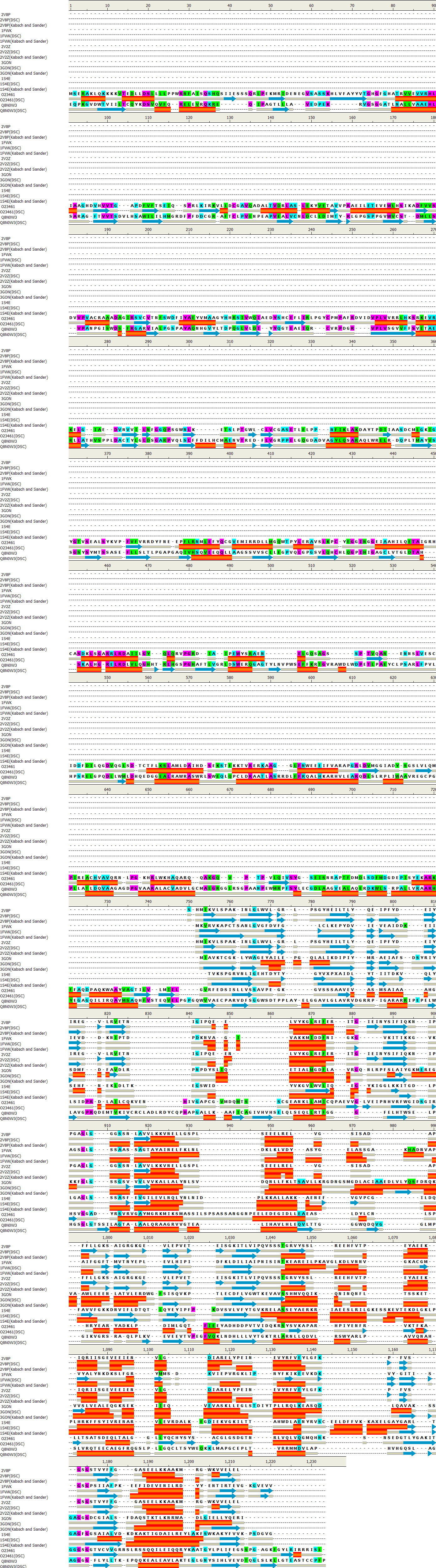


**2**

**3**


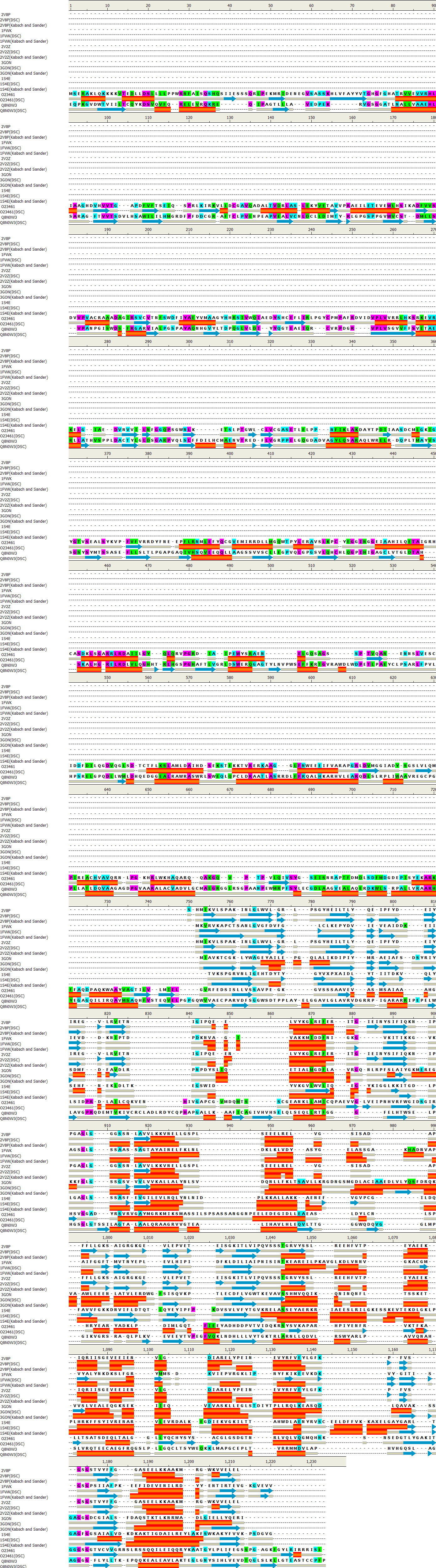


**3**

**2**

**2**

**4**

**2**

**5**


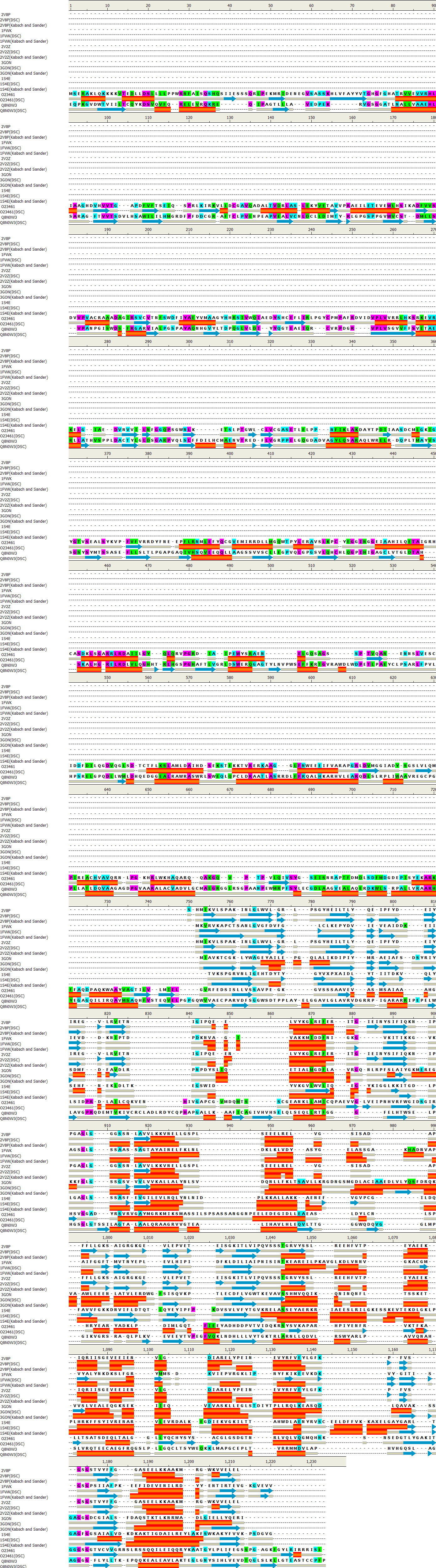


**Figure SEQ9**. Conserved functional residues within the **Group 6** kinases. The indicated numbering is as per the identified residues as outlined in Sup Inf Table SI 4A. Pink = 100% identical, Green = >75% identical, turquoise = >50% identical. Secondary structure elements; Orange/Pink tube = Helix, Blue Arrow = Sheet, Grey = Coil. 1SAE, Galactokinase; 2V8P, Mevalonate kinase; 1FWK, Homoserine kinase; 3GON, Phosphomevalonate kinase; O23461, L-arabinokinase; Q8N0W3, Fucokinase.

**Table AF 6A**. Kinases representing **Group 7** and the identified conserved amino acid residues associated with the catalysis of phosphoryl transfer and the measured inter-atomic distances are shown. The PDB or uniprot accession numbers are indicated. Conserved residues replaced by conserved functionality are indicated by 3-letter code.

| **Kinase** | **Asn- αC-C=O to Aden-NH_2_^1^** | **C8-H to α-PO_4_^2^** | **α-PO_4_ to β-PO_4_^3^** | **His^4^** | **Asp^5^** | **Asp^6^** | **Asp^7^** | **Loop** |
| --- | --- | --- | --- | --- | --- | --- | --- | --- |
| **Group 7:AIR synthetase-like sequences** | | | | | | | |  |
| Thiamine phosphate kinase 3C9S (*Aquiflex aeolicus*) | AsnB119  2.549 | 3.774 | 3.092 | HisA219 4.132 | Asp210 3.822 | Asp43 3.490 | Substrate 3.308 | ValB115-SerB121 Gly118 2.299 |
| Selenide, water dikinase 3FD6 (*Homo sapiens*) | MetA124  2.730 | 3.413 | 1.700 | LysB32 1.700 | AspB69 5.519 | AspB110 5.443 | AspB265 3.104 | SerA158-ValA165 GlyA162 2.244 |
| Selenide, water dikinase 2YYE (*Aquiflex aeolicus*) | HisB132 3.196 | 3.404 | 2.622 | HisA132 2.413 | AspA60 2.718 | AspA83 2.835 | AspA219 2.943 | LeuB128-ThrB133 GlyB131 2.408 |
| NAD^+^ kinase 1Z0S **FROM GROUP 2 (**[*Archaeoglobus fulgidus*)](http://www.rcsb.org/pdb/search/smartSubquery.do?smartSearchSubtype=TreeEntityQuery&t=1&n=2234) | Ala180 2.746 | Met127 2.973 | Met127 3.487 | β-PO_4_ 4.084 |  |  |  | Direct transfer to β-PO_4_ |
|  |  |  |  |  |  |  |  |  |
| **Mean** | **2.825** | **3.883** | **2.471** | **2.748** | **4.020** | **3.923** | **3.118** |  |
| **Standard Deviation** | **0.334** | **0.822** | **0.708** | **1.250** | **1.411** | **1.357** | **0.183** |  |
| **% Standard Deviation** | **11.816** | **21.166** | **28.654** | **45.489** | **35.101** | **34.588** | **5.866** |  |

1. Coordination of the carbonyl of the backbone carbon of Asn to the hydrogen of the C6-NH_2_ of the nucleotide with the Asn carbonyl acting as a general base catalyst.
2. Nucleotide adenyl C8-H coordination to α-PO_4_.
3. Interatomic distance between α-PO_4_ and β-PO_4_ allowing for direct proton transfer.

4.-7. on substrate binding, the deprotonation of the substrate by a Asp residue leads to the inter-aspartate transfer of the proton to a coordinated His/Lys which acts to transfer the H^+^ to the α-PO_4_, the primary step in the creation of the pentavalent intermediate.

**Figure MECH10.** Phosphoryl transfer mechanism found in the Group 7 kinases differs from the other kinase groups in that the active site is dependent on the formation of the dimer between two subunits. This allows the reaction to be initiated via the coordination of a carbonyl arising from the protein backbone to the ATP C6-NH_2_ on the one subunit (B) as does a conserved backbone carbonyl which acts to stabilize the carbene formed at C8, and the remainder of the coordinating residues arising from the second subunit (A). The reaction mediated by thiamine monophosphate kinase is initiated by the binding of the thiamine monophosphate. Once protonation has occurred from C8-H to the α-PO_4_, the substrate is protonated via a cascade involving a conserved His residue and a series of Asp residues. The protonation of the substrate allows the formation of the pentavalent intermediate, the migration of the Mg^2+^ from being the β-PO_4_/γ-PO_4_ coordinated to being α-PO_4_/β-PO_4_ coordinated, and the concomitant re-protonation of C8.

**Table AF 7A**. Kinases representing **Group 8** and the identified conserved amino acid residues associated with the catalysis of phosphoryl transfer and the measured inter-atomic distances are shown. The PDB or uniprot accession numbers are indicated. Conserved residues replaced by conserved functionality are indicated by 3-letter code.

| **Kinase** | **AA-αC-C=O to Aden-NH_2_^1^** | **Asp to C8-H ^1^** | **Asp/Arg^2^** | **Lys to C8-H^3^** | **Arg/Tyr to β**-PO_4_**^4^** | **Glu^4^** |
| --- | --- | --- | --- | --- | --- | --- |
| **Group 8:Riboflavin kinase** | | | | | | |
| Riboflavin kinase^§#^ 1Q9S (*Homo sapiens*) | His91 2.547 | Asp96 2.955 | Asp96/Arg21 5.201 | His88 6.155 | Arg21  3.642 | Glu86 |
| Riboflavin kinase^§#^ 1N06 ([*Schizosaccharomyces pombe*)](http://www.rcsb.org/pdb/search/smartSubquery.do?smartSearchSubtype=TreeEntityQuery&t=1&n=4896) | Leu99 1.723 | Asp106 2.867 | Asp106/Tyr108 4.642 | Tyr108 3.799 | Tyr108  1.990 | Glu96 |
| Riboflavin kinase^§#^ 1N08 ([*Schizosaccharomyces pombe*)](http://www.rcsb.org/pdb/search/smartSubquery.do?smartSearchSubtype=TreeEntityQuery&t=1&n=4896) | Leu99 1.747 | Asp106 3.187 | Asp106/Tyr108 4.491 | Tyr108 3.524 | Tyr108  3.732 | Glu96 |
| Riboflavin kinase^§#^ 1NB0 (*Homo sapiens*) | His91 3.163 | Asp96 3.404 | Asp96/Arg21 4.123 | Lys28 2.716 | Arg21  4.324 | Glu86 |
|  |  |  |  |  |  |  |
| **Mean** | **2.211** | **3.153** | **6.250** | **3.346** | **4.3957** |  |
| **Standard Deviation** | **0.825** | **0.270** | **1.870** | **0.562** | **1.276** |  |
| **% Standard Deviation** | **37.293** | **8.569** | **29.918** | **16.823** | **29.028** |  |

^§^ inter-subunit in interaction.

1. Asp carboxyl group coordination to C8-H.
2. Asp to Arg coordination.
3. Arg to β-PO_4_ coordination.
4. Lys to C8-H
5. deprotonation of the substrate by Glu.

# rotation of histidine N-Cα-Cβ-Cγ torsion angle for optimum interatomic distance.

**Figure AF 11.** The postulated phosphoryl transfer mechanism found in the Group 8 kinases is similar to all other groups utilizing the “push“mechanism (flavokinase family). The mechanism occurs via coordination of the adenyl C6-NH_2_ and protonation of C8 via a coordinated Lys changing C8 from sp2 to sp3 hybridization, and alters the protonation of C8-H. The C8-H becomes more acidic, allowing for the protonation of the β-PO_4_, via a conserved Asp to Arg proton transfer. The H^+^ transfer is directly to the β-PO_4_, facilitating the formation of the pentavalent intermediate between the γ-PO_4_ and the substrate nucleophile. There is a simultaneous Glu-mediated deprotonation of the substrate-OH that allows for the nucleophilic attack by the substrate, creating the pentavalent intermediate and allowing phosphoryl transfer. The protonated Glu then transfers the proton to the γ-PO_4_ changing the coordination of the Mg^2+^ from being β-PO_4_ to γ-PO_4_ coordinated to being α-PO_4_ to β-PO_4_ coordinated. The H^+^ originally arising from the C8 is then transferred back to C8, allowing the electron density of the adenyl moiety to return to the “ground-state” distribution.


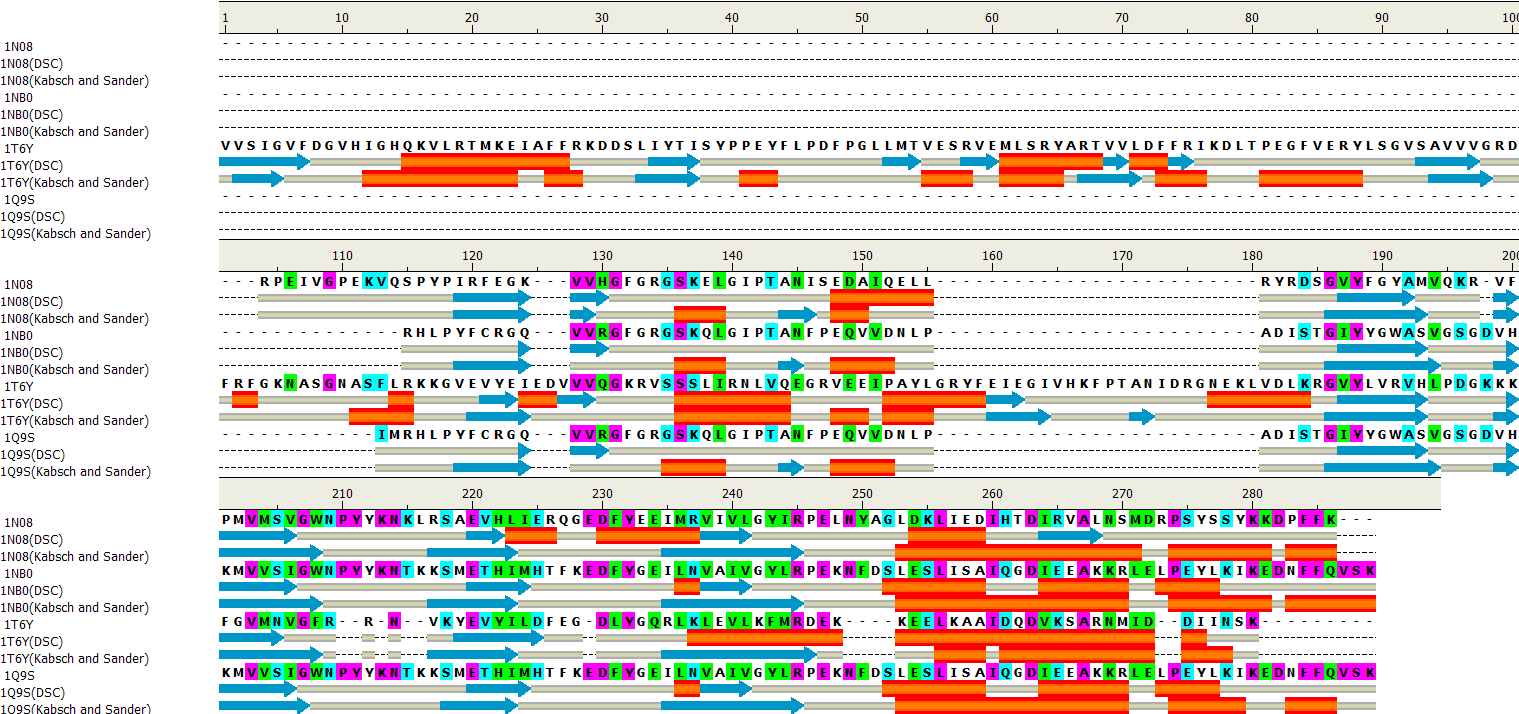


**3**

**3**

**2**

**1**

**2**

**Figure SEQ12**. Conserved functional residues within the **Group 8** kinases. The indicated numbering is as per the identified residues as outlined in Sup Inf Table SI 7A. Pink = 100% identical, Green = >75% identical, turquoise = >50% identical. Secondary structure elements; Orange/Pink tube = Helix, Blue Arrow = Sheet, Grey = Coil. 1N08; [*Schizosaccharomyces pombe*,](http://www.rcsb.org/pdb/search/smartSubquery.do?smartSearchSubtype=TreeEntityQuery&t=1&n=4896)  1NB0; *Homo sapiens*, 1Q9S; *Homo sapiens*.

**Table AF 8A**. Kinases representing **Group 9** **– 10** and the identified conserved amino acid residues associated with the catalysis of phosphoryl transfer and the measured inter-atomic distances are shown. The PDB or uniprot accession numbers are indicated. Conserved residues replaced by conserved functionality are indicated by 3-letter code.

| **Kinase** | **EC** | **Met^1^** | **Thr^2^** | **His^3^** | **Thr^4^** |  |  |  |  |  |  |  |  |
| --- | --- | --- | --- | --- | --- | --- | --- | --- | --- | --- | --- | --- | --- |
| **GROUP 9: Dihydroxyacetone kinase** | | | | | |  |  |  |  |  |  |  |  |
| Dihydroxyacetone kinase ^§#^ 2BTD **(**[*Escherichia coli*)](http://www.rcsb.org/pdb/search/smartSubquery.do?smartSearchSubtype=TreeEntityQuery&t=1&n=469008) | 2.7.1.29 | 477 | 476 | **Direct** | 480 |  |  |  |  |  |  |  |  |
| **GROUP 10: Glycerate kinase** | | | | | |  |  |  |  |  |  |  |  |
| Glycerate kinase ^4^ ([*Neisseria meningitidis*)](http://www.rcsb.org/pdb/search/smartSubquery.do?smartSearchSubtype=TreeEntityQuery&t=1&n=65699) | 2.7.1.31 | No ADP |  |  |  |  |  |  |  |  |  |  |  |
| **Kinase** | **AA-αC-C=O to Aden-NH_2_** | **N7 to Arg^1^** | **C8-H to Arg^1^** | **Arg^1^ Nε to α-PO_4_** | **Arg^1^ Nε to α-PO_4_** | **C8-H to α-PO_4_** | **Arg^2^-NH_3_ to α -PO_4_** | **Arg^2^-NH_3_ to β-PO_4_** | **1^st^ His^3^ to γ-PO_4_** | **Glu^4^ to His** | **2^nd^ His to γ-PO_4_^5^** | **Glu to His^6^** | **Asn-γC-C=O to C8-H^7^** |
| **GROUP 11: Polyphosphate kinase (legend as per Group 3 kinases)** | | | | | |  |  |  |  |  |  |  |  |
| Polyphosphate kinase 1XDP  From Group 9 ([*Escherichia coli*)](http://www.rcsb.org/pdb/search/smartSubquery.do?smartSearchSubtype=TreeEntityQuery&t=1&n=562) | Asp587 3.287 | Arg564 3.213 | Arg564 2.614 | Arg564 3.003 | Arg564 3.003 | 7.406 | Arg405 2.994 | Arg4053.353 | His435 2.901 | Glu623 1.944 | His592 2.901 | Asp470 1.667 | Asn45 2.906 |
| Polyphosphate kinase 2O8R  ([*Porphyromonas gingivalis*)](http://www.rcsb.org/pdb/search/smartSubquery.do?smartSearchSubtype=TreeEntityQuery&t=1&n=837) |  | Arg568 | Arg568 | Arg568 | Arg568 |  | Arg407 | Arg407 | His 437 | Glu626 | His595 | Asp474 | Asn46 |
| **GROUP 12: Integral membrane kinases** | | | | | |  |  |  |  |  |  |  |  |
| No structures. |  |  |  |  |  |  |  |  |  |  |  |  |  |

^§^ two orientations of the ANP in the same molecule.

1. C8-H coordinated.
2. N7 coordinated.
3. C8-H to β-PO_4_ coordinated.
4. No ATP in structure.

# rotation of adenine around the adenine/ribose bond.

**Figure AF 13.** The postulated phosphoryl transfer mechanism found in the Group 11 kinases which utilizes the “push“mechanism but also undergoes an autophosphorylation of a histidine residue (polyphosphate family). The mechanism occurs via coordination of the adenyl C6-NH_2_ from the protein backbone to the ATP C6-NH_2_ and an Asn carbonyl which acts to stabilize the carbene formed at C8. The C8-H becomes more acidic, allowing for the protonation of the α-PO_4_, via a conserved Arg proton transfer. A second conserved Arg transfers the proton from the α-PO_4_ to the γ-PO_4_ facilitating the formation of the pentavalent intermediate and to allow for phosphoryl transfer. There is a simultaneous deprotonation of a conserved His allowing for the nucleophilic attack of the His for the γ-PO_4_. There is also a putative deprotonation of the substrate phosphate by a second His allowing for the concomitant formation of the phosphate dimer (polymer). The H^+^ originally arising from the C8 is then transferred back to C8, allowing the electron density of the adenyl moiety to return to the “ground-state” distribution.


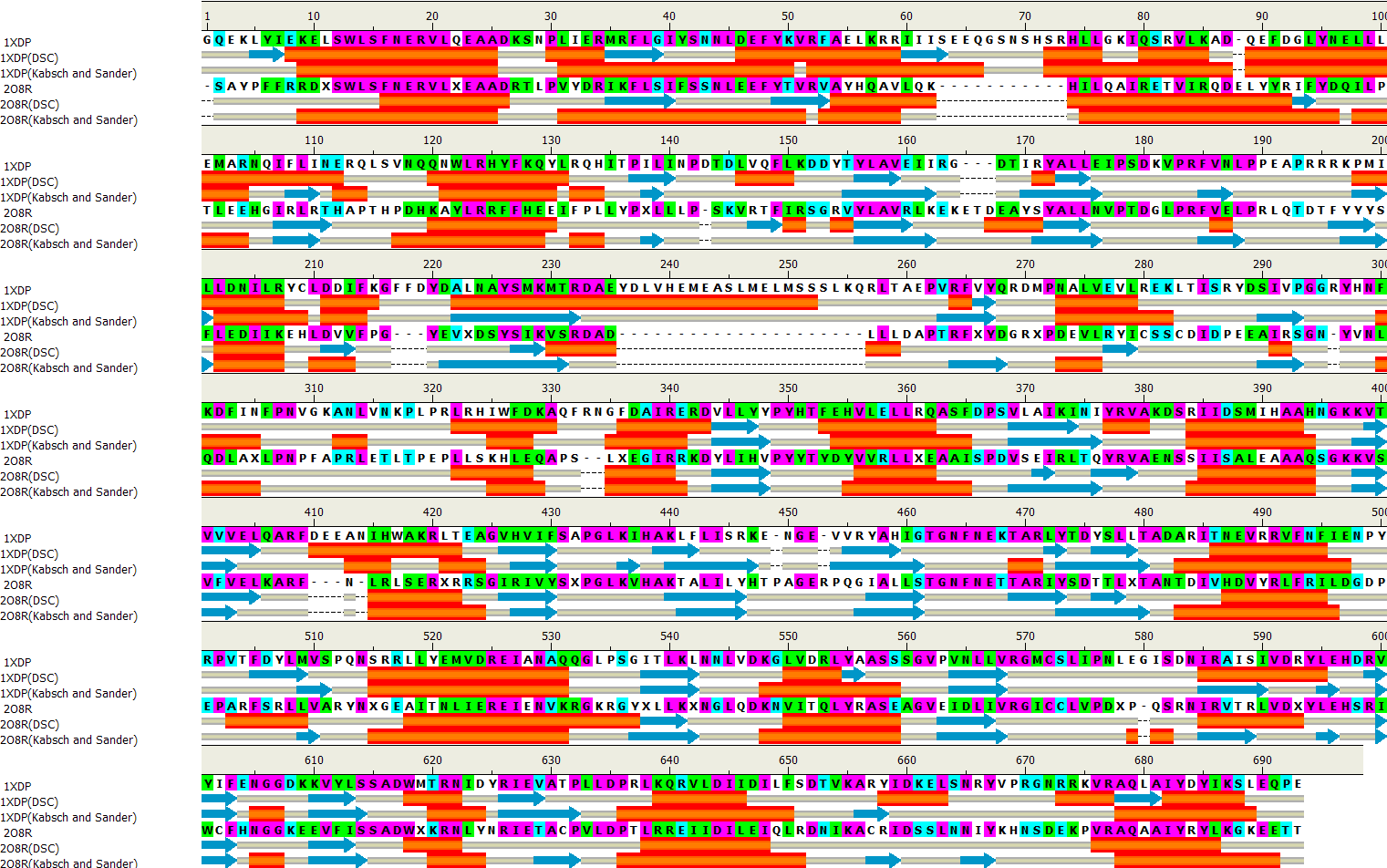


**1**

**3**

**2**

**4**

**5**

**6**

**7**

**Figure SEQ14**. Conserved functional residues within the **Group 11** kinase family. The indicated numbering is as per the identified residues as outlined in Sup Inf Table SI 8A. Pink = 100% identical, Green = >75% identical, turquoise = >50% identical. Secondary structure elements; Orange/Pink tube = Helix, Blue Arrow = Sheet, Grey = Coil. 1XDP;  [*Escherichia coli*,](http://www.rcsb.org/pdb/search/smartSubquery.do?smartSearchSubtype=TreeEntityQuery&t=1&n=4896)  2O8R; *Porphyromonas gingivalis*.

**References (**Additional file 1**)**

References outlined below refer to the crystal structures used for the analysis of all active sites in this investigation. Included are the RCSB protein databank accession codes for each protein used.

# Hong, B. S., Allali_Hassani, A., Templ, W., Finerty Jr., P. J., Mackenzie, F., Domiv, S., Vedadi, M. and Park, H. W. (2010) Crystal structures of human choline kinase isoforms in complex with hemicholinium-3: single amino acid near the active site influences inhibitor sensitivity. [***J. Biol. Chem.***](javascript:AL_get(this, 'jour', 'J Biol Chem.');) *285*, 16330-16340. (3G15.pdb)

1. Debreczeni, J.E., Bullock, A., Knapp, S., Von Delft, F., Sundstrom, M., Arrowsmith, C., Weigelt, J. and Edwards, A. (2010) PIM kinase. Structural Genomics Consortium, Oxford. Journal: To be Published. (2BZK.pdb)
2. Lowe, E.D., Noble, M.E., Skamnaki, V.T., Oikonomakos, N.G., Owen, D.J. and Johnson, L.N. (1997) The crystal structure of a phosphorylase kinase peptide substrate complex: kinase substrate recognition*. EMBO J.* *16*, 6646-6658. (2PHK.pdb)
3. Thorsell, A.G., Uppenberg, J., Hogbom, M., Ogg, D., Arrowsmith, C., Berglund, H., Collins, R., Edwards, A., Ehn, M., Flodin, S., Flores, A., Graslund, S., Hammarstrom, M., Kotenyova, T., Nilsson-Ehle, P., Nordlund, P., Nyman, T., Sagemark, J., Stenmark, P., Sundstrom, M., Van Den Berg, S., Weigelt, J., Holmberg-Schiavone, L., Persson, C.,   Hallberg, B.M. (2010) Structure of Human Phosphatidylinositol-4-phosphate 5-kinase, type II, gamma. Journal**:** To be Published. (2GK9.pdb)
4. Fong, D.H. and Berghuis, A.M. (2002) Substrate promiscuity of an aminoglycoside antibiotic resistance enzyme via target mimicry. *EMBO J. 21,* 2323-2331. (1L8T.pdb)
5. Ku, S.Y., Smith, G.D. and Howell, P.L. (2007) ADP-2Ho as a phasing tool for nucleotide-containing proteins. *Acta Crystallogr.,Sect.D. 63,* 493-499. (2OLC.pdb)
6. Lougheed, J.C., Chen, R.H. Mak, P. and Stout, T.J. (2004) Crystal structures of the phosphorylated and unphosphorylated kinase domains of the Cdc42-associated tyrosine kinase ACK1. *J. Biol. Chem. 279***,** 44039-44045. (1U54.pdb)
7. Qiu, W., Hutchinson, A., Wernimont, A., Walker, J.R., Sullivan, H., Lin, Y.-H., Mackenzie, F., Kozieradzki, I., Cossar, D., Schapira, M., Senisterra, G., Vedadi, M., Arrowsmith, C.H., Bountra, C., Weigelt, J., Edwards, A.M., Bochkarev, A., Hui, R. and Amani, M. (2010) Crystal structure of full length CpCDPK3 (cgd5_820) in complex with Ca2+ and AMPPN. Journal: To be Published. (3LIJ.pdb)
8. Singh, P. and Tesmer, J.J.G. (2008) Crystal Structure of G protein coupled receptor kinase 1 bound to ATP and magnesium chloride at 2.7A. Journal: To be Published. (3C4W.pdb)
9. Lodowski, D.T., Barnhill, J.F., Pyskadlo, R.M., Ghirlando, R., Sterne-Marr, R. and Tesmer, J.J.G. (2005) The role of G beta gamma and domain interfaces in the activation of G protein-coupled receptor kinase 2. *Biochemistry 44*, 6958-6970. (1YM7.pdb)
10. Walker, E.H., Pacold, M.E., Perisic, O., Stephens, L., Hawkins, P.T., Whymann, M.P. and Williams, R.L. (2000) Structural determinants of phosphoinositide 3-kinase inhibition by wortmannin, LY294002, quercetin, myricetin, and staurosporine. *Mol.Cell 6,* 909-919. (1E8X.pdb)
11. Liu, L., Song, X., He, D., Komma, C., Kita, A., Virbasius, J.V., Huang, G., Bellamy, H.D., Miki, K., Czech, M.P. and Zhou, G.W. (2006) Crystal structure of the C2 domain of class II phosphatidylinositide 3-kinase C2alpha. *J. Biol. Chem. 281***,** 4254-4260. (2B3R.pdb)
12. Rao, V.D., Misra, S., Boronenkov, I.V., Anderson, R.A. and Hurley, J.H. (1998) Structure of type IIbeta phosphatidylinositol phosphate kinase: a protein kinase fold flattened for interfacial phosphorylation. *Cell 94*, 829-839. (1BO1.pdb)
13. Gonzalez, B., Schell, M.J., Letcher, A.J., Veprintsev, D.B., Irvine, R.F. and Williams, R.L. (2004) Structure of a human inositol 1,4,5-trisphosphate 3-kinase: substrate binding reveals why it is not a phosphoinositide 3-kinase. *Mol.Cell. 15***,** 689-701. (1W2C.pdb)
14. Holmes, W., and Jogl, G. (2006) Crystal structure of inositol phosphate multikinase 2 and implications for substrate specificity. *J. Biol. Chem. 281*, 38109-3811. (2IF8.pdb)
15. Structure of Human Phosphatidylinositol-4-phosphate 5-kinase, type II, gamma
16. Thorsell, A.G., Uppenberg, J., Hogbom, M., Ogg, D., Arrowsmith, C., Berglund, H., Collins, R., Edwards, A., Ehn, M., Flodin, S., Flores, A., Graslund, S., Hammarstrom, M., Kotenyova, T., Nilsson-Ehle, P., Nordlund, P., Nyman, T., Sagemark, J., Stenmark, P., Sundstrom, M., Van Den Berg, S., Weigelt, J., Holmberg-Schiavone, L., Persson, C. and Hallberg, B.M. (2006) Structural Genomics Consortium. Journal: To be Published. (2GK9.pdb)
17. Chamberlain, P.P., Qian, X., Stiles, A.R., Cho, J., Jones, D.H., Lesley, S.A., Grabau, E.A., Shears, S.B. and Spraggon, G. (2007) Integration of inositol phosphate signaling pathways via human ITPK1. *J. Biol. Chem. 282*, 28117-28125. (2QB5.pdb)
18. Herzberg, O., Chen, C.C., Kapadia, G., McGuire, M., Carroll, L.J., Noh, S.J. and Dunaway-Mariano, D. (1996) Swiveling-domain mechanism for enzymatic phosphotransfer between remote reaction sites. *Proc.Natl.Acad.Sci.USA 93*, 2652-2657. (1DIK.pdb)
19. Andreassi, J.L., Vetting, M.W., Bilder, P.W., Roderick, S.L. and Leyh, T.S. (2009) Structure of the ternary complex of phosphomevalonate kinase: the enzyme and its family. *Biochemistry 48***,** 6461-6468. (3GON.pdb)
20. Kraft, L., Sprenger, G.A. and Lindqvist, Y. Conformational changes during the catalytic cycle of gluconate kinase as revealed by X-ray crystallography. (2002) *J. Mol. Biol. 318***,** 1057-1069. (1KO5.pdb)
21. Harrison, D.H., Runquist, J.A., Holub, A. and Miziorko, H.M. (1998) The crystal structure of phosphoribulokinase from Rhodobacter sphaeroides reveals a fold similar to that of adenylate kinase. *Biochemistry 37*, 5074-5085. (1A7J.pdb)
22. Segura-Pena, D., Lutz, S., Monnerjahn, C., Konrad, M., and Lavie, A. (2007) Binding of ATP to TK1-like enzymes is associated with a conformational change in the quaternary structure. *J. Mol. Biol. 369***,** 129-141. (2ORW.pdb)
23. Chetnani, B., Das, S., Kumar, P., Surolia, A. and Vijayan, M. (2009) Mycobacterium tuberculosis pantothenate kinase: possible changes in location of ligands during enzyme action. *Acta Crystallogr.,Sect.D 65*, 312-325. (2ZS9.pdb)
24. Kosinska, U., Stenmark, P., Arrowsmith, C., Berglund, H., Busam, R., Collins, R., Edwards, A., Ericsson, U.B., Flodin, S., Flores, A., Graslund, S., Hammarstrom, M., Hallberg, B.M., Holmberg Schiavone, L., Hogbom, M., Johansson, I., Karlberg, T., Kotenyova, T., Moche, M., Nilsson, M.E.P., Nyman, T., Ogg, D., Persson, C., Sagemark, J., Sundstrom, M., Uppenberg, J., Uppsten, M., Thorsell, A.G., Van Den Berg, S., Weigelt, J., Welin, M and Nordlund, P. (2007) Structure of Human Uridine-Cytidine Kinase 1. Journal**:** To be Published. (2UVQ.pdb)
25. Gu, Y., Reshetnikova, L., Li, Y.,   Wu, Y.,   Yan, H.,   Singh, S.,   Ji, X. (2002) Crystal structure of shikimate kinase from Mycobacterium tuberculosis reveals the dynamic role of the LID domain in catalysis. *J. Mol. Biol. 319*, 779-789. (1L4U.pdb)
26. Kim, S.G.,   Cavalier, M.,   El-Maghrabi, M.R.,   Lee, Y.H. (2007) A direct substrate-substrate interaction found in the kinase domain of the bifunctional enzyme, 6-phosphofructo-2-kinase/fructose-2,6-bisphosphatase. *J. Mol. Biol. 370*, 14-26. (2I1V.pdb)
27. Welin, M.,   Wang, L.,   Eriksson, S.,   Eklund, H. (2007) Structure-function analysis of a bacterial deoxyadenosine kinase reveals the basis for substrate specificity. *J. Mol. Biol. 366*, 1615-1623. (2JAS.pdb)
28. Egeblad-Welin, L., Sonntag, Y., Eklund, H. and Munch-Petersen, B. (2007) Functional studies of active-site mutants from Drosophila melanogaster deoxyribonucleoside kinase. Investigations of the putative catalytic glutamate-arginine pair and of residues responsible for substrate specificity. FEBS J. **274:** 1542-1551. (2JCS.pdb)
29. Zhu, Y.,   Huang, W.,   Lee, S.S.,   Xu, W. (2005) Crystal structure of a polyphosphate kinase and its implications for polyphosphate synthesis. *Embo Rep. 6***,** 681-687. (1XDP.pdb)
30. Bunkoczi, G., Filippakopoulos, P., Jansson, A., Longman, E., Von Delft, F., Edwards, A., Arrowsmith, C., Sundstrom, M., Weigelt, J. and Knapp, S. (2009) Structure of Adenylate Kinase 1 in Complex with P1, P4-Di(Adenosine)Tetraphosphate. Structural Genomics Consortium. Journal: To be Published. (2C95.pdb)
31. Schlichting, I. And Reinstein, J. (1999 pH influences fluoride coordination number of the AlFx phosphoryl transfer transition state analog. *Nat. Struct. Biol. 6*, 721-723. (1QF9.pdb)
32. Pattanayek, R., Williams, D.R., Pattanayek, S., Xu, Y., Mori, T., Johnson, C.H., Stewart, P.L. and Egli, M. (2006) Analysis of KaiA-KaiC protein interactions in the cyano-bacterial circadian clock using hybrid structural methods. *Embo J. 25*, 2017-2028. (2GBL.pdb)
33. Tari, L.W., Matte, A., Goldie, H. and Delbaere, L.T. (1997) Mg(2+)-Mn2+ clusters in enzyme-catalyzed phosphoryl-transfer reactions. *Nat. Struct. Biol. 4*, 990-994. (1AQ2.pdb)
34. Petit, P., Lagarde, A., Boutin, J.A., Ferry, G. and Vuillard, L. (2009) Effect of modulators on the structure and function of human glucokinase. Journal: To be Published. (3FGU.pdb)
35. Ose, T., Kitamura, M., Okuyama, M., Mori, H., Kimura, A., Watanabe, N., Yao, M. and Tanaka, I. (2009) Crystal Structure of Alpha-Xylosidase from Escherichia coli. Journal**:** To be Published. (1WE5.pdb)
36. Kotaka, M., Ren, J., Lockyer, M., Hawkins, A.R. and Stammers, D.K. (2006) Structures of R- and T-state Escherichia coli aspartokinase III. Mechanisms of the allosteric transition and inhibition by lysine. *J. Biol. Chem. 281*, 31544-3152. (2J0W.pdb)
37. Gil-Ortiz, F., Ramon-Maiques, S., Fita, I. and Rubio, V. (2003) The course of phosphorus in the reaction of N-acetyl-L-glutamate kinase, determined from the structures of crystalline complexes, including a complex with an AlF(4)(-) transition state mimic. J. Mol. Biol. **331:** 231-244. (1OH9.pdb)
38. Marco-Marin, C., Gil-Ortiz, F. and Rubio, V. (2005) The crystal structure of Pyrococcus furiosus UMP kinase provides insight into catalysis and regulation in microbial pyrimidine nucleotide biosynthesis. *J. Mol. Biol. 352*, 438-354. (2BRI.pdb)
39. Evans, P.R., Farrants, G.W. and Hudson, P.J. (1981) Phosphofructokinase: structure and control. *Philos. Trans. R. Soc. London, Ser.B 293*, 53-62. (4PFK.pdb)
40. McNae, I.W., Martinez-Oyanedel, J., Keillor, J.W., Michels, P.A., Fothergill-Gilmore, L.A. and Walkinshaw, M.D. (2009) The crystal structure of ATP-bound phosphofructokinase from Trypanosoma brucei reveals conformational transitions different from those of other phosphofructokinases. *J. Mol. Biol. 385*, 1519-1533. (3F5M.pdb)
41. Liu, J., Lou, Y., Yokota, H., Adams, P.D., Kim, R. and Kim, S.H. (2005) Crystal structures of an NAD kinase from Archaeoglobus fulgidus in complex with ATP, NAD, or NADP. *J. Mol. Biol. 354*, 289-303. (1Z0S.pdb)
42. Crystal structure of Possible 1-phosphofructokinase (EC 2.7.1.56) (tm0828) from THERMOTOGA MARITIMA at 2.46 A resolution. Joint Center for Structural Genomics (JCSG). Journal: To be published. (2AJR.pdb)
43. Seetharaman, J., Lew, S., Wang, D., Neely, H., Janjua, K., Cunningham, K., Owens, L.,   Xiao, R., Liu, J., Baran, M.C., Acton, T.B., Rost, B., Montelione, G.T., Hunt, J.F. and Tong, L. (2009) Crystal structure of Pyrophosphate-dependent phosphofructokinase from Marinobacter aquaeolei, Northeast Structural Genomics Consortium Target MqR88. Journal: To be Published. (3K2Q.pdb)
44. Miller, D.J., Jerga, A., Rock, C.O. and White, S.W. (2008) Analysis of the Staphylococcus aureus DgkB structure reveals a common catalytic mechanism for the soluble diacylglycerol kinases. *Structure 16*, 1036-1046. (2QV7.pdb)
45. Morgan, H. P., McNae, I. W., Nowicki, M. W., Hannaert, V., Michels, P. A. M., Fothergill-Gilmore, L. A., and Walkinshaw, M. D. (2010) Allosteric mechanism of pyruvate kinase from *Leishmania mexicana* uses a rock and lock model. *J.Biol.Chem. 285*, 12892-12898. (3HQQ.pdb)
46. Nocek, B., Cuff, M., Volkart, L., and Joachimiak, A crystal structure of fructokinase from *Bacillus subtilis* complexed with ADP. To be Published. (3EPQ.pdb)
47. Sigrell, J. A., Cameron, A. D., Jones, T. A., and Mowbray, S. L. (1998) Structure of *Escherichia coli* ribokinase in complex with ribose and dinucleotide determined to 1.8 Å resolution: insights into a new family of kinase structures. *Structure 6,* 183-193. (1RKD.pdb)
48. Reddy, M. C. M., Palaninathan, S. K., Shetty, N. D., Owen, J. L., Watson, M. D., and Sacchettini, J. C. (2007) High resolution crystal structures of *Mycobacterium tuberculosis* adenosine kinase: insights into the mechanism and specificity of this novel prokaryotic enzyme. *J.Biol.Chem. 282*, 27334-27342. (2PKN.pdb)
49. Bagaria, A., Kumaran, D., Burley, S. K., and Swaminathan, S. Crystal structure of pyridoxal kinase from *Lactobacillus plantarum* in complex with ATP. Journal**:** To be Published. (3IBQ.pdb)
50. Satyanarayana, L., Burley, S. K., and Swaminathan, S. Crystal Structure of a putative 2-Keto-3-deoxygluconate kinase from *Enterococcus faecalis*. Journal**:** To be Published. (3KTN.pdb)
51. Jeyakanthan, J., Kuramitsu, S., and Yokoyama, S. Structure of hydroxyethylthiazole kinase protein from *Pyrococcus Horikoshii* Ot3. Journal**:** To be Published. (3HPD.pdb)
52. Joint Center for Structural Genomics (JCSG). Crystal structure of possible 1-phosphofructokinase (EC 2.7.1.56) (tm0828) from *Thermotoga maritina* at 2.46 Å resolution. Journal**:** To be Published. (2AJR.pdb)
53. Miallau, L., Hunter, W. N., Mcsweeney, S. M., and Leonard, G. A. (2007) Structures of *Staphylococcus aureus* d-tagatose-6-phosphate kinase implicate domain motions in specificity and mechanism. *J.Biol.Chem. 282*, 19948. (2JG1.pdb, 2JGV.pdb)
54. Kochinyan, S., Brown, G., Skarina, T., Singer, A. U., Wong, A. H. Y., Cuff, M. E., Guixe, V., Merino, F., Savchenko, A., Yakunin, A. F., and Jia, Z. Crystal structure of an ADP-dependent 6-phosphofructokinase from *Pyrococcus horikoshii* OT3. Journal**:** To be Published. (3DRW.pdb)
55. Ito, S., Fushinobu, S., Yoshioka, I., Koga, S., Matsuzawa, H., and Wakagi, T. (2001) Structural basis for the ADP-specificity of a novel glucokinase from a hyperthermophilic archaeon. *Structure* *9*, 205-214. (1GC5.pdb)
56. Bagautdinov, B., Kuramitsu, S., Yokoyama, S., Miyano, M., and Tahirov, T. H. Crystal Structure analysis of phosphomethylpyrimidine Kinase (*ThiD*) from *Thermus Thermophilus* Hb8. To be Published. (1UB0.pdb)
57. Cheng, G., Bennett, E. M., Begley, T. P., and Ealick, S. E. (2002) Crystal structure of 4-amino-5-hydroxymethyl-2-methylpyrimidine phosphate kinase from *Salmonella typhimurium* at 2.3 Å resolution. *Structure* *10*, 225-235. (1JXH.pdb)
58. Liu, J. Y., Timm, D. E., Hurley, T. D. (2006) Pyrithiamine as a substrate for thiamine pyrophosphokinase. *J.Biol.Chem.* *281***,** 6601-6607. (2F17.pdb)
59. Rajashankar, K. R., Kniewel, R., Solorzano, V., and Lima, C.D. Glycerate kinase from *Neisseria meningitidis* (serogroup A). Journal**:** To be Published. (1TO6.pdb)
60. Schwarzenbacher, R., McMullan, D., Krishna, S. S., Xu, Q., Miller, M. D., Canaves, J. M., Elsliger, M. A., Floyd, R., Grzechnik, S. K., Jaroszewski, L., Klock, H. E., Koesema, E., Kovarik, J. S., Kreusch, A., Kuhn, P., McPhillips, T. M., Morse, A. T., Quijano, K., Spraggon, G., Stevens, R. C., van den Bedem, H., Wolf, G., Hodgson, K. O., Wooley, J., Deacon, A. M., Godzik, A., Lesley, S. A., and Wilson, I. A. (2006) Crystal structure of a glycerate kinase (TM1585) from *Thermotoga maritima* at 2.70 Å resolution reveals a new fold. *Proteins* *65*, 243-248. (2B8N.pdb)
61. Osipiuk, J., Zhou, M., Holzle, D., Anderson, W., and Joachimiak, A. X-ray crystal structure of putative glycerate kinase 2 from *Salmonella typhimurium* LT2. Journal**:** To be Published. (3CWC.pdb)
62. Aleshin, A. E., Kirby, C., Liu, X., Bourenkov, G. P., Bartunik, H. D., Fromm, H. J., and Honzatko, R. B. (2000) Crystal structures of mutant monomeric hexokinase I reveal multiple ADP binding sites and conformational changes relevant to allosteric regulation, *J.Mol.Biol. 296***,** 1001-1015. (1DGK.pdb)
63. Petit, P., Lagarde, A., Boutin, J. A., Ferry, G., and Vuillard, L. Effect of modulators on the structure and function of human glucokinase. Journal**:** To be Published. (3FGU.pdb)
64. Satyanarayana, L., Burley, S. K., and Swaminathan, S. Crystal structure of fructokinase with bound ATP from *Xylella fastidiosa*. Journal**:** To be Published. (3KLI.pdb)
65. Grueninger, D., and Schulz, G. E. (2006) Structure and reaction mechanism of l-rhamnulose kinase from *Escherichia coli*. *J.Mol.Biol. 359***,** 787. (2CGJ.pdb)
66. Zhang, Z., Burley, S. K., and Swaminathan, S. The crystal structure of xylulose kinase from *Lactobacillus acidophilus*. Journal**:** To be Published. (3LL3.pdb)
67. Agarwal, R., Burley, S. K., and Swaminathan, S. Crystal structure of ribulokinase from *Bacillus halodurans*. Journal**:** To be Published. (3JVP.pdb)
68. Zhang, Z., Burley, S. K., and Swaminathan, S. Crystal structure of xylulokinase from *Chromobacterium violaceum*. Journal**:** To be Published. (3HZ6.pdb)
69. Bystrom, C. E., Pettigrew, D. W., Branchaud, B. P., O`Brien, P., and Remington, S. J. (1999) Crystal structures of *Escherichia coli* glycerol kinase variant S58-->W in complex with nonhydrolyzable ATP analogues reveal a putative active conformation of the enzyme as a result of domain motion. *Biochemistry 38*, 3508-3518. (1BWF.pdb)
70. Yang, K., Strauss, E., Huerta, C., and Zhang, H. (2008) Structural basis for substrate binding and the catalytic mechanism of type III pantothenate kinase. *Biochemistry 47*, 1369-1380. (3BF1.pdb)
71. Weihofen, W. A., Berger, M., Chen, H., Saenger, W., and Hinderlich, S. (2006) Structures of human *N*-acetylglucosamine kinase in two complexes with *N*-acetylglucosamine and with ADP/glucose: insights into substrate specificity and regulation. *J.Mol.Biol. 364*, 388. (2CH6.pdb)
72. Tong, Y., Tempel, W., Nedyalkova, L., Mackenzie, F., and Park, H. W. (2009) Crystal structure of the *N*-acetylmannosamine kinase domain of GNE. *Plos One 4*, e7165-e7165. (3EO3.pdb)
73. Patskovsky, Y., and Almo, S. C. Crystal Structure of *Escherichia coli* putative *N*-acetylmannosamine Kinase. Journal**:** To be Published. (2AA4.pdb)
74. Mukai, T., Kawai, S., Mori, S., Mikami, B., and Murata, K. (2004) Crystal structure of bacterial inorganic polyphosphate/ATP-glucomannokinase. Insights into kinase evolution. J*.Biol.Chem. 279*, 50591-50600. (1WOQ.pdb)
75. Gorrell, A., Lawrence, S. H., and Ferry, J. G. (2005) Structural and kinetic analyses of arginine residues in the active site of the acetate kinase from *Methanosarcina thermophila*. *J.Biol.Chem. 280***,** 10731-10742. (1TUY.pdb)
76. Diao, J., and Hasson, M.S. (2009) Crystal structure of butyrate kinase 2 from *Thermotoga maritima*, a member of the ASKHA superfamily of phosphotransferases. *J.Bacteriol. 191***,** 2521-2529. (1SAZ.pdb)
77. Hartley, A., Glynn, S. E., Barynin, V., Baker, P. J., Sedelnikova, S. E., Verhees, C., de Geus, D., van der Oost, J., Timson, D. J., Reece, R. J., and Rice, D. W. (2004) Substrate specificity and mechanism from the structure of *Pyrococcus furiosus* galactokinase. *J.Mol.Biol. 337***,** 387-398. (1S4E.pdb)
78. Sgraja, T., Alphey, M. S., Ghilagaber, S., Marquez, R., Robertson, M. N., Hemmings, J. L., Lauw, S., Rohdich, F., Bacher, A., Eisenreich, W., Illarionova, V., and Hunter, W. N. (2008) Characterization of *Aquifex aeolicus* 4-diphosphocytidyl-2C-methyl-d-erythritol kinase - ligand recognition in a template for antimicrobial drug discovery. *FEBS J. 275*, 2779. (2V8P.pdb, 2V2Z.pdb)
79. Zhou, T., Daugherty, M., Grishin, N. V., Osterman, A. L., and Zhang, H. (2000) Structure and mechanism of homoserine kinase: prototype for the GHMP kinase superfamily. *Structure Fold. Des. 8*, 1247-1257. (1FWK.pdb)
80. McCulloch, K. M., Kinsland, C., Begley, T. P., and Ealick, S. E. (2008) Structural studies of thiamin monophosphate kinase in complex with substrates and products. *Biochemistry 47*, 3810-3821. (3C9S.pdb)
81. Wang, K. T., Wang, J., Li, L. F., and Su, X. D. (2009) Crystal structures of catalytic intermediates of human selenophosphate synthetase 1. J*.Mol.Biol. 390*, 747-759. (3FD6.pdb)
82. Itoh, Y., Sekine, S. I., Matsumoto, E., Akasaka, R., Takemoto, C., Shirouzu, M., and Yokoyama, S. (2009) Structure of selenophosphate synthetase essential for selenium incorporation into proteins and RNAs. *J.Mol.Biol 385(5)*, 1456-69. (2YYE.pdb)
83. Karthikeyan, S., Zhou, Q., Osterman, A. L., and Zhang, H. (2003) Ligand binding-induced conformational changes in riboflavin kinase: structural basis for the ordered mechanism. *Biochemistry 42*, 12532-12538. (1Q9S.pdb)
84. Bauer, S., Kemter, K., Bacher, A., Huber, R., Fischer, M., and Steinbacher, S. (2003) Crystal structure of *Schizosaccharomyces pombe* riboflavin kinase reveals a novel ATP and riboflavin-binding fold. *J.Mol.Biol. 326*, 1463-1473. (1N06.pdb, 1N08.pdb)
